# Model-Based Algorithms for Detecting Peripheral Artery Disease Using Administrative Data From an Electronic Health Record Data System: Algorithm Development Study

**DOI:** 10.2196/18542

**Published:** 2020-08-19

**Authors:** Elizabeth Hope Weissler, Steven J Lippmann, Michelle M Smerek, Rachael A Ward, Aman Kansal, Adam Brock, Robert C Sullivan, Chandler Long, Manesh R Patel, Melissa A Greiner, N Chantelle Hardy, Lesley H Curtis, W Schuyler Jones

**Affiliations:** 1 Division of Vascular and Endovascular Surgery Duke University School of Medicine Durham, NC United States; 2 Department of Population Health Sciences Duke University School of Medicine Durham, NC United States; 3 Department of Medicine Duke University School of Medicine Durham, NC United States; 4 Duke Clinical Research Institute Durham, NC United States

**Keywords:** peripheral artery disease, patient selection, electronic health records, cardiology, health data

## Abstract

**Background:**

Peripheral artery disease (PAD) affects 8 to 10 million Americans, who face significantly elevated risks of both mortality and major limb events such as amputation. Unfortunately, PAD is relatively underdiagnosed, undertreated, and underresearched, leading to wide variations in treatment patterns and outcomes. Efforts to improve PAD care and outcomes have been hampered by persistent difficulties identifying patients with PAD for clinical and investigatory purposes.

**Objective:**

The aim of this study is to develop and validate a model-based algorithm to detect patients with peripheral artery disease (PAD) using data from an electronic health record (EHR) system.

**Methods:**

An initial query of the EHR in a large health system identified all patients with PAD-related diagnosis codes for any encounter during the study period. Clinical adjudication of PAD diagnosis was performed by chart review on a random subgroup. A binary logistic regression to predict PAD was built and validated using a least absolute shrinkage and selection operator (LASSO) approach in the adjudicated patients. The algorithm was then applied to the nonsampled records to further evaluate its performance.

**Results:**

The initial EHR data query using 406 diagnostic codes yielded 15,406 patients. Overall, 2500 patients were randomly selected for ground truth PAD status adjudication. In the end, 108 code flags remained after removing rarely- and never-used codes. We entered these code flags plus administrative encounter, imaging, procedure, and specialist flags into a LASSO model. The area under the curve for this model was 0.862.

**Conclusions:**

The algorithm we constructed has two main advantages over other approaches to the identification of patients with PAD. First, it was derived from a broad population of patients with many different PAD manifestations and treatment pathways across a large health system. Second, our model does not rely on clinical notes and can be applied in situations in which only administrative billing data (eg, large administrative data sets) are available. A combination of diagnosis codes and administrative flags can accurately identify patients with PAD in large cohorts.

## Introduction

Lower extremity peripheral artery disease (PAD) is a prevalent chronic vascular condition that is estimated to affect over 200 million patients globally [1]. Although most patients with PAD are asymptomatic, more severe disease is associated with negative health and quality of life effects including claudication (leg pain caused by insufficient blood flow), ischemia (blood flow insufficient to meet the extremity’s metabolic demands), and tissue loss from small wounds that worsen without adequate blood for healing. Severe ischemia with enlarging or infected wounds can require amputation [1,2]. Given the morbidity and mortality burden of PAD, investigation of novel therapies and implementation efforts is an ongoing necessity.

Improvement in the quality of PAD treatment and research requires correct and efficient identification of patients who truly have the disease. Although prospective studies can confirm patients’ diagnoses through multiple methods, studies that rely on the review of electronic health records (EHRs) or billing claims are limited to preexisting data. Computable phenotypes based on billing codes are sufficient to identify affected patients for many conditions, but for others, current diagnosis codes do not adequately differentiate the condition of interest from other related conditions [3,4]. PAD detection algorithms using administrative code sets, such as combinations of International Classification of Diseases, Ninth Revision, Clinical Modification (ICD-9-CM) diagnosis codes or Current Procedural Terminology (CPT) procedure codes, have been shown to be relatively inaccurate compared to diagnostic “gold standards” such as the ankle-brachial index, especially when applied beyond patients seen in a vascular laboratory or clinic setting [5-8].

Our initial attempt to identify patients with PAD within a large US academic health system using billing diagnosis codes had a very low positive predictive value. In this paper, we describe the two-staged “learning” approach that we adopted by first determining the PAD status for a random sample of the initially selected patients; training and validating a model using that patient set; and then scoring the remaining patients from the initial patient query to identify charts with a high likelihood of PAD to review for model validation and PAD cohort inclusion. The goal of this research was to develop and validate a model-based algorithm to accurately detect patients with peripheral artery disease using diagnostic billing codes and administrative information available in the EHRs data system.

## Methods

### Data Source and Study Population

The study population was selected using a query to Duke Enterprise Data Unified Content Explorer (DEDUCE). DEDUCE interfaces with and supports queries of the EHR data repository for all patients seen within the Duke University Health System (DUHS), an integrated health system that includes 3 hospitals and a large number of outpatient clinical offices in the Raleigh-Durham region of North Carolina. To be eligible for inclusion in this study, patients needed to have had at least one clinical encounter at DUHS resulting in one or more PAD-related diagnosis codes between January 1, 2015, and March 31, 2016. This study period was chosen in part because it included the period during which the ICD-9-CM to ICD-10-CM (10th Revision) transition occurred, thereby facilitating incorporation of codes from both systems into our algorithm. Encounter-level EHRs were obtained for all clinical encounters during the study period, including hospital admissions, emergency department visits, and outpatient clinic visits. This research was approved by the Duke University Institutional Review Board (protocol ID number Pro00075637).

### Selection of Diagnosis Codes, Procedure Codes, and Other Administrative Data Flags

Our initial list of diagnosis codes related to lower extremity PAD (including peripheral vascular disease, atherosclerosis, diabetes with peripheral circulatory disorders, lower extremity ulcers, arterial thromboembolism, and gangrene) contained 31 ICD-9-CM and 375 ICD-10-CM diagnosis codes (Multimedia Appendix 1). The ICD-9-CM codes used in this study were drawn from cohort eligibility criteria or outcome definitions from prior studies of PAD [9,10], as well as from clinician review of the ICD-9-CM classification system. ICD-10-CM codes were forward- and backward-mapped from ICD-9-CM codes using General Equivalence Mappings (GEMs) and were screened by the clinical team to eliminate spurious mappings [11]. The mapped corresponding ICD-9 and ICD-10 codes were included as separate flags. However, there were two codes (ICD-9-CM 443.9 and ICD-10-CM I73.9 for “Peripheral vascular disease, unspecified”) that were grouped into a single flag because the terminology for this code did not change with the ICD-10-CM transition. In addition, nearly half of all patients in the study population had one or both of those codes (443.9 or I73.9) present during the study period. There were 247 PAD-related codes that were not detected for any patients, and an additional 50 codes that were used for only 1 or 2 patients; these codes were removed from the analysis, leaving 108 diagnosis code flags including the combined flag for 443.9/I73.9.

Additionally, 4 indicator variables were created to increase the likelihood that a PAD-related diagnosis code indicated true PAD, rather than an encounter devoting to “ruling out” PAD. Two were procedure code-based: one for having any revascularization procedure and another for having any diagnostic imaging code associated with an encounter with a PAD-related diagnosis code. We selected ICD-9-PCS, ICD-10-PCS, and CPT procedure codes based on prior literature and clinical expertise (Multimedia Appendix 2) [12,13]. Revascularization procedures included codes for atherectomy, angioplasty, dilation, bypass, replacement, or supplementation procedures related to the lower extremity arteries. Diagnostic studies included noninvasive hemodynamic studies, ultrasound, magnetic resonance imaging, computed tomography angiography, and catheter-based angiography.

Finally, we also derived two indicator variables based on other administrative information contained within the EHR. One was a flag for having two or more encounters with a PAD-related diagnosis code within the study period. The other was a flag for encounters associated with PAD codes in which the primary physician was listed as “Cardiology,” “Vascular Surgery,” “Cardiovascular Medicine,” “Interventional Radiology,” “Podiatry,” or “Wound Care” (the most common provider types who frequently care for patients with PAD).

### Chart Abstraction Process, Model Development, and Validation

Chart abstraction was necessary for the larger PAD outcomes study that this project was a part of because there are potential confounders of the associations between patient characteristics and clinical outcomes that must be obtained through review of clinical data. It was impractical to abstract data from and confirm the very large number of potential patients with PAD identified from the initial billing diagnosis codes. Instead, we took a two-staged “learning” approach to abstraction by first reviewing charts from a random subgroup of patients and then using this PAD-adjudicated subgroup to model which of the diagnosis and administrative flags were most predictive of true PAD diagnosis. We then used the probabilities generated from this model to decide which of the remaining patients’ records to abstract.

**Figure 1:**
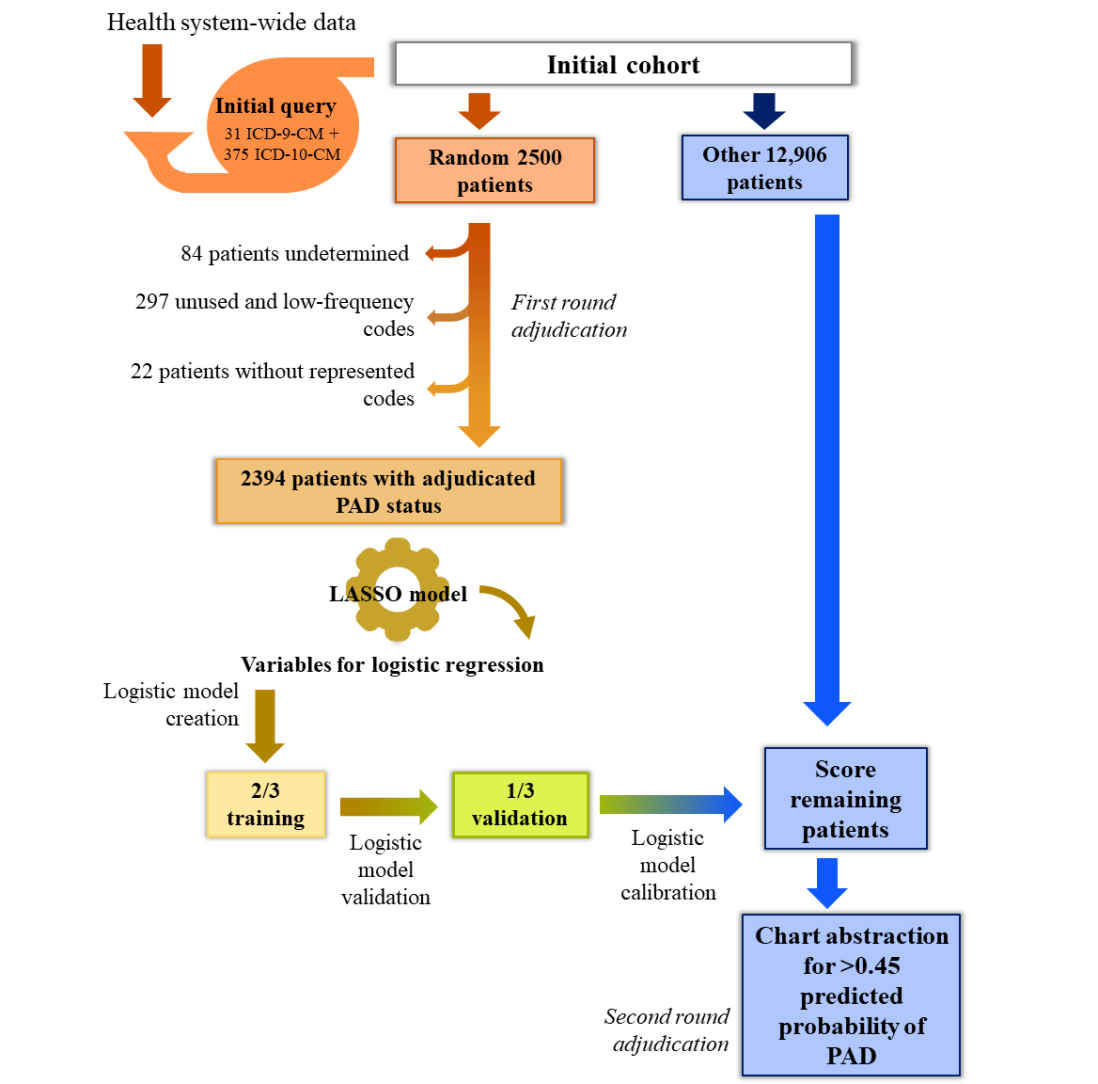
Overview of the chart abstraction and analysis process. LASSO: least absolute shrinkage and selection operator; PAD: peripheral artery disease.

Chart review was performed in accordance with a written manual to standardize abstraction. There were 6 medical abstractors in total, and each reviewer was trained to complete the forms completely. When discordant information, inconclusive data, or uncertainty remained after initial review, the file was marked and the senior author (WSJ) reviewed the file and made a final determination. In the first stage of abstraction, we reviewed charts to adjudicate PAD status for a random sample of 2500 patients from the original cohort (Figure 1). PAD was confirmed using either ankle-brachial index (ABI), history of prior revascularization, or lower extremity amputation for an indication of symptomatic PAD. ABI 0.9 or ABI 1.4 in either limb was diagnostic of PAD, and toe pressures were used if lower extremity vessels were noncompressible. Revascularization procedures performed between January 1, 2010 and the index visit date during the study period within DUHS were considered to be prior revascularizations.

To avoid overfitting the prediction model, we used the least absolute shrinkage and selection operator (LASSO) approach to reduce the number of variables [14]. The starting point of the LASSO model included the binary flags for each diagnostic code, as well as for revascularization procedures, diagnostic imaging, specialist provider, and having ≥2 PAD encounters. Using the chart abstraction PAD status determination as the “ground truth,” we fit the LASSO logistic regression model with all adjudicated patients. The LASSO model was performed using the SAS (SAS Institute Inc) HPGENSELECT procedure using the Bayesian information criterion as the selection criterion, and setting the stop criterion to “none.” The LASSO-reduced variable list was stored for use in the next stage of analysis.

Adjudicated patients were then randomly partitioned 2:1 into training and validation sets. Using only the training set, we fit a logistic regression model with the LASSO-reduced variable list, stored the model coefficients, estimated the C statistic, and produced a receiver operating characteristic (ROC) curve. We then applied the stored model coefficients to score the patients in the validation set and reestimated the C statistic. To assess model calibration, we divided both the training and validation sets into deciles of predicted probability and plotted the proportion of adjudicated true PAD within each decile.

After confirming that the model was performing similarly in both the training and validation sets, we recombined the sets and fit a final logistic model using the LASSO-reduced variable list and all adjudicated patients to obtain the final trained coefficient estimates. Using the predicted probabilities from the final trained logistic model, we evaluated potential discrimination thresholds to classify true presence of PAD.

We then scored the remaining, unadjudicated patients from the original data query using the final model coefficients. Patients with a predicted probability of ≥45% of truly having PAD were then included in the second round of chart abstraction. This threshold was chosen to favor sensitivity and was based on examination of both the ROC curve and the distribution of the predicted probabilities among these patients. To further validate the model performance, we also evaluated the concordance between the predicted PAD probability and actual PAD presence for each level of predicted probability.

## Results

### Overview

In the initial data pull from the DUHS EHRs data repository, we identified 15,406 patients who had ≥1 clinical encounter within the health system during the period from January 1, 2015, through March 31, 2016, that was coded with one of the 406 PAD-related ICD-9-CM and ICD-10-CM diagnosis codes. Of the 2500 patients who were randomly selected for the first round of chart abstraction, 2416 had a definitive “yes” or “no” decision adjudicated. The remaining 84 patients were considered “undetermined” due to insufficient evidence in the charts, and were removed from the cohort.

### Initial Code Inclusion and Exclusion Decisions

We began the analysis with 406 PAD-related ICD diagnosis codes (31 ICD-9-CM, 375 ICD-10-CM). Of those 406 codes, there were 247 codes that were not assigned during the study period to any of the 2416 clinically adjudicated patients. Additionally, 35 codes were assigned to only 1 patient, and 15 codes were assigned to only 2 patients during the study period. One of the most common diagnoses was “Peripheral vascular disease, unspecified,” which is 443.9 in ICD-9-CM and I73.9 in ICD-10-CM; 1190 (49.7%) of all patients had either one or both of those codes present during the study period. We grouped these two codes into a single flag because of their prevalence and because the terminology for this code did not change with the ICD-10-CM transition. Prior to LASSO modeling, we removed 297 unused and very low frequency (1-2 uses) diagnosis codes and combined the flag for 443.9/I73.9, leaving 108 diagnosis code flags to be included in the LASSO model. In addition, 22 patients who no longer had any of the retained diagnosis codes were removed, leaving an analysis cohort of 2394 patients with adjudicated yes/no PAD status (Figure 1). Among these 2394 patients, only 780 (32.6%) were adjudicated as having confirmed PAD (Table 1; baseline characteristics by training versus validation roles available in Multimedia Appendix 3).

**Table 1:** Baseline characteristics of initially-adjudicated patients by confirmed peripheral artery disease status.

Demographics			Patients without confirmed peripheral artery disease (n=1614)		Patients with confirmed peripheral artery disease (n=780)		*P* value	
Age (years), mean (SD)			66.9 (15.0)		69.8 (10.9)		.001	
Gender (male), n (%)			824 (51.1)		464 (59.5)		<.001	
**Race, n (%)**								<.001
	White	1161 (71.9)		464 (59.5)		N/A^a^		
	Black/African American	380 (23.5)		252 (32.3)		N/A		
	Other	73 (4.5)		64 (8.2)		N/A		

^a^N/A: not applicable.

### Model Construction and Evaluation

We first assessed multicollinearity by fitting a linear regression model and evaluating the variance inflation factor for each of the 108 retained diagnosis flags and the 4 other indicator variables. Most of the variance inflation factor values were below 1.5, and the maximum VIF was 2.85, indicating that the variables in the model were sufficiently noncollinear to proceed, using a rule of thumb of <3.

We then entered the 108 diagnosis code flags and the 4 administrative flags for revascularization, diagnostic testing, specialist service, and ≥2 PAD-related encounters into the LASSO logistic regression prediction model. This yielded 15 flags for inclusion, including all 4 administrative flags and 11 diagnosis code flags.

Using the 15 LASSO-selected variables, we fit another logistic regression model to the adjudicated training set (2/3 partition, n=1604). Odds ratios and 95% confidence intervals from this training model are presented in Table 2.

**Figure 2:**
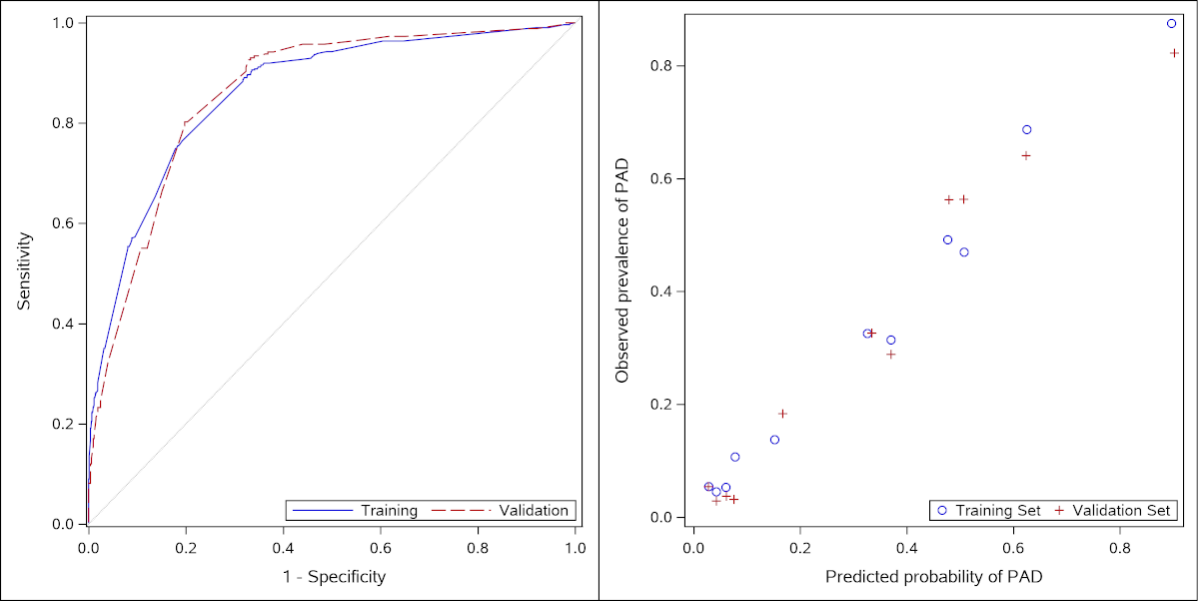
Comparison of results from training and validation sets. Left panel: receiver operating characteristic curves for training and validation sets. Right panel: Comparison of deciles of predicted probabilities in training set versus validation set. PAD: peripheral artery disease.

In the training set, the C statistic was 0.8618 (95% CI 0.8427-0.8810). We then applied the model coefficients derived from the training set to score the observations in the adjudicated validation set (1/3 partition, n=790). In the validation set, the C statistic was 0.8618 (95% CI 0.8352-0.8884). Figure 2 displays the ROC curves for both the training and validation sets. Additionally, we ranked both the training and validation sets into deciles of predicted probability and plotted the relationship between the mean predicted probability in each decile to the observed prevalence of confirmed PAD in that decile (Figure 2). Overall, it appeared that the model derived from the training set fit the validation data equally well.

Finally, we refit the model using all 2394 PAD-adjudicated patients to obtain the final odds ratios, which are displayed in Table 2. The C statistic for the area under the ROC curve for this final model was 0.8618 (95% CI 0.8463-0.8774). We then generated a classification table to assess the impact of potential thresholds of predicted probability on the discrimination measures. At a threshold of predicted probability ≥0.45, the estimated sensitivity was 75.3% and the estimated specificity was 81.7%, with an estimated positive predictive value of 66.5% and negative predictive value of 87.2%.

**Figure 3:**
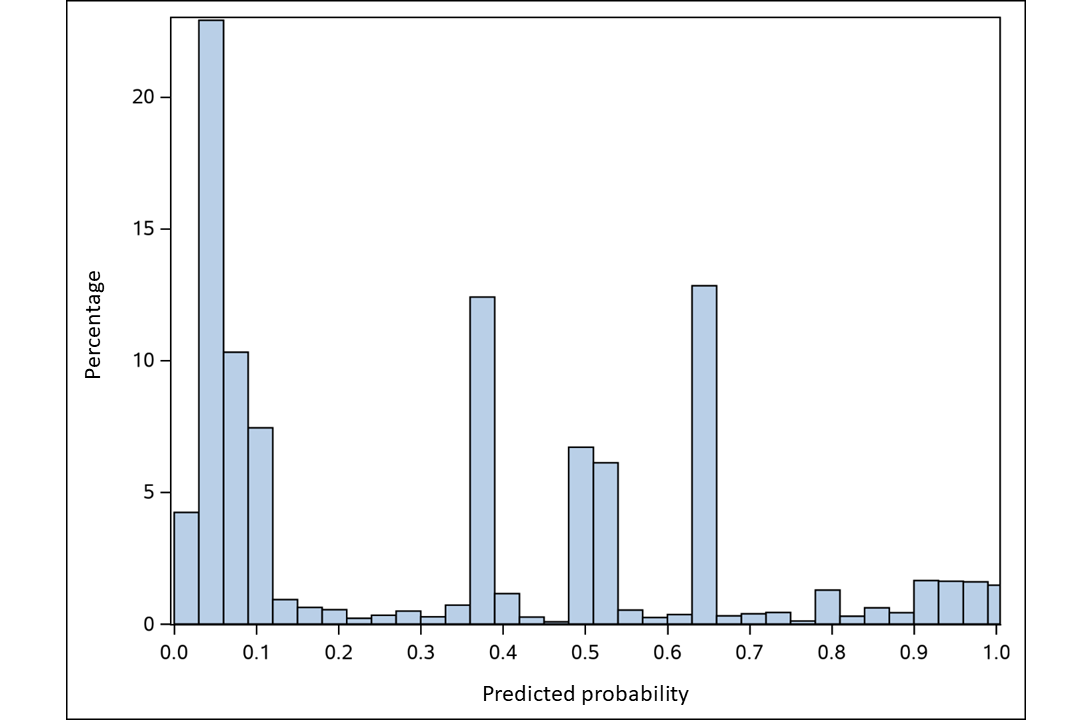
Histogram of predicted PAD probabilities in the remaining unadjudicated patients using the final model coefficients. The distribution of predicted PAD probabilities contributed to a chosen probability threshold of 0.45 for second round chart adjudication. PAD: peripheral artery disease.

**Figure 4:**
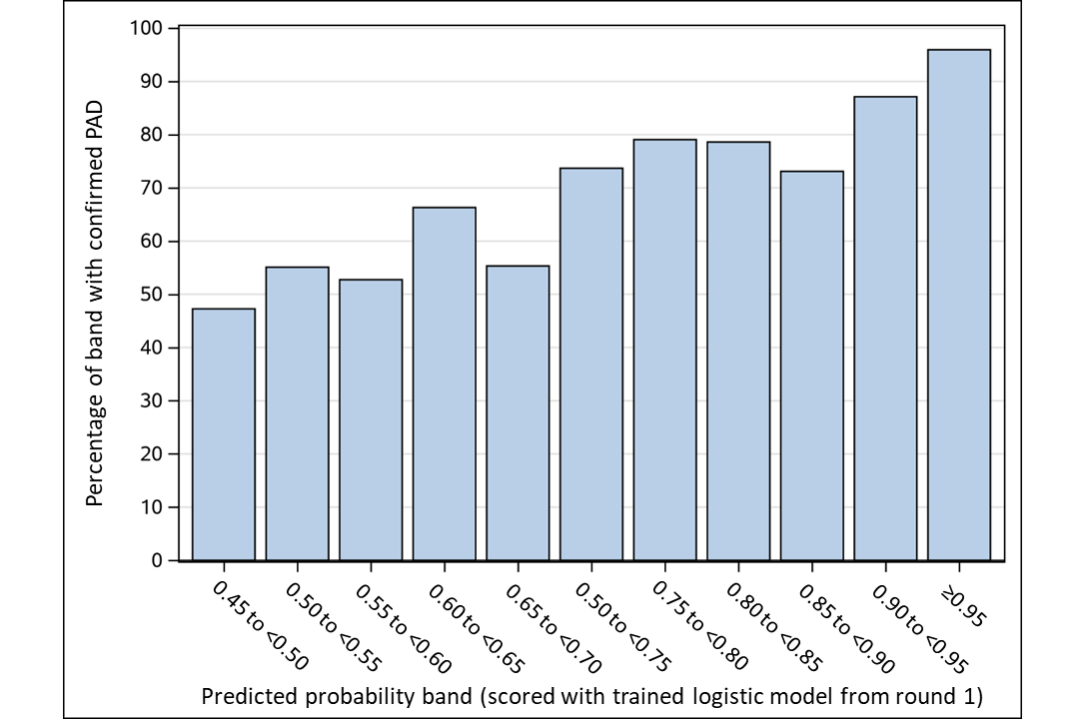
Model performance. Proportion of patients who were selected for the second round of abstraction who were confirmed to have PAD via abstraction, by bands of the predicted probabilities obtained with the trained logistic model. PAD: peripheral artery disease.

We then applied the final model coefficients to score the remaining 12,801 patients from the original data pull. Of these patients, 4753 (37.1%) had a predicted PAD probability of ≥0.45 (Figure 3). PAD status was definitively adjudicated in 4493 patients and 260 patients were assigned an “Undetermined” status. Of the 4493 patients, 2981 (66.3%) were confirmed to have PAD. Figure 4 illustrates the proportion of patients who had confirmed PAD at each level of predicted probability.

**Table 2:** Odds ratio estimates and 95% confidence intervals from the training set and the final model using the variables selected in the least absolute shrinkage and selection operator (LASSO) model.

Diagnosis code or flag type	ICD^a^ version	ICD code description or study definition	Training set (n=1604), odds ratio (95% CI)	Final model (n=2394), odds ratio (95% CI)
250.70	9	Diabetes with peripheral circulatory disorders, type II or unspecified type, not stated as uncontrolled	1.81 (0.78-4.24)	1.62 (0.78-3.39)
440.20	9	Atherosclerosis of native arteries of the extremities, unspecified	2.04 (0.79-5.29)	3.62 (1.60-8.19)
440.21	9	Atherosclerosis of native arteries of the extremities with intermittent claudication	6.28 (2.84-13.92)	5.81 (3.07-10.97)
440.23	9	Atherosclerosis of native arteries of the extremities with ulceration	24.18 (4.48-130.45)	13.73 (3.22-58.64)
440.9	9	Generalized and unspecified atherosclerosis	0.73 (0.36-1.49)	0.71 (0.39-1.30)
444.22	9	Arterial embolism and thrombosis of lower extremity	2.14 (0.67-6.78)	4.37 (1.67-11.48)
707.10	9	Ulcer of lower limb, unspecified	0.29 (0.12-0.70)	0.41 (0.21-0.80)
785.4	9	Gangrene	2.59 (0.89-7.48)	2.71 (1.11-6.59)
I702.13	10	Atherosclerosis of native arteries of extremities with intermittent claudication, bilateral legs	103.23 (11.78-904.75)	20.55 (5.97-70.80)
I96	10	Gangrene, not elsewhere classified	2.61 (0.87-7.86)	2.15 (0.84-5.51)
443.9 or I739	9 and 10	Peripheral vascular disease, unspecified	13.28 (9.53-18.50)	14.22 (10.77-18.77)
Specialist	N/A^b^	Any PAD-related specialist during study period	1.64 (1.22-2.19)	1.66 (1.31-2.10)
Revascularization	N/A	Any revascularization procedure during study period	3.38 (1.60-7.14)	2.37 (1.32-4.26)
Diagnostic imaging	N/A	Any PAD-related diagnostic imaging test during study period	1.08 (0.71-1.65)	0.99 (0.70-1.40)
≥2 encounters	N/A	2 PAD-related encounters during study period	1.70 ( 1.26-2.28)	1.86 (1.46-2.36)

^a^ICD: International Classificiation of Diseases.

^b^N/A: not applicable.

## Discussion

### Principal Results

We created a model-based algorithm for detecting PAD because diagnosis codes were an unacceptably low-yield way to find patients with PAD in our health system’s EHRs. Out of 2394 randomly selected patients from our initial query of 15,406 patients with PAD codes, only 780 actually had PAD, a positive predictive value of 32.6%. Faced with the prohibitively labor-intensive process of chart extraction and adjudication for thousands of patients in our initial EHR-identified cohort with low probabilities for true PAD (despite the presence of PAD codes), we attempted to improve our yield using the LASSO approach for selecting administrative codes and flags most predictive of PAD. Our final model included 10 individual ICD-9/10 CM codes, one combined ICD-9+10 CM code, and flags for visits with PAD-related specialists, prior revascularizations, PAD-related diagnostic imaging, and ≥2 PAD-related encounters during the study period. This model had a C statistic of 0.8618. When we applied the full model to the remaining 12,801 patients and abstracted PAD status from those predicted to have a 45% or greater chance of PAD, we found that our yield of true PAD diagnoses tracked with the underlying predicted probability of PAD, as seen in Figure 4. That is, roughly 45% of the patients predicted to have a 45% probability of PAD actually had PAD and approximately 95% of the patients predicted to have a 95% probability of PAD actually had PAD.

We are currently using the cohort derived from our code- and administrative data–based model to analyze patient, provider, and health system factors associated with PAD care and outcomes in our health system. We set a 45% threshold for manual chart abstraction both because of the underlying characteristics of the model and because we wanted to derive a cohort that broadly reflected all patients with PAD in our health system. Depending on a researcher’s goals, the threshold for inclusion or chart abstraction can be adjusted accordingly to favor sensitivity (using lower probability threshold) or specificity (using higher probability threshold) as needed, allowing for more efficient cohort construction. For instance, if the model were applied to a larger population with the intent to find patients for a PAD-related interventional study, a researcher might choose to increase the threshold probability to obtain a more specific though less broadly representative cohort with less manual effort.

### Comparison With Prior Work

The use of diagnostic codes in administrative data sets is an appealing method of identifying patients with PAD, but it can be challenging. Although the use of PAD-associated procedure codes generally is sensitive and specific for the subgroup of patients with PAD undergoing a given procedure, diagnosis codes alone have poor predictive value [6,15]. We believe our model combining administrative data with diagnosis codes offers two main advantages.

First, the training population we used to build the model is representative of all patients with PAD at our institution, regardless of what location they received care in, what care they received, and who provided the care. This has not been true of prior similar efforts, which have used preexisting groups of patients with known PAD status from which to construct their models. For instance, Fan et al [7] designed and tested an administrative code–based algorithm in a population of 22,723 Mayo Clinic patients with PAD codes who underwent ABI testing. Their model, which included diagnosis codes, imaging procedures, and toe amputation, yielded an area under the curve of 0.912 in a test subset of the initial vascular lab population. However, when tested in a community sample, the sensitivity dropped from 85.5% to 68%. Hong et al [8] pooled patients from two prior prospective trials that had collected ABIs to create a cohort of patients with known PAD status from whom to construct various models combining diagnosis and procedure codes. They tested their models’ abilities to find the patients already known to have PAD within administrative data sets, reaching a maximum sensitivity of 34.7%. Bekwelem et al [16] used a similar approach to discriminate between patients with and without critical limb ischemia (a more specific and severe kind of PAD) in a preadjudicated database and reported a maximum sensitivity of 92% by using either diagnosis or procedure codes. They then applied their model to unadjudicated health system data, but never confirmed their findings. We believe that training our model in a cohort containing diverse representations of PAD is a significant benefit for its applicability in multiple circumstances.

Second, we believe that another strength of the algorithm is its use of structured data. Though there have been some reports of natural language processing for PAD cohort identification [16,17], free text is not always available, either in adequate amounts to train an algorithm or at all for a given study population of interest. Examples of free text–limited circumstances include feasibility analyses for future studies, the construction of cohorts for further investigation, and research carried out entirely in an administrative context. Natural language processing approaches also require more time, expertise, and computing resources.

### Limitations

Our approach does have some limitations. To maximize sensitivity, our initial DEDUCE query included a large number of PAD diagnosis codes, some of which were not used for any patient in the DEDUCE cohort or were used for only 1 or 2 patients. This may have driven down our initial positive PAD yield rate. Furthermore, we chose to treat each ICD-CM code as an individual flag because the mapping between ICD-9 and ICD-10 was not entirely concordant except in the instance of 443.9 and I73.9 (unspecified peripheral vascular disease). ICD-10 codes for PAD often specify the disease state followed by a specific affected anatomic location. Rather than treat these codes independently, we could have combined all of the ICD-10 codes with similar disease processes across multiple anatomic locations. This may have increased the likelihood for some ICD codes on the margin to make it into the model. In addition, we did not have access to revascularization procedures prior to 2010, which may have minimally decreased the ability of our model to find patients with PAD. The final and most significant limitation of our approach is that, thus far, we have validated it only internally, and are therefore unsure of how it will perform in different EHRs and health systems. As we look toward deploying this model as part of collaborative research with other institutions, we will need to remain vigilant for signs of model performance degradation. Furthermore, deployment in other health systems will require some level of chart adjudication for validation, the necessary amount of which will be determined on the basis of the threshold chosen, intended cohort use, and initial performance in the new health system.

### Conclusions

We selected all patients from an entire health system with PAD-related diagnosis codes between January 1, 2015, and March 31, 2016. Using a random subset of patients, we constructed a code- and administrative data–based model including 10 individual ICD-9/10 CM flags, one combined ICD-9+10 CM flag, and flags for visits with PAD-related specialists, prior revascularizations, PAD-related diagnostic imaging, and ≥2 PAD-related encounters during the study period. This model had a C statistic of 0.8618. Use of only nonselective PAD diagnosis codes to identify patients for research purposes is unacceptably nonspecific for many studies and should not be done without supplementary methods of cohort confirmation.

## Appendix

Multimedia Appendix 1 ICD-9-CM and ICD-10-CM diagnosis codes included in initial data pull: 31 ICD-9-CM codes, 375 ICD-10-CM codes.

Multimedia Appendix 2 Procedure codes for lower extremity revascularization (ICD-9-CM, ICD-10-PCS, and CPT procedure codes) and procedure codes for diagnostic imaging.

Multimedia Appendix 3 Table of baseline demographics by training versus validation roles.

## Abbreviations

ABI: ankle-brachial index
CPT: Current Procedural Technology
DEDUCE: Duke Enterprise Data Unified Content Explorer
DUHS: Duke University Health System
EHR: electronic health record
ICD-9-CM: International Classification of Diseases, Ninth Revision, Clinical Modification
ICD-10-CM: International Classification of Diseases, Tenth Revision, Clinical Modification
LASSO: least absolute shrinkage and selection operator
PAD: peripheral artery disease
ROC: receiver operating characteristic
