# Supplementary material for: Model-Based Algorithms for Detecting Peripheral Artery Disease Using Administrative Data From an Electronic Health Record Data System: Algorithm Development Study
Source: JMIR Med Inform. 2020 Aug 19;8(8):e18542. doi: 10.2196/18542 (PMC7468640; doi:10.2196/18542)
Supplement: Multimedia Appendix 1 [file medinform_v8i8e18542_app1.docx]

**Appendix 1:**

**ICD-9-CM Diagnosis codes used in the initial cohort query**

| Code Set | Code | Description |
| --- | --- | --- |
| ICD9 | 250.70 | Diabetes with peripheral circulatory disorders, type II or unspecified type, not stated as uncontrolled |
| ICD9 | 250.71 | Diabetes with peripheral circulatory disorders, type I [juvenile type], not stated as uncontrolled |
| ICD9 | 250.72 | Diabetes with peripheral circulatory disorders, type II or unspecified type, uncontrolled |
| ICD9 | 250.73 | Diabetes with peripheral circulatory disorders, type I [juvenile type], uncontrolled |
| ICD9 | 440.0 | Atherosclerosis of aorta |
| ICD9 | 440.20 | Atherosclerosis of native arteries of the extremities, unspecified |
| ICD9 | 440.21 | Atherosclerosis of native arteries of the extremities with intermittent claudication |
| ICD9 | 440.22 | Atherosclerosis of native arteries of the extremities with rest pain |
| ICD9 | 440.23 | Atherosclerosis of native arteries of the extremities with ulceration |
| ICD9 | 440.24 | Atherosclerosis of native arteries of the extremities with gangrene |
| ICD9 | 440.29 | Other atherosclerosis of native arteries of the extremities |
| ICD9 | 440.30 | Atherosclerosis of unspecified bypass graft of the extremities |
| ICD9 | 440.31 | Atherosclerosis of autologous vein bypass graft of the extremities |
| ICD9 | 440.32 | Atherosclerosis of nonautologous biological bypass graft of the extremities |
| ICD9 | 440.4 | Chronic total occlusion of artery of the extremities |
| ICD9 | 440.9 | Generalized and unspecified atherosclerosis |
| ICD9 | 443.9 | Peripheral vascular disease, unspecified |
| ICD9 | 444.22 | Arterial embolism and thrombosis of lower extremity |
| ICD9 | 444.81 | Embolism and thrombosis of iliac artery |
| ICD9 | 444.89 | Embolism and thrombosis of other specified artery |
| ICD9 | 445.02 | Atheroembolism of lower extremity |
| ICD9 | 447.1 | Stricture of artery |
| ICD9 | 459.9 | Unspecified circulatory system disorder |
| ICD9 | 707.10 | Ulcer of lower limb, unspecified |
| ICD9 | 707.11 | Ulcer of thigh |
| ICD9 | 707.12 | Ulcer of calf |
| ICD9 | 707.13 | Ulcer of ankle |
| ICD9 | 707.14 | Ulcer of heel and midfoot |
| ICD9 | 707.15 | Ulcer of other part of foot |
| ICD9 | 707.19 | Ulcer of other part of lower limb |
| ICD9 | 785.4 | Gangrene |

**ICD-10-CM Diagnosis codes used in the initial cohort query**

| Code Set | Code | Description |
| --- | --- | --- |
| ICD10 | E0852 | Diabetes mellitus due to underlying condition with diabetic peripheral angiopathy and gangrene |
| ICD10 | E0952 | Drug or chemical induced diabetes with diabetic peripheral angiopathy with gangrene |
| ICD10 | E1051 | Type 1 diabetes mellitus with diabetic peripheral angiopathy without gangrene |
| ICD10 | E1052 | Type 1 diabetes mellitus with diabetic peripheral angiopathy with gangrene |
| ICD10 | E1059 | Type 1 diabetes mellitus with other circulatory complications |
| ICD10 | E1151 | Type 2 diabetes mellitus with diabetic peripheral angiopathy without gangrene |
| ICD10 | E1152 | Type 2 diabetes mellitus with diabetic peripheral angiopathy with gangrene |
| ICD10 | E1159 | Type 2 diabetes mellitus with other circulatory complications |
| ICD10 | E1351 | Other specified diabetes mellitus with diabetic peripheral angiopathy without gangrene |
| ICD10 | E1352 | Other specified diabetes with diabetic peripheral angiopathy with gangrene |
| ICD10 | E1359 | Other specified diabetes with other circulatory complications |
| ICD10 | I700 | Atherosclerosis of aorta |
| ICD10 | I70201 | Unspecified atherosclerosis of native arteries of extremities, right leg |
| ICD10 | I70202 | Unspecified atherosclerosis of native arteries of extremities, left leg |
| ICD10 | I70203 | Unspecified atherosclerosis of native arteries of extremities, bilateral legs |
| ICD10 | I70208 | Unspecified atherosclerosis of native arteries of extremities, other extremity |
| ICD10 | I70209 | Unspecified atherosclerosis of native arteries of extremities, unspecified extremity |
| ICD10 | I70211 | Atherosclerosis of native arteries of extremities with intermittent claudication, right leg |
| ICD10 | I70212 | Atherosclerosis of native arteries of extremities with intermittent claudication, left leg |
| ICD10 | I70213 | Atherosclerosis of native arteries of extremities with intermittent claudication, bilateral legs |
| ICD10 | I70218 | Atherosclerosis of native arteries of extremities with intermittent claudication, other extremity |
| ICD10 | I70219 | Atherosclerosis of native arteries of extremities with intermittent claudication, unspecified extremity |
| ICD10 | I70221 | Atherosclerosis of native arteries of extremities with rest pain, right leg |
| ICD10 | I70222 | Atherosclerosis of native arteries of extremities with rest pain, left leg |
| ICD10 | I70223 | Atherosclerosis of native arteries of extremities with rest pain, bilateral legs |
| ICD10 | I70228 | Atherosclerosis of native arteries of extremities with rest pain, other extremity |
| ICD10 | I70229 | Atherosclerosis of native arteries of extremities with rest pain, unspecified extremity |
| ICD10 | I70231 | Atherosclerosis of native arteries of right leg with ulceration of thigh |
| ICD10 | I70232 | Atherosclerosis of native arteries of right leg with ulceration of calf |
| ICD10 | I70233 | Atherosclerosis of native arteries of right leg with ulceration of ankle |
| ICD10 | I70234 | Atherosclerosis of native arteries of right leg with ulceration of heel and midfoot |
| ICD10 | I70235 | Atherosclerosis of native arteries of right leg with ulceration of other part of foot |
| ICD10 | I70238 | Atherosclerosis of native arteries of right leg with ulceration of other part of lower right leg |
| ICD10 | I70239 | Atherosclerosis of native arteries of right leg with ulceration of unspecified site |
| ICD10 | I70241 | Atherosclerosis of native arteries of left leg with ulceration of thigh |
| ICD10 | I70242 | Atherosclerosis of native arteries of left leg with ulceration of calf |
| ICD10 | I70243 | Atherosclerosis of native arteries of left leg with ulceration of ankle |
| ICD10 | I70244 | Atherosclerosis of native arteries of left leg with ulceration of heel and midfoot |
| ICD10 | I70245 | Atherosclerosis of native arteries of left leg with ulceration of other part of foot |
| ICD10 | I70248 | Atherosclerosis of native arteries of left leg with ulceration of other part of lower left leg |
| ICD10 | I70249 | Atherosclerosis of native arteries of left leg with ulceration of unspecified site |
| ICD10 | I7025 | Atherosclerosis of native arteries of other extremities with ulceration |
| ICD10 | I70261 | Atherosclerosis of native arteries of extremities with gangrene, right leg |
| ICD10 | I70262 | Atherosclerosis of native arteries of extremities with gangrene, left leg |
| ICD10 | I70263 | Atherosclerosis of native arteries of extremities with gangrene, bilateral legs |
| ICD10 | I70268 | Atherosclerosis of native arteries of extremities with gangrene, other extremity |
| ICD10 | I70269 | Atherosclerosis of native arteries of extremities with gangrene, unspecified extremity |
| ICD10 | I70291 | Other atherosclerosis of native arteries of extremities, right leg |
| ICD10 | I70292 | Other atherosclerosis of native arteries of extremities, left leg |
| ICD10 | I70293 | Other atherosclerosis of native arteries of extremities, bilateral legs |
| ICD10 | I70298 | Other atherosclerosis of native arteries of extremities, other extremity |
| ICD10 | I70299 | Other atherosclerosis of native arteries of extremities, unspecified extremity |
| ICD10 | I70301 | Unspecified atherosclerosis of unspecified type of bypass graft(s) of the extremities, right leg |
| ICD10 | I70302 | Unspecified atherosclerosis of unspecified type of bypass graft(s) of the extremities, left leg |
| ICD10 | I70303 | Unspecified atherosclerosis of unspecified type of bypass graft(s) of the extremities, bilateral legs |
| ICD10 | I70308 | Unspecified atherosclerosis of unspecified type of bypass graft(s) of the extremities, other extremity |
| ICD10 | I70309 | Unspecified atherosclerosis of unspecified type of bypass graft(s) of the extremities, unspecified extremity |
| ICD10 | I70311 | Atherosclerosis of unspecified type of bypass graft(s) of the extremities with intermittent claudication, right leg |
| ICD10 | I70312 | Atherosclerosis of unspecified type of bypass graft(s) of the extremities with intermittent claudication, left leg |
| ICD10 | I70313 | Atherosclerosis of unspecified type of bypass graft(s) of the extremities with intermittent claudication, bilateral legs |
| ICD10 | I70318 | Atherosclerosis of unspecified type of bypass graft(s) of the extremities with intermittent claudication, other extremity |
| ICD10 | I70319 | Atherosclerosis of unspecified type of bypass graft(s) of the extremities with intermittent claudication, unspecified extremity |
| ICD10 | I70321 | Atherosclerosis of unspecified type of bypass graft(s) of the extremities with rest pain, right leg |
| ICD10 | I70322 | Atherosclerosis of unspecified type of bypass graft(s) of the extremities with rest pain, left leg |
| ICD10 | I70323 | Atherosclerosis of unspecified type of bypass graft(s) of the extremities with rest pain, bilateral legs |
| ICD10 | I70328 | Atherosclerosis of unspecified type of bypass graft(s) of the extremities with rest pain, other extremity |
| ICD10 | I70329 | Atherosclerosis of unspecified type of bypass graft(s) of the extremities with rest pain, unspecified extremity |
| ICD10 | I70331 | Atherosclerosis of unspecified type of bypass graft(s) of the right leg with ulceration of thigh |
| ICD10 | I70332 | Atherosclerosis of unspecified type of bypass graft(s) of the right leg with ulceration of calf |
| ICD10 | I70333 | Atherosclerosis of unspecified type of bypass graft(s) of the right leg with ulceration of ankle |
| ICD10 | I70334 | Atherosclerosis of unspecified type of bypass graft(s) of the right leg with ulceration of heel and midfoot |
| ICD10 | I70335 | Atherosclerosis of unspecified type of bypass graft(s) of the right leg with ulceration of other part of foot |
| ICD10 | I70338 | Atherosclerosis of unspecified type of bypass graft(s) of the right leg with ulceration of other part of lower leg |
| ICD10 | I70339 | Atherosclerosis of unspecified type of bypass graft(s) of the right leg with ulceration of unspecified site |
| ICD10 | I70341 | Atherosclerosis of unspecified type of bypass graft(s) of the left leg with ulceration of thigh |
| ICD10 | I70342 | Atherosclerosis of unspecified type of bypass graft(s) of the left leg with ulceration of calf |
| ICD10 | I70343 | Atherosclerosis of unspecified type of bypass graft(s) of the left leg with ulceration of ankle |
| ICD10 | I70344 | Atherosclerosis of unspecified type of bypass graft(s) of the left leg with ulceration of heel and midfoot |
| ICD10 | I70345 | Atherosclerosis of unspecified type of bypass graft(s) of the left leg with ulceration of other part of foot |
| ICD10 | I70348 | Atherosclerosis of unspecified type of bypass graft(s) of the left leg with ulceration of other part of lower leg |
| ICD10 | I70349 | Atherosclerosis of unspecified type of bypass graft(s) of the left leg with ulceration of unspecified site |
| ICD10 | I7035 | Atherosclerosis of unspecified type of bypass grafts of other extremity with ulceration |
| ICD10 | I70361 | Atherosclerosis of unspecified type of bypass graft(s) of the extremities with gangrene, right leg |
| ICD10 | I70362 | Atherosclerosis of unspecified type of bypass graft(s) of the extremities with gangrene, left leg |
| ICD10 | I70363 | Atherosclerosis of unspecified type of bypass graft(s) of the extremities with gangrene, bilateral legs |
| ICD10 | I70368 | Atherosclerosis of unspecified type of bypass graft(s) of the extremities with gangrene, other extremity |
| ICD10 | I70369 | Atherosclerosis of unspecified type of bypass graft(s) of the extremities with gangrene, unspecified extremity |
| ICD10 | I70391 | Other atherosclerosis of unspecified type of bypass graft(s) of the extremities, right leg |
| ICD10 | I70392 | Other atherosclerosis of unspecified type of bypass graft(s) of the extremities, left leg |
| ICD10 | I70393 | Other atherosclerosis of unspecified type of bypass graft(s) of the extremities, bilateral legs |
| ICD10 | I70398 | Other atherosclerosis of unspecified type of bypass graft(s) of the extremities, other extremity |
| ICD10 | I70399 | Other atherosclerosis of unspecified type of bypass graft(s) of the extremities, unspecified extremity |
| ICD10 | I70401 | Unspecified atherosclerosis of autologous vein bypass graft(s) of the extremities, right leg |
| ICD10 | I70402 | Unspecified atherosclerosis of autologous vein bypass graft(s) of the extremities, left leg |
| ICD10 | I70403 | Unspecified atherosclerosis of autologous vein bypass graft(s) of the extremities, bilateral legs |
| ICD10 | I70408 | Unspecified atherosclerosis of autologous vein bypass graft(s) of the extremities, other extremity |
| ICD10 | I70409 | Unspecified atherosclerosis of autologous vein bypass graft(s) of the extremities, unspecified extremity |
| ICD10 | I70411 | Atherosclerosis of autologous vein bypass graft(s) of the extremities with intermittent claudication, right leg |
| ICD10 | I70412 | Atherosclerosis of autologous vein bypass graft(s) of the extremities with intermittent claudication, left leg |
| ICD10 | I70413 | Atherosclerosis of autologous vein bypass graft(s) of the extremities with intermittent claudication, bilateral legs |
| ICD10 | I70418 | Atherosclerosis of autologous vein bypass graft(s) of the extremities with intermittent claudication, other extremity |
| ICD10 | I70419 | Atherosclerosis of autologous vein bypass graft(s) of the extremities with intermittent claudication, unspecified extremity |
| ICD10 | I70421 | Atherosclerosis of autologous vein bypass graft(s) of the extremities with rest pain, right leg |
| ICD10 | I70422 | Atherosclerosis of autologous vein bypass graft(s) of the extremities with rest pain, left leg |
| ICD10 | I70423 | Atherosclerosis of autologous vein bypass graft(s) of the extremities with rest pain, bilateral legs |
| ICD10 | I70428 | Atherosclerosis of autologous vein bypass graft(s) of the extremities with rest pain, other extremity |
| ICD10 | I70429 | Atherosclerosis of autologous vein bypass graft(s) of the extremities with rest pain, unspecified extremity |
| ICD10 | I70431 | Atherosclerosis of autologous vein bypass graft(s) of the right leg with ulceration of thigh |
| ICD10 | I70432 | Atherosclerosis of autologous vein bypass graft(s) of the right leg with ulceration of calf |
| ICD10 | I70433 | Atherosclerosis of autologous vein bypass graft(s) of the right leg with ulceration of ankle |
| ICD10 | I70434 | Atherosclerosis of autologous vein bypass graft(s) of the right leg with ulceration of heel and midfoot |
| ICD10 | I70435 | Atherosclerosis of autologous vein bypass graft(s) of the right leg with ulceration of other part of foot |
| ICD10 | I70438 | Atherosclerosis of autologous vein bypass graft(s) of the right leg with ulceration of other part of lower leg |
| ICD10 | I70439 | Atherosclerosis of autologous vein bypass graft(s) of the right leg with ulceration of unspecified site |
| ICD10 | I70441 | Atherosclerosis of autologous vein bypass graft(s) of the left leg with ulceration of thigh |
| ICD10 | I70442 | Atherosclerosis of autologous vein bypass graft(s) of the left leg with ulceration of calf |
| ICD10 | I70443 | Atherosclerosis of autologous vein bypass graft(s) of the left leg with ulceration of ankle |
| ICD10 | I70444 | Atherosclerosis of autologous vein bypass graft(s) of the left leg with ulceration of heel and midfoot |
| ICD10 | I70445 | Atherosclerosis of autologous vein bypass graft(s) of the left leg with ulceration of other part of foot |
| ICD10 | I70448 | Atherosclerosis of autologous vein bypass graft(s) of the left leg with ulceration of other part of lower leg |
| ICD10 | I70449 | Atherosclerosis of autologous vein bypass graft(s) of the left leg with ulceration of unspecified site |
| ICD10 | I7045 | Atherosclerosis of autologous vein bypass graft(s) of other extremity with ulceration |
| ICD10 | I70461 | Atherosclerosis of autologous vein bypass graft(s) of the extremities with gangrene, right leg |
| ICD10 | I70462 | Atherosclerosis of autologous vein bypass graft(s) of the extremities with gangrene, left leg |
| ICD10 | I70463 | Atherosclerosis of autologous vein bypass graft(s) of the extremities with gangrene, bilateral legs |
| ICD10 | I70468 | Atherosclerosis of autologous vein bypass graft(s) of the extremities with gangrene, other extremity |
| ICD10 | I70469 | Atherosclerosis of autologous vein bypass graft(s) of the extremities with gangrene, unspecified extremity |
| ICD10 | I70491 | Other atherosclerosis of autologous vein bypass graft(s) of the extremities, right leg |
| ICD10 | I70492 | Other atherosclerosis of autologous vein bypass graft(s) of the extremities, left leg |
| ICD10 | I70493 | Other atherosclerosis of autologous vein bypass graft(s) of the extremities, bilateral legs |
| ICD10 | I70498 | Other atherosclerosis of autologous vein bypass graft(s) of the extremities, other extremity |
| ICD10 | I70499 | Other atherosclerosis of autologous vein bypass graft(s) of the extremities, unspecified extremity |
| ICD10 | I70501 | Unspecified atherosclerosis of nonautologous biological bypass graft(s) of the extremities, right leg |
| ICD10 | I70502 | Unspecified atherosclerosis of nonautologous biological bypass graft(s) of the extremities, left leg |
| ICD10 | I70503 | Unspecified atherosclerosis of nonautologous biological bypass graft(s) of the extremities, bilateral legs |
| ICD10 | I70508 | Unspecified atherosclerosis of nonautologous biological bypass graft(s) of the extremities, other extremity |
| ICD10 | I70509 | Unspecified atherosclerosis of nonautologous biological bypass graft(s) of the extremities, unspecified extremity |
| ICD10 | I70511 | Atherosclerosis of nonautologous biological bypass graft(s) of the extremities with intermittent claudication, right leg |
| ICD10 | I70512 | Atherosclerosis of nonautologous biological bypass graft(s) of the extremities with intermittent claudication, left leg |
| ICD10 | I70513 | Atherosclerosis of nonautologous biological bypass graft(s) of the extremities with intermittent claudication, bilateral legs |
| ICD10 | I70518 | Atherosclerosis of nonautologous biological bypass graft(s) of the extremities with intermittent claudication, other extremity |
| ICD10 | I70519 | Atherosclerosis of nonautologous biological bypass graft(s) of the extremities with intermittent claudication, unspecified extremity |
| ICD10 | I70521 | Atherosclerosis of nonautologous biological bypass graft(s) of the extremities with rest pain, right leg |
| ICD10 | I70522 | Atherosclerosis of nonautologous biological bypass graft(s) of the extremities with rest pain, left leg |
| ICD10 | I70523 | Atherosclerosis of nonautologous biological bypass graft(s) of the extremities with rest pain, bilateral legs |
| ICD10 | I70528 | Atherosclerosis of nonautologous biological bypass graft(s) of the extremities with rest pain, other extremity |
| ICD10 | I70529 | Atherosclerosis of nonautologous biological bypass graft(s) of the extremities with rest pain, unspecified extremity |
| ICD10 | I70531 | Atherosclerosis of nonautologous biological bypass graft(s) of the right leg with ulceration of thigh |
| ICD10 | I70532 | Atherosclerosis of nonautologous biological bypass graft(s) of the right leg with ulceration of calf |
| ICD10 | I70533 | Atherosclerosis of nonautologous biological bypass graft(s) of the right leg with ulceration of ankle |
| ICD10 | I70534 | Atherosclerosis of nonautologous biological bypass graft(s) of the right leg with ulceration of heel and midfoot |
| ICD10 | I70535 | Atherosclerosis of nonautologous biological bypass graft(s) of the right leg with ulceration of other part of foot |
| ICD10 | I70538 | Atherosclerosis of nonautologous biological bypass graft(s) of the right leg with ulceration of other part of lower leg |
| ICD10 | I70539 | Atherosclerosis of nonautologous biological bypass graft(s) of the right leg with ulceration of unspecified site |
| ICD10 | I70541 | Atherosclerosis of nonautologous biological bypass graft(s) of the left leg with ulceration of thigh |
| ICD10 | I70542 | Atherosclerosis of nonautologous biological bypass graft(s) of the left leg with ulceration of calf |
| ICD10 | I70543 | Atherosclerosis of nonautologous biological bypass graft(s) of the left leg with ulceration of ankle |
| ICD10 | I70544 | Atherosclerosis of nonautologous biological bypass graft(s) of the left leg with ulceration of heel and midfoot |
| ICD10 | I70545 | Atherosclerosis of nonautologous biological bypass graft(s) of the left leg with ulceration of other part of foot |
| ICD10 | I70548 | Atherosclerosis of nonautologous biological bypass graft(s) of the left leg with ulceration of other part of lower leg |
| ICD10 | I70549 | Atherosclerosis of nonautologous biological bypass graft(s) of the left leg with ulceration of unspecified site |
| ICD10 | I7055 | Atherosclerosis of nonautologous biological bypass graft(s) of other extremity with ulceration |
| ICD10 | I70561 | Atherosclerosis of nonautologous biological bypass graft(s) of the extremities with gangrene, right leg |
| ICD10 | I70562 | Atherosclerosis of nonautologous biological bypass graft(s) of the extremities with gangrene, left leg |
| ICD10 | I70563 | Atherosclerosis of nonautologous biological bypass graft(s) of the extremities with gangrene, bilateral legs |
| ICD10 | I70568 | Atherosclerosis of nonautologous biological bypass graft(s) of the extremities with gangrene, other extremity |
| ICD10 | I70569 | Atherosclerosis of nonautologous biological bypass graft(s) of the extremities with gangrene, unspecified extremity |
| ICD10 | I70591 | Other atherosclerosis of nonautologous biological bypass graft(s) of the extremities, right leg |
| ICD10 | I70592 | Other atherosclerosis of nonautologous biological bypass graft(s) of the extremities, left leg |
| ICD10 | I70593 | Other atherosclerosis of nonautologous biological bypass graft(s) of the extremities, bilateral legs |
| ICD10 | I70598 | Other atherosclerosis of nonautologous biological bypass graft(s) of the extremities, other extremity |
| ICD10 | I70599 | Other atherosclerosis of nonautologous biological bypass graft(s) of the extremities, unspecified extremity |
| ICD10 | I70601 | Unspecified atherosclerosis of nonbiological bypass graft(s) of the extremities, right leg |
| ICD10 | I70602 | Unspecified atherosclerosis of nonbiological bypass graft(s) of the extremities, left leg |
| ICD10 | I70603 | Unspecified atherosclerosis of nonbiological bypass graft(s) of the extremities, bilateral legs |
| ICD10 | I70608 | Unspecified atherosclerosis of nonbiological bypass graft(s) of the extremities, other extremity |
| ICD10 | I70609 | Unspecified atherosclerosis of nonbiological bypass graft(s) of the extremities, unspecified extremity |
| ICD10 | I70611 | Atherosclerosis of nonbiological bypass graft(s) of the extremities with intermittent claudication, right leg |
| ICD10 | I70612 | Atherosclerosis of nonbiological bypass graft(s) of the extremities with intermittent claudication, left leg |
| ICD10 | I70613 | Atherosclerosis of nonbiological bypass graft(s) of the extremities with intermittent claudication, bilateral legs |
| ICD10 | I70618 | Atherosclerosis of nonbiological bypass graft(s) of the extremities with intermittent claudication, other extremity |
| ICD10 | I70619 | Atherosclerosis of nonbiological bypass graft(s) of the extremities with intermittent claudication, unspecified extremity |
| ICD10 | I70621 | Atherosclerosis of nonbiological bypass graft(s) of the extremities with rest pain, right leg |
| ICD10 | I70622 | Atherosclerosis of nonbiological bypass graft(s) of the extremities with rest pain, left leg |
| ICD10 | I70623 | Atherosclerosis of nonbiological bypass graft(s) of the extremities with rest pain, bilateral legs |
| ICD10 | I70628 | Atherosclerosis of nonbiological bypass graft(s) of the extremities with rest pain, other extremity |
| ICD10 | I70629 | Atherosclerosis of nonbiological bypass graft(s) of the extremities with rest pain, unspecified extremity |
| ICD10 | I70631 | Atherosclerosis of nonbiological bypass graft(s) of the right leg with ulceration of thigh |
| ICD10 | I70632 | Atherosclerosis of nonbiological bypass graft(s) of the right leg with ulceration of calf |
| ICD10 | I70633 | Atherosclerosis of nonbiological bypass graft(s) of the right leg with ulceration of ankle |
| ICD10 | I70634 | Atherosclerosis of nonbiological bypass graft(s) of the right leg with ulceration of heel and midfoot |
| ICD10 | I70635 | Atherosclerosis of nonbiological bypass graft(s) of the right leg with ulceration of other part of foot |
| ICD10 | I70638 | Atherosclerosis of nonbiological bypass graft(s) of the right leg with ulceration of other part of lower leg |
| ICD10 | I70639 | Atherosclerosis of nonbiological bypass graft(s) of the right leg with ulceration of unspecified site |
| ICD10 | I70641 | Atherosclerosis of nonbiological bypass graft(s) of the left leg with ulceration of thigh |
| ICD10 | I70642 | Atherosclerosis of nonbiological bypass graft(s) of the left leg with ulceration of calf |
| ICD10 | I70643 | Atherosclerosis of nonbiological bypass graft(s) of the left leg with ulceration of ankle |
| ICD10 | I70644 | Atherosclerosis of nonbiological bypass graft(s) of the left leg with ulceration of heel and midfoot |
| ICD10 | I70645 | Atherosclerosis of nonbiological bypass graft(s) of the left leg with ulceration of other part of foot |
| ICD10 | I70648 | Atherosclerosis of nonbiological bypass graft(s) of the left leg with ulceration of other part of lower leg |
| ICD10 | I70649 | Atherosclerosis of nonbiological bypass graft(s) of the left leg with ulceration of unspecified site |
| ICD10 | I7065 | Atherosclerosis of nonbiological bypass grafts of other extremity with ulceration |
| ICD10 | I70661 | Atherosclerosis of nonbiological bypass graft(s) of the extremities with gangrene, right leg |
| ICD10 | I70662 | Atherosclerosis of nonbiological bypass graft(s) of the extremities with gangrene, left leg |
| ICD10 | I70663 | Atherosclerosis of nonbiological bypass graft(s) of the extremities with gangrene, bilateral legs |
| ICD10 | I70668 | Atherosclerosis of nonbiological bypass graft(s) of the extremities with gangrene, other extremity |
| ICD10 | I70669 | Atherosclerosis of nonbiological bypass graft(s) of the extremities with gangrene, unspecified extremity |
| ICD10 | I70691 | Other atherosclerosis of nonbiological bypass graft(s) of the extremities, right leg |
| ICD10 | I70692 | Other atherosclerosis of nonbiological bypass graft(s) of the extremities, left leg |
| ICD10 | I70693 | Other atherosclerosis of nonbiological bypass graft(s) of the extremities, bilateral legs |
| ICD10 | I70698 | Other atherosclerosis of nonbiological bypass graft(s) of the extremities, other extremity |
| ICD10 | I70699 | Other atherosclerosis of nonbiological bypass graft(s) of the extremities, unspecified extremity |
| ICD10 | I70701 | Unspecified atherosclerosis of other type of bypass graft(s) of the extremities, right leg |
| ICD10 | I70702 | Unspecified atherosclerosis of other type of bypass graft(s) of the extremities, left leg |
| ICD10 | I70703 | Unspecified atherosclerosis of other type of bypass graft(s) of the extremities, bilateral legs |
| ICD10 | I70708 | Unspecified atherosclerosis of other type of bypass graft(s) of the extremities, other extremity |
| ICD10 | I70709 | Unspecified atherosclerosis of other type of bypass graft(s) of the extremities, unspecified extremity |
| ICD10 | I70711 | Atherosclerosis of other type of bypass graft(s) of the extremities with intermittent claudication, right leg |
| ICD10 | I70712 | Atherosclerosis of other type of bypass graft(s) of the extremities with intermittent claudication, left leg |
| ICD10 | I70713 | Atherosclerosis of other type of bypass graft(s) of the extremities with intermittent claudication, bilateral legs |
| ICD10 | I70718 | Atherosclerosis of other type of bypass graft(s) of the extremities with intermittent claudication, other extremity |
| ICD10 | I70719 | Atherosclerosis of other type of bypass graft(s) of the extremities with intermittent claudication, unspecified extremity |
| ICD10 | I70721 | Atherosclerosis of other type of bypass graft(s) of the extremities with rest pain, right leg |
| ICD10 | I70722 | Atherosclerosis of other type of bypass graft(s) of the extremities with rest pain, left leg |
| ICD10 | I70723 | Atherosclerosis of other type of bypass graft(s) of the extremities with rest pain, bilateral legs |
| ICD10 | I70728 | Atherosclerosis of other type of bypass graft(s) of the extremities with rest pain, other extremity |
| ICD10 | I70729 | Atherosclerosis of other type of bypass graft(s) of the extremities with rest pain, unspecified extremity |
| ICD10 | I70731 | Atherosclerosis of other type of bypass graft(s) of the right leg with ulceration of thigh |
| ICD10 | I70732 | Atherosclerosis of other type of bypass graft(s) of the right leg with ulceration of calf |
| ICD10 | I70733 | Atherosclerosis of other type of bypass graft(s) of the right leg with ulceration of ankle |
| ICD10 | I70734 | Atherosclerosis of other type of bypass graft(s) of the right leg with ulceration of heel and midfoot |
| ICD10 | I70735 | Atherosclerosis of other type of bypass graft(s) of the right leg with ulceration of other part of foot |
| ICD10 | I70738 | Atherosclerosis of other type of bypass graft(s) of the right leg with ulceration of other part of lower leg |
| ICD10 | I70739 | Atherosclerosis of other type of bypass graft(s) of the right leg with ulceration of unspecified site |
| ICD10 | I70741 | Atherosclerosis of other type of bypass graft(s) of the left leg with ulceration of thigh |
| ICD10 | I70742 | Atherosclerosis of other type of bypass graft(s) of the left leg with ulceration of calf |
| ICD10 | I70743 | Atherosclerosis of other type of bypass graft(s) of the left leg with ulceration of ankle |
| ICD10 | I70744 | Atherosclerosis of other type of bypass graft(s) of the left leg with ulceration of heel and midfoot |
| ICD10 | I70745 | Atherosclerosis of other type of bypass graft(s) of the left leg with ulceration of other part of foot |
| ICD10 | I70748 | Atherosclerosis of other type of bypass graft(s) of the left leg with ulceration of other part of lower leg |
| ICD10 | I70749 | Atherosclerosis of other type of bypass graft(s) of the left leg with ulceration of unspecified site |
| ICD10 | I7075 | Atherosclerosis of other type of bypass graft(s) of other extremity with ulceration |
| ICD10 | I70761 | Atherosclerosis of other type of bypass graft(s) of the extremities with gangrene, right leg |
| ICD10 | I70762 | Atherosclerosis of other type of bypass graft(s) of the extremities with gangrene, left leg |
| ICD10 | I70763 | Atherosclerosis of other type of bypass graft(s) of the extremities with gangrene, bilateral legs |
| ICD10 | I70768 | Atherosclerosis of other type of bypass graft(s) of the extremities with gangrene, other extremity |
| ICD10 | I70769 | Atherosclerosis of other type of bypass graft(s) of the extremities with gangrene, unspecified extremity |
| ICD10 | I70791 | Other atherosclerosis of other type of bypass graft(s) of the extremities, right leg |
| ICD10 | I70792 | Other atherosclerosis of other type of bypass graft(s) of the extremities, left leg |
| ICD10 | I70793 | Other atherosclerosis of other type of bypass graft(s) of the extremities, bilateral legs |
| ICD10 | I70798 | Other atherosclerosis of other type of bypass graft(s) of the extremities, other extremity |
| ICD10 | I70799 | Other atherosclerosis of other type of bypass graft(s) of the extremities, unspecified extremity |
| ICD10 | I7090 | Unspecified atherosclerosis |
| ICD10 | I7091 | Generalized atherosclerosis |
| ICD10 | I7092 | Chronic total occlusion of artery of the extremities |
| ICD10 | I739 | Peripheral vascular disease, unspecified |
| ICD10 | I7401 | Saddle embolus of abdominal aorta |
| ICD10 | I7409 | Other arterial embolism and thrombosis of abdominal aorta |
| ICD10 | I7410 | Embolism and thrombosis of unspecified parts of aorta |
| ICD10 | I7419 | Embolism and thrombosis of other parts of aorta |
| ICD10 | I743 | Embolism and thrombosis of arteries of the lower extremities |
| ICD10 | I744 | Embolism and thrombosis of arteries of extremities, unspecified |
| ICD10 | I745 | Embolism and thrombosis of iliac artery |
| ICD10 | I748 | Embolism and thrombosis of other arteries |
| ICD10 | I75021 | Atheroembolism of right lower extremity |
| ICD10 | I75022 | Atheroembolism of left lower extremity |
| ICD10 | I75023 | Atheroembolism of bilateral lower extremities |
| ICD10 | I75029 | Atheroembolism of unspecified lower extremity |
| ICD10 | I771 | Stricture of artery |
| ICD10 | I96 | Gangrene, not elsewhere classified |
| ICD10 | L97101 | Non-pressure chronic ulcer of unspecified thigh limited to breakdown of skin |
| ICD10 | L97102 | Non-pressure chronic ulcer of unspecified thigh with fat layer exposed |
| ICD10 | L97103 | Non-pressure chronic ulcer of unspecified thigh with necrosis of muscle |
| ICD10 | L97104 | Non-pressure chronic ulcer of unspecified thigh with necrosis of bone |
| ICD10 | L97109 | Non-pressure chronic ulcer of unspecified thigh with unspecified severity |
| ICD10 | L97111 | Non-pressure chronic ulcer of right thigh limited to breakdown of skin |
| ICD10 | L97112 | Non-pressure chronic ulcer of right thigh with fat layer exposed |
| ICD10 | L97113 | Non-pressure chronic ulcer of right thigh with necrosis of muscle |
| ICD10 | L97114 | Non-pressure chronic ulcer of right thigh with necrosis of bone |
| ICD10 | L97119 | Non-pressure chronic ulcer of right thigh with unspecified severity |
| ICD10 | L97121 | Non-pressure chronic ulcer of left thigh limited to breakdown of skin |
| ICD10 | L97122 | Non-pressure chronic ulcer of left thigh with fat layer exposed |
| ICD10 | L97123 | Non-pressure chronic ulcer of left thigh with necrosis of muscle |
| ICD10 | L97124 | Non-pressure chronic ulcer of left thigh with necrosis of bone |
| ICD10 | L97129 | Non-pressure chronic ulcer of left thigh with unspecified severity |
| ICD10 | L97201 | Non-pressure chronic ulcer of unspecified calf limited to breakdown of skin |
| ICD10 | L97202 | Non-pressure chronic ulcer of unspecified calf with fat layer exposed |
| ICD10 | L97203 | Non-pressure chronic ulcer of unspecified calf with necrosis of muscle |
| ICD10 | L97204 | Non-pressure chronic ulcer of unspecified calf with necrosis of bone |
| ICD10 | L97209 | Non-pressure chronic ulcer of unspecified calf with unspecified severity |
| ICD10 | L97211 | Non-pressure chronic ulcer of right calf limited to breakdown of skin |
| ICD10 | L97212 | Non-pressure chronic ulcer of right calf with fat layer exposed |
| ICD10 | L97213 | Non-pressure chronic ulcer of right calf with necrosis of muscle |
| ICD10 | L97214 | Non-pressure chronic ulcer of right calf with necrosis of bone |
| ICD10 | L97219 | Non-pressure chronic ulcer of right calf with unspecified severity |
| ICD10 | L97221 | Non-pressure chronic ulcer of left calf limited to breakdown of skin |
| ICD10 | L97222 | Non-pressure chronic ulcer of left calf with fat layer exposed |
| ICD10 | L97223 | Non-pressure chronic ulcer of left calf with necrosis of muscle |
| ICD10 | L97224 | Non-pressure chronic ulcer of left calf with necrosis of bone |
| ICD10 | L97229 | Non-pressure chronic ulcer of left calf with unspecified severity |
| ICD10 | L97301 | Non-pressure chronic ulcer of unspecified ankle limited to breakdown of skin |
| ICD10 | L97302 | Non-pressure chronic ulcer of unspecified ankle with fat layer exposed |
| ICD10 | L97303 | Non-pressure chronic ulcer of unspecified ankle with necrosis of muscle |
| ICD10 | L97304 | Non-pressure chronic ulcer of unspecified ankle with necrosis of bone |
| ICD10 | L97309 | Non-pressure chronic ulcer of unspecified ankle with unspecified severity |
| ICD10 | L97311 | Non-pressure chronic ulcer of right ankle limited to breakdown of skin |
| ICD10 | L97312 | Non-pressure chronic ulcer of right ankle with fat layer exposed |
| ICD10 | L97313 | Non-pressure chronic ulcer of right ankle with necrosis of muscle |
| ICD10 | L97314 | Non-pressure chronic ulcer of right ankle with necrosis of bone |
| ICD10 | L97319 | Non-pressure chronic ulcer of right ankle with unspecified severity |
| ICD10 | L97321 | Non-pressure chronic ulcer of left ankle limited to breakdown of skin |
| ICD10 | L97322 | Non-pressure chronic ulcer of left ankle with fat layer exposed |
| ICD10 | L97323 | Non-pressure chronic ulcer of left ankle with necrosis of muscle |
| ICD10 | L97324 | Non-pressure chronic ulcer of left ankle with necrosis of bone |
| ICD10 | L97329 | Non-pressure chronic ulcer of left ankle with unspecified severity |
| ICD10 | L97401 | Non-pressure chronic ulcer of unspecified heel and midfoot limited to breakdown of skin |
| ICD10 | L97402 | Non-pressure chronic ulcer of unspecified heel and midfoot with fat layer exposed |
| ICD10 | L97403 | Non-pressure chronic ulcer of unspecified heel and midfoot with necrosis of muscle |
| ICD10 | L97404 | Non-pressure chronic ulcer of unspecified heel and midfoot with necrosis of bone |
| ICD10 | L97409 | Non-pressure chronic ulcer of unspecified heel and midfoot with unspecified severity |
| ICD10 | L97411 | Non-pressure chronic ulcer of right heel and midfoot limited to breakdown of skin |
| ICD10 | L97412 | Non-pressure chronic ulcer of right heel and midfoot with fat layer exposed |
| ICD10 | L97413 | Non-pressure chronic ulcer of right heel and midfoot with necrosis of muscle |
| ICD10 | L97414 | Non-pressure chronic ulcer of right heel and midfoot with necrosis of bone |
| ICD10 | L97419 | Non-pressure chronic ulcer of right heel and midfoot with unspecified severity |
| ICD10 | L97421 | Non-pressure chronic ulcer of left heel and midfoot limited to breakdown of skin |
| ICD10 | L97422 | Non-pressure chronic ulcer of left heel and midfoot with fat layer exposed |
| ICD10 | L97423 | Non-pressure chronic ulcer of left heel and midfoot with necrosis of muscle |
| ICD10 | L97424 | Non-pressure chronic ulcer of left heel and midfoot with necrosis of bone |
| ICD10 | L97429 | Non-pressure chronic ulcer of left heel and midfoot with unspecified severity |
| ICD10 | L97501 | Non-pressure chronic ulcer of other part of unspecified foot limited to breakdown of skin |
| ICD10 | L97502 | Non-pressure chronic ulcer of other part of unspecified foot with fat layer exposed |
| ICD10 | L97503 | Non-pressure chronic ulcer of other part of unspecified foot with necrosis of muscle |
| ICD10 | L97504 | Non-pressure chronic ulcer of other part of unspecified foot with necrosis of bone |
| ICD10 | L97509 | Non-pressure chronic ulcer of other part of unspecified foot with unspecified severity |
| ICD10 | L97511 | Non-pressure chronic ulcer of other part of right foot limited to breakdown of skin |
| ICD10 | L97512 | Non-pressure chronic ulcer of other part of right foot with fat layer exposed |
| ICD10 | L97513 | Non-pressure chronic ulcer of other part of right foot with necrosis of muscle |
| ICD10 | L97514 | Non-pressure chronic ulcer of other part of right foot with necrosis of bone |
| ICD10 | L97519 | Non-pressure chronic ulcer of other part of right foot with unspecified severity |
| ICD10 | L97521 | Non-pressure chronic ulcer of other part of left foot limited to breakdown of skin |
| ICD10 | L97522 | Non-pressure chronic ulcer of other part of left foot with fat layer exposed |
| ICD10 | L97523 | Non-pressure chronic ulcer of other part of left foot with necrosis of muscle |
| ICD10 | L97524 | Non-pressure chronic ulcer of other part of left foot with necrosis of bone |
| ICD10 | L97529 | Non-pressure chronic ulcer of other part of left foot with unspecified severity |
| ICD10 | L97801 | Non-pressure chronic ulcer of other part of unspecified lower leg limited to breakdown of skin |
| ICD10 | L97802 | Non-pressure chronic ulcer of other part of unspecified lower leg with fat layer exposed |
| ICD10 | L97803 | Non-pressure chronic ulcer of other part of unspecified lower leg with necrosis of muscle |
| ICD10 | L97804 | Non-pressure chronic ulcer of other part of unspecified lower leg with necrosis of bone |
| ICD10 | L97809 | Non-pressure chronic ulcer of other part of unspecified lower leg with unspecified severity |
| ICD10 | L97811 | Non-pressure chronic ulcer of other part of right lower leg limited to breakdown of skin |
| ICD10 | L97812 | Non-pressure chronic ulcer of other part of right lower leg with fat layer exposed |
| ICD10 | L97813 | Non-pressure chronic ulcer of other part of right lower leg with necrosis of muscle |
| ICD10 | L97814 | Non-pressure chronic ulcer of other part of right lower leg with necrosis of bone |
| ICD10 | L97819 | Non-pressure chronic ulcer of other part of right lower leg with unspecified severity |
| ICD10 | L97821 | Non-pressure chronic ulcer of other part of left lower leg limited to breakdown of skin |
| ICD10 | L97822 | Non-pressure chronic ulcer of other part of left lower leg with fat layer exposed |
| ICD10 | L97823 | Non-pressure chronic ulcer of other part of left lower leg with necrosis of muscle |
| ICD10 | L97824 | Non-pressure chronic ulcer of other part of left lower leg with necrosis of bone |
| ICD10 | L97829 | Non-pressure chronic ulcer of other part of left lower leg with unspecified severity |
| ICD10 | L97901 | Non-pressure chronic ulcer of unspecified part of unspecified lower leg limited to breakdown of skin |
| ICD10 | L97902 | Non-pressure chronic ulcer of unspecified part of unspecified lower leg with fat layer exposed |
| ICD10 | L97903 | Non-pressure chronic ulcer of unspecified part of unspecified lower leg with necrosis of muscle |
| ICD10 | L97904 | Non-pressure chronic ulcer of unspecified part of unspecified lower leg with necrosis of bone |
| ICD10 | L97909 | Non-pressure chronic ulcer of unspecified part of unspecified lower leg with unspecified severity |
| ICD10 | L97911 | Non-pressure chronic ulcer of unspecified part of right lower leg limited to breakdown of skin |
| ICD10 | L97912 | Non-pressure chronic ulcer of unspecified part of right lower leg with fat layer exposed |
| ICD10 | L97913 | Non-pressure chronic ulcer of unspecified part of right lower leg with necrosis of muscle |
| ICD10 | L97914 | Non-pressure chronic ulcer of unspecified part of right lower leg with necrosis of bone |
| ICD10 | L97919 | Non-pressure chronic ulcer of unspecified part of right lower leg with unspecified severity |
| ICD10 | L97921 | Non-pressure chronic ulcer of unspecified part of left lower leg limited to breakdown of skin |
| ICD10 | L97922 | Non-pressure chronic ulcer of unspecified part of left lower leg with fat layer exposed |
| ICD10 | L97923 | Non-pressure chronic ulcer of unspecified part of left lower leg with necrosis of muscle |
| ICD10 | L97924 | Non-pressure chronic ulcer of unspecified part of left lower leg with necrosis of bone |
| ICD10 | L97929 | Non-pressure chronic ulcer of unspecified part of left lower leg with unspecified severity |
