# Supplementary material for: Model-Based Algorithms for Detecting Peripheral Artery Disease Using Administrative Data From an Electronic Health Record Data System: Algorithm Development Study
Source: JMIR Med Inform. 2020 Aug 19;8(8):e18542. doi: 10.2196/18542 (PMC7468640; doi:10.2196/18542)
Supplement: Multimedia Appendix 2 [file medinform_v8i8e18542_app2.docx]

**Appendix 2:**

CPT procedure codes (revascularization and imaging)

| **Code** | **Definition** |
| --- | --- |
| 0238T | Catheter removal of plaque from groin artery, accessed through the skin or open procedure including radiological supervision and interpretation |
| 35381 | Thromboendarterectomy, with or without patch graft; femoral and/or popliteal, and/or tibioperoneal |
| 35450 | Balloon dilation of narrowed or blocked kidney or abdominal organ artery, open procedure |
| 35452 | Balloon dilation of narrowed or blocked aorta, open procedure |
| 35454 | Transluminal balloon angioplasty, open; iliac |
| 35456 | Transluminal balloon angioplasty, open; femoral-popliteal |
| 35459 | Transluminal balloon angioplasty, open; tibioperoneal trunk and branches |
| 35470 | Transluminal balloon angioplasty, percutaneous; tibioperoneal trunk or branches, each vessel |
| 35472 | Balloon dilation of narrowed or blocked aorta, accessed through the skin |
| 35473 | Transluminal balloon angioplasty, percutaneous; iliac |
| 35474 | Transluminal balloon angioplasty, percutaneous; femoral-popliteal |
| 35481 | Transluminal peripheral atherectomy, open; aortic |
| 35482 | Transluminal peripheral atherectomy, open; iliac |
| 35483 | Transluminal peripheral atherectomy, open; femoral-popliteal |
| 35485 | Transluminal peripheral atherectomy, open; tibioperoneal trunk and branches |
| 35491 | Transluminal peripheral atherectomy, percutaneous; aortic |
| 35492 | Transluminal peripheral atherectomy, percutaneous; iliac |
| 35493 | Transluminal peripheral atherectomy, percutaneous; femoral-popliteal |
| 35495 | Transluminal peripheral atherectomy, percutaneous; tibioperoneal trunk and branches |
| 35521 | Bypass of diseased or blocked artery (arm to upper leg artery) |
| 35533 | Bypass of diseased or blocked artery (arm to upper leg artery) |
| 35537 | Bypass of diseased or blocked artery (aorta to groin artery) |
| 35538 | Bypass of diseased or blocked artery (aorta to groin and opposite groin artery) |
| 35539 | Bypass of diseased or blocked artery (aorta to upper leg artery) |
| 35540 | Bypass of diseased or blocked artery (aorta to upper leg and opposite upper leg artery) |
| 35541 | Bypass graft, with vein; aortoiliac or bi-iliac bypass graft |
| 35546 | Bypass graft, with vein; Aortofemoral or bifemoral |
| 35548 | Bypass graft, with vein; aortoiliofemoral, unilateral |
| 35549 | Bypass graft, with vein; aortoiliofemoral, bilateral |
| 35551 | Bypass graft, with vein; aortofemoral-popliteal |
| 35556 | Bypass of diseased or blocked artery (upper to lower leg artery) |
| 35558 | Bypass of diseased or blocked artery (upper leg to opposite upper leg artery) |
| 35563 | Bypass of diseased or blocked artery (groin to opposite groin artery) |
| 35565 | Bypass of diseased or blocked artery (groin to upper leg artery) |
| 35566 | Bypass of diseased or blocked artery (upper leg to lower leg artery) |
| 35571 | Bypass of diseased or blocked artery (lower leg to lower leg artery) |
| 35582 | In-situ vein bypass; aortofemoral-popliteal (only femoral-popliteal portion in-situ) |
| 35583 | Bypass of diseased or blocked artery (upper leg to lower thigh artery) |
| 35585 | Bypass of diseased or blocked artery (upper leg to lower leg artery) |
| 35587 | Bypass of diseased or blocked artery (lower thigh to lower leg artery) |
| 35621 | Bypass of diseased or blocked artery (arm to chest artery) |
| 35623 | Bypass of diseased or blocked artery (arm to lower leg artery) |
| 35637 | Bypass of diseased or blocked artery (aorta to groin artery) |
| 35638 | Bypass of diseased or blocked artery (aorta to groin to opposite groin artery) |
| 35641 | Bypass graft, with other than vein; aortoiliac or bi-iliac |
| 35646 | Bypass of diseased or blocked artery (aorta to upper leg and opposite upper leg artery) |
| 35647 | Bypass of diseased or blocked artery (aorta to upper leg artery) |
| 35651 | Bypass graft, with other than vein; aortofemoral-popliteal |
| 35654 | Bypass of diseased or blocked artery (arm to both lower thigh arteries) |
| 35656 | Bypass of diseased or blocked artery (upper leg to lower thigh artery) |
| 35661 | Bypass of diseased or blocked artery (upper leg to opposite upper leg artery) |
| 35663 | Bypass of diseased or blocked artery (groin to opposite groin artery) |
| 35665 | Bypass on diseased or blocked groin to upper leg artery |
| 35666 | Bypass of diseased or blocked artery (upper leg to lower leg arteries) |
| 35671 | Bypass of diseased or blocked artery (upper leg to lower leg arteries) |
| 35879 | Revision of arterial bypass of leg with placement of vein patch, open procedure |
| 35881 | Revision of arterial bypass of leg with placement of relocated vein, open procedure |
| 35883 | Revision of arterial bypass of groin with placement synthetic graft, open procedure |
| 35884 | Revision of arterial bypass of groin with vein patch graft, open procedure |
| 36140 | Insertion of needle or catheter into an artery of arm or leg |
| 36200 | Insertion of catheter into aorta |
| 36245 | Insertion of catheter into abdominal pelvic or leg artery |
| 36246 | Insertion of catheter into abdominal pelvic or leg artery |
| 36247 | Insertion of catheter into abdominal pelvic or leg artery |
| 36248 | Insertion of catheter into each additional abdominal, pelvic or leg artery |
| 37184 | Removal of blood clot and injections to dissolve blood clot from artery or arterial graft using fluoroscopic guidance, accessed through the skin |
| 37185 | Removal of blood clot and injections to dissolve blood clot from artery or arterial graft using fluoroscopic guidance, accessed through the skin |
| 37186 | Removal of blood clot and injections to dissolve blood clot from artery or arterial graft using fluoroscopic guidance, accessed beneath the skin |
| 37205 | Insertion of stent into blood vessel |
| 37206 | Insertion of stent into blood vessel |
| 37207 | Insertion of stent in blood vessel via catheter |
| 37208 | Insertion of stent in blood vessel via catheter |
| 37220 | Balloon dilation of artery in one side of groin, endovascular, accessed through the skin or open procedure |
| 37221 | Insertion of stents in artery in one side of groin, endovascular, accessed through the skin or open procedure |
| 37222 | Balloon dilation of groin artery, endovascular, open, or percutaneous approach |
| 37223 | Insertion of stents into groin artery, endovascular, accessed through the skin or open procedure |
| 37224 | Balloon dilation of arteries in one leg, endovascular, accessed through the skin or open procedure |
| 37225 | Removal of plaque in arteries in one leg, endovascular, accessed through the skin or open procedure |
| 37226 | Insertion of stents into arteries in one leg, endovascular, accessed through the skin or open procedure |
| 37227 | Removal of plaque and insertion of stents into arteries in one leg, endovascular, accessed through the skin or open procedure |
| 37228 | Balloon dilation of artery of one leg, endovascular, accessed through the skin or open procedure |
| 37229 | Removal of plaque in artery in one leg, endovascular, accessed through the skin or open procedure |
| 37230 | Insertion of stents into artery in one leg, endovascular, accessed through the skin or open procedure |
| 37231 | Removal of plaque and insertion of stents into artery in one leg, endovascular, accessed through the skin or open procedure |
| 37232 | Balloon dilation of artery in one leg, endovascular, accessed through the skin or open procedure |
| 37233 | Removal of plaque in artery in one leg, endovascular, accessed through the skin or open procedure |
| 37234 | Insertion of stents into artery in one leg, endovascular, accessed through the skin or open procedure |
| 37235 | Removal of plaque and insertion of stents into artery in one leg, endovascular, accessed through the skin or open procedure |
| 37236 | Insertion of intravascular stents in artery (except lower extremity, cervical carotid, extracranial vertebral or intrathoracic carotid, intracranial, or coronary), open or accessed through the skin, with radiological supervision and interpretation |
| 37237 | Insertion of intravascular stents in artery (except lower extremity, cervical carotid, extracranial vertebral or intrathoracic carotid, intracranial, or coronary), open or accessed through the skin, with radiological supervision and interpretation |
| 37238 | Insertion of intravascular stents in vein, open or accessed through the skin, with radiological supervision and interpretation |
| 37239 | Insertion of intravascular stents in vein, open or accessed through the skin, with radiological supervision and interpretation |
| 72191 | CT scan of pelvic blood vessels with contrast |
| 72198 | MRA scan of pelvic blood vessels |
| 73706 | CT scan of lower leg blood vessels with contrast |
| 73725 | MRA scan of leg blood vessels |
| 74185 | MRI scan of blood vessels of abdomen |
| 75630 | Radiological supervision and interpretation X-ray of abdominal aorta and both leg arteries |
| 75635 | CT scan of abdominal aorta and both leg arteries with contrast |
| 75710 | Radiological supervision and interpretation of imaging of artery of one arm or leg |
| 75716 | Radiological supervision and interpretation of imaging of arteries of both arms or legs |
| 76350 | Subtraction in conjunction with contrast studies |
| 93922 | Ultrasound study of arteries of both arms and legs |
| 93923 | Ultrasound study of arteries of both arms and legs |
| 93924 | Ultrasound study of arteries of both legs at rest and exercise |
| 93925 | Ultrasound study of arteries and arterial grafts of both legs |
| 93926 | Ultrasound study of arteries and arterial grafts of one leg or limited |
| 93978 | Ultrasound scan of vena cava or groin graft or vessel blood flow |
| 93979 | Ultrasound scan of blood flow of aorta, vena cava, bypass graphs, or one side of the groin or limited scan |

ICD-9 procedure codes

| **Code** | **Definition** |
| --- | --- |
| 00.40 | Procedure on single vessel |
| 00.41 | Procedure on two vessels |
| 00.42 | Procedure on three vessels |
| 00.43 | Procedure on four or more vessels |
| 00.44 | Procedure on vessel bifurcation |
| 00.45 | Insertion of one vascular stent |
| 00.46 | Insertion of two vascular stents |
| 00.47 | Insertion of three vascular stents |
| 00.48 | Insertion of four or more vascular stents |
| 00.55 | Insertion of drug-eluting stent(s) of other peripheral vessel(s) |
| 00.60 | Insertion of drug-eluting stent(s) of superficial femoral artery |
| 17.56 | Atherectomy of other non-coronary vessel(s) |
| 38.08 | Incision of vessel, lower limb arteries |
| 38.18 | Endarterectomy, lower limb arteries |
| 38.38 | Resection of vessel with anastomosis, lower limb arteries |
| 38.48 | Resection of vessel with replacement, lower limb arteries |
| 39.25 | Aorta-iliac-femoral bypass |
| 39.29 | Other (peripheral) vascular shunt or bypass |
| 39.49 | Other revision of vascular procedure |
| 39.50 | Angioplasty of other non-coronary vessel(s) |
| 39.56 | Repair of blood vessel with tissue patch graft |
| 39.57 | Repair of blood vessel with synthetic patch graft |
| 39.58 | Repair of blood vessel with unspecified type of patch graft |
| 39.90 | Insertion of non-drug-eluting peripheral (non-coronary) vessel stent(s) |
| 88.48 | Arteriography of femoral and other lower extremity arteries |

**ICD-10 procedure codes**

| **Code** | **Definition** |
| --- | --- |
| 047C04Z | Dilation of Right Common Iliac Artery with Drug-eluting Intraluminal Device, Open Approach |
| 047C0DZ | Dilation of Right Common Iliac Artery with Intraluminal Device, Open Approach |
| 047C0ZZ | Dilation of Right Common Iliac Artery, Open Approach |
| 047C34Z | Dilation of Right Common Iliac Artery with Drug-eluting Intraluminal Device, Percutaneous Approach |
| 047C3DZ | Dilation of Right Common Iliac Artery with Intraluminal Device, Percutaneous Approach |
| 047C3ZZ | Dilation of Right Common Iliac Artery, Percutaneous Approach |
| 047C44Z | Dilation of Right Common Iliac Artery with Drug-eluting Intraluminal Device, Percutaneous Endoscopic Approach |
| 047C4DZ | Dilation of Right Common Iliac Artery with Intraluminal Device, Percutaneous Endoscopic Approach |
| 047C4ZZ | Dilation of Right Common Iliac Artery, Percutaneous Endoscopic Approach |
| 047D04Z | Dilation of Left Common Iliac Artery with Drug-eluting Intraluminal Device, Open Approach |
| 047D0DZ | Dilation of Left Common Iliac Artery with Intraluminal Device, Open Approach |
| 047D0ZZ | Dilation of Left Common Iliac Artery, Open Approach |
| 047D34Z | Dilation of Left Common Iliac Artery with Drug-eluting Intraluminal Device, Percutaneous Approach |
| 047D3DZ | Dilation of Left Common Iliac Artery with Intraluminal Device, Percutaneous Approach |
| 047D3ZZ | Dilation of Left Common Iliac Artery, Percutaneous Approach |
| 047D44Z | Dilation of Left Common Iliac Artery with Drug-eluting Intraluminal Device, Percutaneous Endoscopic Approach |
| 047D4DZ | Dilation of Left Common Iliac Artery with Intraluminal Device, Percutaneous Endoscopic Approach |
| 047D4ZZ | Dilation of Left Common Iliac Artery, Percutaneous Endoscopic Approach |
| 047E04Z | Dilation of Right Internal Iliac Artery with Drug-eluting Intraluminal Device, Open Approach |
| 047E0DZ | Dilation of Right Internal Iliac Artery with Intraluminal Device, Open Approach |
| 047E0ZZ | Dilation of Right Internal Iliac Artery, Open Approach |
| 047E34Z | Dilation of Right Internal Iliac Artery with Drug-eluting Intraluminal Device, Percutaneous Approach |
| 047E3DZ | Dilation of Right Internal Iliac Artery with Intraluminal Device, Percutaneous Approach |
| 047E3ZZ | Dilation of Right Internal Iliac Artery, Percutaneous Approach |
| 047E44Z | Dilation of Right Internal Iliac Artery with Drug-eluting Intraluminal Device, Percutaneous Endoscopic Approach |
| 047E4DZ | Dilation of Right Internal Iliac Artery with Intraluminal Device, Percutaneous Endoscopic Approach |
| 047E4ZZ | Dilation of Right Internal Iliac Artery, Percutaneous Endoscopic Approach |
| 047F04Z | Dilation of Left Internal Iliac Artery with Drug-eluting Intraluminal Device, Open Approach |
| 047F0DZ | Dilation of Left Internal Iliac Artery with Intraluminal Device, Open Approach |
| 047F0ZZ | Dilation of Left Internal Iliac Artery, Open Approach |
| 047F34Z | Dilation of Left Internal Iliac Artery with Drug-eluting Intraluminal Device, Percutaneous Approach |
| 047F3DZ | Dilation of Left Internal Iliac Artery with Intraluminal Device, Percutaneous Approach |
| 047F3ZZ | Dilation of Left Internal Iliac Artery, Percutaneous Approach |
| 047F44Z | Dilation of Left Internal Iliac Artery with Drug-eluting Intraluminal Device, Percutaneous Endoscopic Approach |
| 047F4DZ | Dilation of Left Internal Iliac Artery with Intraluminal Device, Percutaneous Endoscopic Approach |
| 047F4ZZ | Dilation of Left Internal Iliac Artery, Percutaneous Endoscopic Approach |
| 047H04Z | Dilation of Right External Iliac Artery with Drug-eluting Intraluminal Device, Open Approach |
| 047H0DZ | Dilation of Right External Iliac Artery with Intraluminal Device, Open Approach |
| 047H0ZZ | Dilation of Right External Iliac Artery, Open Approach |
| 047H34Z | Dilation of Right External Iliac Artery with Drug-eluting Intraluminal Device, Percutaneous Approach |
| 047H3DZ | Dilation of Right External Iliac Artery with Intraluminal Device, Percutaneous Approach |
| 047H3ZZ | Dilation of Right External Iliac Artery, Percutaneous Approach |
| 047H44Z | Dilation of Right External Iliac Artery with Drug-eluting Intraluminal Device, Percutaneous Endoscopic Approach |
| 047H4DZ | Dilation of Right External Iliac Artery with Intraluminal Device, Percutaneous Endoscopic Approach |
| 047H4ZZ | Dilation of Right External Iliac Artery, Percutaneous Endoscopic Approach |
| 047J04Z | Dilation of Left External Iliac Artery with Drug-eluting Intraluminal Device, Open Approach |
| 047J0DZ | Dilation of Left External Iliac Artery with Intraluminal Device, Open Approach |
| 047J0ZZ | Dilation of Left External Iliac Artery, Open Approach |
| 047J34Z | Dilation of Left External Iliac Artery with Drug-eluting Intraluminal Device, Percutaneous Approach |
| 047J3DZ | Dilation of Left External Iliac Artery with Intraluminal Device, Percutaneous Approach |
| 047J3ZZ | Dilation of Left External Iliac Artery, Percutaneous Approach |
| 047J44Z | Dilation of Left External Iliac Artery with Drug-eluting Intraluminal Device, Percutaneous Endoscopic Approach |
| 047J4DZ | Dilation of Left External Iliac Artery with Intraluminal Device, Percutaneous Endoscopic Approach |
| 047J4ZZ | Dilation of Left External Iliac Artery, Percutaneous Endoscopic Approach |
| 047K041 | Dilation of Right Femoral Artery with Drug-eluting Intraluminal Device, using Drug-Coated Balloon, Open Approach |
| 047K04Z | Dilation of Right Femoral Artery with Drug-eluting Intraluminal Device, Open Approach |
| 047K0D1 | Dilation of Right Femoral Artery with Intraluminal Device, using Drug-Coated Balloon, Open Approach |
| 047K0DZ | Dilation of Right Femoral Artery with Intraluminal Device, Open Approach |
| 047K0Z1 | Dilation of Right Femoral Artery using Drug-Coated Balloon, Open Approach |
| 047K0ZZ | Dilation of Right Femoral Artery, Open Approach |
| 047K341 | Dilation of Right Femoral Artery with Drug-eluting Intraluminal Device, using Drug-Coated Balloon, Percutaneous Approach |
| 047K34Z | Dilation of Right Femoral Artery with Drug-eluting Intraluminal Device, Percutaneous Approach |
| 047K3D1 | Dilation of Right Femoral Artery with Intraluminal Device, using Drug-Coated Balloon, Percutaneous Approach |
| 047K3DZ | Dilation of Right Femoral Artery with Intraluminal Device, Percutaneous Approach |
| 047K3Z1 | Dilation of Right Femoral Artery using Drug-Coated Balloon, Percutaneous Approach |
| 047K3ZZ | Dilation of Right Femoral Artery, Percutaneous Approach |
| 047K441 | Dilation of Right Femoral Artery with Drug-eluting Intraluminal Device, using Drug-Coated Balloon, Percutaneous Endoscopic Approach |
| 047K44Z | Dilation of Right Femoral Artery with Drug-eluting Intraluminal Device, Percutaneous Endoscopic Approach |
| 047K4D1 | Dilation of Right Femoral Artery with Intraluminal Device, using Drug-Coated Balloon, Percutaneous Endoscopic Approach |
| 047K4DZ | Dilation of Right Femoral Artery with Intraluminal Device, Percutaneous Endoscopic Approach |
| 047K4Z1 | Dilation of Right Femoral Artery using Drug-Coated Balloon, Percutaneous Endoscopic Approach |
| 047K4ZZ | Dilation of Right Femoral Artery, Percutaneous Endoscopic Approach |
| 047L041 | Dilation of Left Femoral Artery with Drug-eluting Intraluminal Device, using Drug-Coated Balloon, Open Approach |
| 047L04Z | Dilation of Left Femoral Artery with Drug-eluting Intraluminal Device, Open Approach |
| 047L0D1 | Dilation of Left Femoral Artery with Intraluminal Device, using Drug-Coated Balloon, Open Approach |
| 047L0DZ | Dilation of Left Femoral Artery with Intraluminal Device, Open Approach |
| 047L0Z1 | Dilation of Left Femoral Artery using Drug-Coated Balloon, Open Approach |
| 047L0ZZ | Dilation of Left Femoral Artery, Open Approach |
| 047L341 | Dilation of Left Femoral Artery with Drug-eluting Intraluminal Device, using Drug-Coated Balloon, Percutaneous Approach |
| 047L34Z | Dilation of Left Femoral Artery with Drug-eluting Intraluminal Device, Percutaneous Approach |
| 047L3D1 | Dilation of Left Femoral Artery with Intraluminal Device, using Drug-Coated Balloon, Percutaneous Approach |
| 047L3DZ | Dilation of Left Femoral Artery with Intraluminal Device, Percutaneous Approach |
| 047L3Z1 | Dilation of Left Femoral Artery using Drug-Coated Balloon, Percutaneous Approach |
| 047L3ZZ | Dilation of Left Femoral Artery, Percutaneous Approach |
| 047L441 | Dilation of Left Femoral Artery with Drug-eluting Intraluminal Device, using Drug-Coated Balloon, Percutaneous Endoscopic Approach |
| 047L44Z | Dilation of Left Femoral Artery with Drug-eluting Intraluminal Device, Percutaneous Endoscopic Approach |
| 047L4D1 | Dilation of Left Femoral Artery with Intraluminal Device, using Drug-Coated Balloon, Percutaneous Endoscopic Approach |
| 047L4DZ | Dilation of Left Femoral Artery with Intraluminal Device, Percutaneous Endoscopic Approach |
| 047L4Z1 | Dilation of Left Femoral Artery using Drug-Coated Balloon, Percutaneous Endoscopic Approach |
| 047L4ZZ | Dilation of Left Femoral Artery, Percutaneous Endoscopic Approach |
| 047M041 | Dilation of Right Popliteal Artery with Drug-eluting Intraluminal Device, using Drug-Coated Balloon, Open Approach |
| 047M04Z | Dilation of Right Popliteal Artery with Drug-eluting Intraluminal Device, Open Approach |
| 047M0D1 | Dilation of Right Popliteal Artery with Intraluminal Device, using Drug-Coated Balloon, Open Approach |
| 047M0DZ | Dilation of Right Popliteal Artery with Intraluminal Device, Open Approach |
| 047M0Z1 | Dilation of Right Popliteal Artery using Drug-Coated Balloon, Open Approach |
| 047M0ZZ | Dilation of Right Popliteal Artery, Open Approach |
| 047M341 | Dilation of Right Popliteal Artery with Drug-eluting Intraluminal Device, using Drug-Coated Balloon, Percutaneous Approach |
| 047M34Z | Dilation of Right Popliteal Artery with Drug-eluting Intraluminal Device, Percutaneous Approach |
| 047M3D1 | Dilation of Right Popliteal Artery with Intraluminal Device, using Drug-Coated Balloon, Percutaneous Approach |
| 047M3DZ | Dilation of Right Popliteal Artery with Intraluminal Device, Percutaneous Approach |
| 047M3Z1 | Dilation of Right Popliteal Artery using Drug-Coated Balloon, Percutaneous Approach |
| 047M3ZZ | Dilation of Right Popliteal Artery, Percutaneous Approach |
| 047M441 | Dilation of Right Popliteal Artery with Drug-eluting Intraluminal Device, using Drug-Coated Balloon, Percutaneous Endoscopic Approach |
| 047M44Z | Dilation of Right Popliteal Artery with Drug-eluting Intraluminal Device, Percutaneous Endoscopic Approach |
| 047M4D1 | Dilation of Right Popliteal Artery with Intraluminal Device, using Drug-Coated Balloon, Percutaneous Endoscopic Approach |
| 047M4DZ | Dilation of Right Popliteal Artery with Intraluminal Device, Percutaneous Endoscopic Approach |
| 047M4Z1 | Dilation of Right Popliteal Artery using Drug-Coated Balloon, Percutaneous Endoscopic Approach |
| 047M4ZZ | Dilation of Right Popliteal Artery, Percutaneous Endoscopic Approach |
| 047N041 | Dilation of Left Popliteal Artery with Drug-eluting Intraluminal Device, using Drug-Coated Balloon, Open Approach |
| 047N04Z | Dilation of Left Popliteal Artery with Drug-eluting Intraluminal Device, Open Approach |
| 047N0D1 | Dilation of Left Popliteal Artery with Intraluminal Device, using Drug-Coated Balloon, Open Approach |
| 047N0DZ | Dilation of Left Popliteal Artery with Intraluminal Device, Open Approach |
| 047N0Z1 | Dilation of Left Popliteal Artery using Drug-Coated Balloon, Open Approach |
| 047N0ZZ | Dilation of Left Popliteal Artery, Open Approach |
| 047N341 | Dilation of Left Popliteal Artery with Drug-eluting Intraluminal Device, using Drug-Coated Balloon, Percutaneous Approach |
| 047N34Z | Dilation of Left Popliteal Artery with Drug-eluting Intraluminal Device, Percutaneous Approach |
| 047N3D1 | Dilation of Left Popliteal Artery with Intraluminal Device, using Drug-Coated Balloon, Percutaneous Approach |
| 047N3DZ | Dilation of Left Popliteal Artery with Intraluminal Device, Percutaneous Approach |
| 047N3Z1 | Dilation of Left Popliteal Artery using Drug-Coated Balloon, Percutaneous Approach |
| 047N3ZZ | Dilation of Left Popliteal Artery, Percutaneous Approach |
| 047N441 | Dilation of Left Popliteal Artery with Drug-eluting Intraluminal Device, using Drug-Coated Balloon, Percutaneous Endoscopic Approach |
| 047N44Z | Dilation of Left Popliteal Artery with Drug-eluting Intraluminal Device, Percutaneous Endoscopic Approach |
| 047N4D1 | Dilation of Left Popliteal Artery with Intraluminal Device, using Drug-Coated Balloon, Percutaneous Endoscopic Approach |
| 047N4DZ | Dilation of Left Popliteal Artery with Intraluminal Device, Percutaneous Endoscopic Approach |
| 047N4Z1 | Dilation of Left Popliteal Artery using Drug-Coated Balloon, Percutaneous Endoscopic Approach |
| 047N4ZZ | Dilation of Left Popliteal Artery, Percutaneous Endoscopic Approach |
| 047P04Z | Dilation of Right Anterior Tibial Artery with Drug-eluting Intraluminal Device, Open Approach |
| 047P0DZ | Dilation of Right Anterior Tibial Artery with Intraluminal Device, Open Approach |
| 047P0ZZ | Dilation of Right Anterior Tibial Artery, Open Approach |
| 047P34Z | Dilation of Right Anterior Tibial Artery with Drug-eluting Intraluminal Device, Percutaneous Approach |
| 047P3DZ | Dilation of Right Anterior Tibial Artery with Intraluminal Device, Percutaneous Approach |
| 047P3ZZ | Dilation of Right Anterior Tibial Artery, Percutaneous Approach |
| 047P44Z | Dilation of Right Anterior Tibial Artery with Drug-eluting Intraluminal Device, Percutaneous Endoscopic Approach |
| 047P4DZ | Dilation of Right Anterior Tibial Artery with Intraluminal Device, Percutaneous Endoscopic Approach |
| 047P4ZZ | Dilation of Right Anterior Tibial Artery, Percutaneous Endoscopic Approach |
| 047Q04Z | Dilation of Left Anterior Tibial Artery with Drug-eluting Intraluminal Device, Open Approach |
| 047Q0DZ | Dilation of Left Anterior Tibial Artery with Intraluminal Device, Open Approach |
| 047Q0ZZ | Dilation of Left Anterior Tibial Artery, Open Approach |
| 047Q34Z | Dilation of Left Anterior Tibial Artery with Drug-eluting Intraluminal Device, Percutaneous Approach |
| 047Q3DZ | Dilation of Left Anterior Tibial Artery with Intraluminal Device, Percutaneous Approach |
| 047Q3ZZ | Dilation of Left Anterior Tibial Artery, Percutaneous Approach |
| 047Q44Z | Dilation of Left Anterior Tibial Artery with Drug-eluting Intraluminal Device, Percutaneous Endoscopic Approach |
| 047Q4DZ | Dilation of Left Anterior Tibial Artery with Intraluminal Device, Percutaneous Endoscopic Approach |
| 047Q4ZZ | Dilation of Left Anterior Tibial Artery, Percutaneous Endoscopic Approach |
| 047R04Z | Dilation of Right Posterior Tibial Artery with Drug-eluting Intraluminal Device, Open Approach |
| 047R0DZ | Dilation of Right Posterior Tibial Artery with Intraluminal Device, Open Approach |
| 047R0ZZ | Dilation of Right Posterior Tibial Artery, Open Approach |
| 047R34Z | Dilation of Right Posterior Tibial Artery with Drug-eluting Intraluminal Device, Percutaneous Approach |
| 047R3DZ | Dilation of Right Posterior Tibial Artery with Intraluminal Device, Percutaneous Approach |
| 047R3ZZ | Dilation of Right Posterior Tibial Artery, Percutaneous Approach |
| 047R44Z | Dilation of Right Posterior Tibial Artery with Drug-eluting Intraluminal Device, Percutaneous Endoscopic Approach |
| 047R4DZ | Dilation of Right Posterior Tibial Artery with Intraluminal Device, Percutaneous Endoscopic Approach |
| 047R4ZZ | Dilation of Right Posterior Tibial Artery, Percutaneous Endoscopic Approach |
| 047S04Z | Dilation of Left Posterior Tibial Artery with Drug-eluting Intraluminal Device, Open Approach |
| 047S0DZ | Dilation of Left Posterior Tibial Artery with Intraluminal Device, Open Approach |
| 047S0ZZ | Dilation of Left Posterior Tibial Artery, Open Approach |
| 047S34Z | Dilation of Left Posterior Tibial Artery with Drug-eluting Intraluminal Device, Percutaneous Approach |
| 047S3DZ | Dilation of Left Posterior Tibial Artery with Intraluminal Device, Percutaneous Approach |
| 047S3ZZ | Dilation of Left Posterior Tibial Artery, Percutaneous Approach |
| 047S44Z | Dilation of Left Posterior Tibial Artery with Drug-eluting Intraluminal Device, Percutaneous Endoscopic Approach |
| 047S4DZ | Dilation of Left Posterior Tibial Artery with Intraluminal Device, Percutaneous Endoscopic Approach |
| 047S4ZZ | Dilation of Left Posterior Tibial Artery, Percutaneous Endoscopic Approach |
| 047T04Z | Dilation of Right Peroneal Artery with Drug-eluting Intraluminal Device, Open Approach |
| 047T0DZ | Dilation of Right Peroneal Artery with Intraluminal Device, Open Approach |
| 047T0ZZ | Dilation of Right Peroneal Artery, Open Approach |
| 047T34Z | Dilation of Right Peroneal Artery with Drug-eluting Intraluminal Device, Percutaneous Approach |
| 047T3DZ | Dilation of Right Peroneal Artery with Intraluminal Device, Percutaneous Approach |
| 047T3ZZ | Dilation of Right Peroneal Artery, Percutaneous Approach |
| 047T44Z | Dilation of Right Peroneal Artery with Drug-eluting Intraluminal Device, Percutaneous Endoscopic Approach |
| 047T4DZ | Dilation of Right Peroneal Artery with Intraluminal Device, Percutaneous Endoscopic Approach |
| 047T4ZZ | Dilation of Right Peroneal Artery, Percutaneous Endoscopic Approach |
| 047U04Z | Dilation of Left Peroneal Artery with Drug-eluting Intraluminal Device, Open Approach |
| 047U0DZ | Dilation of Left Peroneal Artery with Intraluminal Device, Open Approach |
| 047U0ZZ | Dilation of Left Peroneal Artery, Open Approach |
| 047U34Z | Dilation of Left Peroneal Artery with Drug-eluting Intraluminal Device, Percutaneous Approach |
| 047U3DZ | Dilation of Left Peroneal Artery with Intraluminal Device, Percutaneous Approach |
| 047U3ZZ | Dilation of Left Peroneal Artery, Percutaneous Approach |
| 047U44Z | Dilation of Left Peroneal Artery with Drug-eluting Intraluminal Device, Percutaneous Endoscopic Approach |
| 047U4DZ | Dilation of Left Peroneal Artery with Intraluminal Device, Percutaneous Endoscopic Approach |
| 047U4ZZ | Dilation of Left Peroneal Artery, Percutaneous Endoscopic Approach |
| 047V04Z | Dilation of Right Foot Artery with Drug-eluting Intraluminal Device, Open Approach |
| 047V0DZ | Dilation of Right Foot Artery with Intraluminal Device, Open Approach |
| 047V0ZZ | Dilation of Right Foot Artery, Open Approach |
| 047V34Z | Dilation of Right Foot Artery with Drug-eluting Intraluminal Device, Percutaneous Approach |
| 047V3DZ | Dilation of Right Foot Artery with Intraluminal Device, Percutaneous Approach |
| 047V3ZZ | Dilation of Right Foot Artery, Percutaneous Approach |
| 047V44Z | Dilation of Right Foot Artery with Drug-eluting Intraluminal Device, Percutaneous Endoscopic Approach |
| 047V4DZ | Dilation of Right Foot Artery with Intraluminal Device, Percutaneous Endoscopic Approach |
| 047V4ZZ | Dilation of Right Foot Artery, Percutaneous Endoscopic Approach |
| 047W04Z | Dilation of Left Foot Artery with Drug-eluting Intraluminal Device, Open Approach |
| 047W0DZ | Dilation of Left Foot Artery with Intraluminal Device, Open Approach |
| 047W0ZZ | Dilation of Left Foot Artery, Open Approach |
| 047W34Z | Dilation of Left Foot Artery with Drug-eluting Intraluminal Device, Percutaneous Approach |
| 047W3DZ | Dilation of Left Foot Artery with Intraluminal Device, Percutaneous Approach |
| 047W3ZZ | Dilation of Left Foot Artery, Percutaneous Approach |
| 047W44Z | Dilation of Left Foot Artery with Drug-eluting Intraluminal Device, Percutaneous Endoscopic Approach |
| 047W4DZ | Dilation of Left Foot Artery with Intraluminal Device, Percutaneous Endoscopic Approach |
| 047W4ZZ | Dilation of Left Foot Artery, Percutaneous Endoscopic Approach |
| 031209B | Bypass Innominate Artery to Left Lower Leg Artery with Autologous Venous Tissue, Open Approach |
| 031209C | Bypass Innominate Artery to Bilateral Lower Leg Artery with Autologous Venous Tissue, Open Approach |
| 03120A6 | Bypass Innominate Artery to Right Upper Leg Artery with Autologous Arterial Tissue, Open Approach |
| 03120A7 | Bypass Innominate Artery to Left Upper Leg Artery with Autologous Arterial Tissue, Open Approach |
| 03120A8 | Bypass Innominate Artery to Bilateral Upper Leg Artery with Autologous Arterial Tissue, Open Approach |
| 03120A9 | Bypass Innominate Artery to Right Lower Leg Artery with Autologous Arterial Tissue, Open Approach |
| 03120AB | Bypass Innominate Artery to Left Lower Leg Artery with Autologous Arterial Tissue, Open Approach |
| 03120AC | Bypass Innominate Artery to Bilateral Lower Leg Artery with Autologous Arterial Tissue, Open Approach |
| 03120J6 | Bypass Innominate Artery to Right Upper Leg Artery with Synthetic Substitute, Open Approach |
| 03120J7 | Bypass Innominate Artery to Left Upper Leg Artery with Synthetic Substitute, Open Approach |
| 03120J8 | Bypass Innominate Artery to Bilateral Upper Leg Artery with Synthetic Substitute, Open Approach |
| 03120J9 | Bypass Innominate Artery to Right Lower Leg Artery with Synthetic Substitute, Open Approach |
| 03120JB | Bypass Innominate Artery to Left Lower Leg Artery with Synthetic Substitute, Open Approach |
| 03120JC | Bypass Innominate Artery to Bilateral Lower Leg Artery with Synthetic Substitute, Open Approach |
| 03120K6 | Bypass Innominate Artery to Right Upper Leg Artery with Nonautologous Tissue Substitute, Open Approach |
| 03120K7 | Bypass Innominate Artery to Left Upper Leg Artery with Nonautologous Tissue Substitute, Open Approach |
| 03120K8 | Bypass Innominate Artery to Bilateral Upper Leg Artery with Nonautologous Tissue Substitute, Open Approach |
| 03120K9 | Bypass Innominate Artery to Right Lower Leg Artery with Nonautologous Tissue Substitute, Open Approach |
| 03120KB | Bypass Innominate Artery to Left Lower Leg Artery with Nonautologous Tissue Substitute, Open Approach |
| 03120KC | Bypass Innominate Artery to Bilateral Lower Leg Artery with Nonautologous Tissue Substitute, Open Approach |
| 03120Z6 | Bypass Innominate Artery to Right Upper Leg Artery, Open Approach |
| 03120Z7 | Bypass Innominate Artery to Left Upper Leg Artery, Open Approach |
| 03120Z8 | Bypass Innominate Artery to Bilateral Upper Leg Artery, Open Approach |
| 03120Z9 | Bypass Innominate Artery to Right Lower Leg Artery, Open Approach |
| 03120ZB | Bypass Innominate Artery to Left Lower Leg Artery, Open Approach |
| 03120ZC | Bypass Innominate Artery to Bilateral Lower Leg Artery, Open Approach |
| 031309B | Bypass Right Subclavian Artery to Left Lower Leg Artery with Autologous Venous Tissue, Open Approach |
| 031309C | Bypass Right Subclavian Artery to Bilateral Lower Leg Artery with Autologous Venous Tissue, Open Approach |
| 03130A6 | Bypass Right Subclavian Artery to Right Upper Leg Artery with Autologous Arterial Tissue, Open Approach |
| 03130A7 | Bypass Right Subclavian Artery to Left Upper Leg Artery with Autologous Arterial Tissue, Open Approach |
| 03130A8 | Bypass Right Subclavian Artery to Bilateral Upper Leg Artery with Autologous Arterial Tissue, Open Approach |
| 03130A9 | Bypass Right Subclavian Artery to Right Lower Leg Artery with Autologous Arterial Tissue, Open Approach |
| 03130AB | Bypass Right Subclavian Artery to Left Lower Leg Artery with Autologous Arterial Tissue, Open Approach |
| 03130AC | Bypass Right Subclavian Artery to Bilateral Lower Leg Artery with Autologous Arterial Tissue, Open Approach |
| 03130J6 | Bypass Right Subclavian Artery to Right Upper Leg Artery with Synthetic Substitute, Open Approach |
| 03130J7 | Bypass Right Subclavian Artery to Left Upper Leg Artery with Synthetic Substitute, Open Approach |
| 03130J8 | Bypass Right Subclavian Artery to Bilateral Upper Leg Artery with Synthetic Substitute, Open Approach |
| 03130J9 | Bypass Right Subclavian Artery to Right Lower Leg Artery with Synthetic Substitute, Open Approach |
| 03130JB | Bypass Right Subclavian Artery to Left Lower Leg Artery with Synthetic Substitute, Open Approach |
| 03130JC | Bypass Right Subclavian Artery to Bilateral Lower Leg Artery with Synthetic Substitute, Open Approach |
| 03130K6 | Bypass Right Subclavian Artery to Right Upper Leg Artery with Nonautologous Tissue Substitute, Open Approach |
| 03130K7 | Bypass Right Subclavian Artery to Left Upper Leg Artery with Nonautologous Tissue Substitute, Open Approach |
| 03130K8 | Bypass Right Subclavian Artery to Bilateral Upper Leg Artery with Nonautologous Tissue Substitute, Open Approach |
| 03130K9 | Bypass Right Subclavian Artery to Right Lower Leg Artery with Nonautologous Tissue Substitute, Open Approach |
| 03130KB | Bypass Right Subclavian Artery to Left Lower Leg Artery with Nonautologous Tissue Substitute, Open Approach |
| 03130KC | Bypass Right Subclavian Artery to Bilateral Lower Leg Artery with Nonautologous Tissue Substitute, Open Approach |
| 041009B | Bypass Abdominal Aorta to Left Internal Iliac Artery with Autologous Venous Tissue, Open Approach |
| 041009C | Bypass Abdominal Aorta to Bilateral Internal Iliac Arteries with Autologous Venous Tissue, Open Approach |
| 041009D | Bypass Abdominal Aorta to Right External Iliac Artery with Autologous Venous Tissue, Open Approach |
| 041009F | Bypass Abdominal Aorta to Left External Iliac Artery with Autologous Venous Tissue, Open Approach |
| 041009G | Bypass Abdominal Aorta to Bilateral External Iliac Arteries with Autologous Venous Tissue, Open Approach |
| 041009H | Bypass Abdominal Aorta to Right Femoral Artery with Autologous Venous Tissue, Open Approach |
| 041009J | Bypass Abdominal Aorta to Left Femoral Artery with Autologous Venous Tissue, Open Approach |
| 041009K | Bypass Abdominal Aorta to Bilateral Femoral Arteries with Autologous Venous Tissue, Open Approach |
| 041009Q | Bypass Abdominal Aorta to Lower Extremity Artery with Autologous Venous Tissue, Open Approach |
| 041009R | Bypass Abdominal Aorta to Lower Artery with Autologous Venous Tissue, Open Approach |
| 04100A6 | Bypass Abdominal Aorta to Right Common Iliac Artery with Autologous Arterial Tissue, Open Approach |
| 04100A7 | Bypass Abdominal Aorta to Left Common Iliac Artery with Autologous Arterial Tissue, Open Approach |
| 04100A8 | Bypass Abdominal Aorta to Bilateral Common Iliac Arteries with Autologous Arterial Tissue, Open Approach |
| 04100A9 | Bypass Abdominal Aorta to Right Internal Iliac Artery with Autologous Arterial Tissue, Open Approach |
| 04100AB | Bypass Abdominal Aorta to Left Internal Iliac Artery with Autologous Arterial Tissue, Open Approach |
| 04100AC | Bypass Abdominal Aorta to Bilateral Internal Iliac Arteries with Autologous Arterial Tissue, Open Approach |
| 04100AD | Bypass Abdominal Aorta to Right External Iliac Artery with Autologous Arterial Tissue, Open Approach |
| 04100AF | Bypass Abdominal Aorta to Left External Iliac Artery with Autologous Arterial Tissue, Open Approach |
| 04100AG | Bypass Abdominal Aorta to Bilateral External Iliac Arteries with Autologous Arterial Tissue, Open Approach |
| 04100AH | Bypass Abdominal Aorta to Right Femoral Artery with Autologous Arterial Tissue, Open Approach |
| 04100AJ | Bypass Abdominal Aorta to Left Femoral Artery with Autologous Arterial Tissue, Open Approach |
| 04100AK | Bypass Abdominal Aorta to Bilateral Femoral Arteries with Autologous Arterial Tissue, Open Approach |
| 04100AQ | Bypass Abdominal Aorta to Lower Extremity Artery with Autologous Arterial Tissue, Open Approach |
| 04100AR | Bypass Abdominal Aorta to Lower Artery with Autologous Arterial Tissue, Open Approach |
| 04100J6 | Bypass Abdominal Aorta to Right Common Iliac Artery with Synthetic Substitute, Open Approach |
| 04100J7 | Bypass Abdominal Aorta to Left Common Iliac Artery with Synthetic Substitute, Open Approach |
| 04100J8 | Bypass Abdominal Aorta to Bilateral Common Iliac Arteries with Synthetic Substitute, Open Approach |
| 04100J9 | Bypass Abdominal Aorta to Right Internal Iliac Artery with Synthetic Substitute, Open Approach |
| 04100JB | Bypass Abdominal Aorta to Left Internal Iliac Artery with Synthetic Substitute, Open Approach |
| 04100JC | Bypass Abdominal Aorta to Bilateral Internal Iliac Arteries with Synthetic Substitute, Open Approach |
| 04100JD | Bypass Abdominal Aorta to Right External Iliac Artery with Synthetic Substitute, Open Approach |
| 04100JF | Bypass Abdominal Aorta to Left External Iliac Artery with Synthetic Substitute, Open Approach |
| 04100JG | Bypass Abdominal Aorta to Bilateral External Iliac Arteries with Synthetic Substitute, Open Approach |
| 04100JH | Bypass Abdominal Aorta to Right Femoral Artery with Synthetic Substitute, Open Approach |
| 04100JJ | Bypass Abdominal Aorta to Left Femoral Artery with Synthetic Substitute, Open Approach |
| 04100JK | Bypass Abdominal Aorta to Bilateral Femoral Arteries with Synthetic Substitute, Open Approach |
| 04100JQ | Bypass Abdominal Aorta to Lower Extremity Artery with Synthetic Substitute, Open Approach |
| 04100JR | Bypass Abdominal Aorta to Lower Artery with Synthetic Substitute, Open Approach |
| 04100K6 | Bypass Abdominal Aorta to Right Common Iliac Artery with Nonautologous Tissue Substitute, Open Approach |
| 04100K7 | Bypass Abdominal Aorta to Left Common Iliac Artery with Nonautologous Tissue Substitute, Open Approach |
| 04100K8 | Bypass Abdominal Aorta to Bilateral Common Iliac Arteries with Nonautologous Tissue Substitute, Open Approach |
| 04100K9 | Bypass Abdominal Aorta to Right Internal Iliac Artery with Nonautologous Tissue Substitute, Open Approach |
| 04100KB | Bypass Abdominal Aorta to Left Internal Iliac Artery with Nonautologous Tissue Substitute, Open Approach |
| 04100KC | Bypass Abdominal Aorta to Bilateral Internal Iliac Arteries with Nonautologous Tissue Substitute, Open Approach |
| 04100KD | Bypass Abdominal Aorta to Right External Iliac Artery with Nonautologous Tissue Substitute, Open Approach |
| 04100KF | Bypass Abdominal Aorta to Left External Iliac Artery with Nonautologous Tissue Substitute, Open Approach |
| 04100KG | Bypass Abdominal Aorta to Bilateral External Iliac Arteries with Nonautologous Tissue Substitute, Open Approach |
| 04100KH | Bypass Abdominal Aorta to Right Femoral Artery with Nonautologous Tissue Substitute, Open Approach |
| 04100KJ | Bypass Abdominal Aorta to Left Femoral Artery with Nonautologous Tissue Substitute, Open Approach |
| 04100KK | Bypass Abdominal Aorta to Bilateral Femoral Arteries with Nonautologous Tissue Substitute, Open Approach |
| 04100KQ | Bypass Abdominal Aorta to Lower Extremity Artery with Nonautologous Tissue Substitute, Open Approach |
| 04100KR | Bypass Abdominal Aorta to Lower Artery with Nonautologous Tissue Substitute, Open Approach |
| 04100Z6 | Bypass Abdominal Aorta to Right Common Iliac Artery, Open Approach |
| 04100Z7 | Bypass Abdominal Aorta to Left Common Iliac Artery, Open Approach |
| 04100Z8 | Bypass Abdominal Aorta to Bilateral Common Iliac Arteries, Open Approach |
| 04100Z9 | Bypass Abdominal Aorta to Right Internal Iliac Artery, Open Approach |
| 04100ZB | Bypass Abdominal Aorta to Left Internal Iliac Artery, Open Approach |
| 04100ZC | Bypass Abdominal Aorta to Bilateral Internal Iliac Arteries, Open Approach |
| 04100ZD | Bypass Abdominal Aorta to Right External Iliac Artery, Open Approach |
| 04100ZF | Bypass Abdominal Aorta to Left External Iliac Artery, Open Approach |
| 04100ZG | Bypass Abdominal Aorta to Bilateral External Iliac Arteries, Open Approach |
| 04100ZH | Bypass Abdominal Aorta to Right Femoral Artery, Open Approach |
| 04100ZJ | Bypass Abdominal Aorta to Left Femoral Artery, Open Approach |
| 04100ZK | Bypass Abdominal Aorta to Bilateral Femoral Arteries, Open Approach |
| 04100ZQ | Bypass Abdominal Aorta to Lower Extremity Artery, Open Approach |
| 04100ZR | Bypass Abdominal Aorta to Lower Artery, Open Approach |
| 041049B | Bypass Abdominal Aorta to Left Internal Iliac Artery with Autologous Venous Tissue, Percutaneous Endoscopic Approach |
| 041049C | Bypass Abdominal Aorta to Bilateral Internal Iliac Arteries with Autologous Venous Tissue, Percutaneous Endoscopic Approach |
| 041049D | Bypass Abdominal Aorta to Right External Iliac Artery with Autologous Venous Tissue, Percutaneous Endoscopic Approach |
| 041049F | Bypass Abdominal Aorta to Left External Iliac Artery with Autologous Venous Tissue, Percutaneous Endoscopic Approach |
| 041049G | Bypass Abdominal Aorta to Bilateral External Iliac Arteries with Autologous Venous Tissue, Percutaneous Endoscopic Approach |
| 041049H | Bypass Abdominal Aorta to Right Femoral Artery with Autologous Venous Tissue, Percutaneous Endoscopic Approach |
| 041049J | Bypass Abdominal Aorta to Left Femoral Artery with Autologous Venous Tissue, Percutaneous Endoscopic Approach |
| 041049K | Bypass Abdominal Aorta to Bilateral Femoral Arteries with Autologous Venous Tissue, Percutaneous Endoscopic Approach |
| 041049Q | Bypass Abdominal Aorta to Lower Extremity Artery with Autologous Venous Tissue, Percutaneous Endoscopic Approach |
| 041049R | Bypass Abdominal Aorta to Lower Artery with Autologous Venous Tissue, Percutaneous Endoscopic Approach |
| 04104A6 | Bypass Abdominal Aorta to Right Common Iliac Artery with Autologous Arterial Tissue, Percutaneous Endoscopic Approach |
| 04104A7 | Bypass Abdominal Aorta to Left Common Iliac Artery with Autologous Arterial Tissue, Percutaneous Endoscopic Approach |
| 04104A8 | Bypass Abdominal Aorta to Bilateral Common Iliac Arteries with Autologous Arterial Tissue, Percutaneous Endoscopic Approach |
| 04104A9 | Bypass Abdominal Aorta to Right Internal Iliac Artery with Autologous Arterial Tissue, Percutaneous Endoscopic Approach |
| 04104AB | Bypass Abdominal Aorta to Left Internal Iliac Artery with Autologous Arterial Tissue, Percutaneous Endoscopic Approach |
| 04104AC | Bypass Abdominal Aorta to Bilateral Internal Iliac Arteries with Autologous Arterial Tissue, Percutaneous Endoscopic Approach |
| 04104AD | Bypass Abdominal Aorta to Right External Iliac Artery with Autologous Arterial Tissue, Percutaneous Endoscopic Approach |
| 04104AF | Bypass Abdominal Aorta to Left External Iliac Artery with Autologous Arterial Tissue, Percutaneous Endoscopic Approach |
| 04104AG | Bypass Abdominal Aorta to Bilateral External Iliac Arteries with Autologous Arterial Tissue, Percutaneous Endoscopic Approach |
| 04104AH | Bypass Abdominal Aorta to Right Femoral Artery with Autologous Arterial Tissue, Percutaneous Endoscopic Approach |
| 04104AJ | Bypass Abdominal Aorta to Left Femoral Artery with Autologous Arterial Tissue, Percutaneous Endoscopic Approach |
| 04104AK | Bypass Abdominal Aorta to Bilateral Femoral Arteries with Autologous Arterial Tissue, Percutaneous Endoscopic Approach |
| 04104AQ | Bypass Abdominal Aorta to Lower Extremity Artery with Autologous Arterial Tissue, Percutaneous Endoscopic Approach |
| 04104AR | Bypass Abdominal Aorta to Lower Artery with Autologous Arterial Tissue, Percutaneous Endoscopic Approach |
| 04104J6 | Bypass Abdominal Aorta to Right Common Iliac Artery with Synthetic Substitute, Percutaneous Endoscopic Approach |
| 04104J7 | Bypass Abdominal Aorta to Left Common Iliac Artery with Synthetic Substitute, Percutaneous Endoscopic Approach |
| 04104J8 | Bypass Abdominal Aorta to Bilateral Common Iliac Arteries with Synthetic Substitute, Percutaneous Endoscopic Approach |
| 04104J9 | Bypass Abdominal Aorta to Right Internal Iliac Artery with Synthetic Substitute, Percutaneous Endoscopic Approach |
| 04104JB | Bypass Abdominal Aorta to Left Internal Iliac Artery with Synthetic Substitute, Percutaneous Endoscopic Approach |
| 04104JC | Bypass Abdominal Aorta to Bilateral Internal Iliac Arteries with Synthetic Substitute, Percutaneous Endoscopic Approach |
| 04104JD | Bypass Abdominal Aorta to Right External Iliac Artery with Synthetic Substitute, Percutaneous Endoscopic Approach |
| 04104JF | Bypass Abdominal Aorta to Left External Iliac Artery with Synthetic Substitute, Percutaneous Endoscopic Approach |
| 04104JG | Bypass Abdominal Aorta to Bilateral External Iliac Arteries with Synthetic Substitute, Percutaneous Endoscopic Approach |
| 04104JH | Bypass Abdominal Aorta to Right Femoral Artery with Synthetic Substitute, Percutaneous Endoscopic Approach |
| 04104JJ | Bypass Abdominal Aorta to Left Femoral Artery with Synthetic Substitute, Percutaneous Endoscopic Approach |
| 04104JK | Bypass Abdominal Aorta to Bilateral Femoral Arteries with Synthetic Substitute, Percutaneous Endoscopic Approach |
| 04104JQ | Bypass Abdominal Aorta to Lower Extremity Artery with Synthetic Substitute, Percutaneous Endoscopic Approach |
| 04104JR | Bypass Abdominal Aorta to Lower Artery with Synthetic Substitute, Percutaneous Endoscopic Approach |
| 04104K6 | Bypass Abdominal Aorta to Right Common Iliac Artery with Nonautologous Tissue Substitute, Percutaneous Endoscopic Approach |
| 04104K7 | Bypass Abdominal Aorta to Left Common Iliac Artery with Nonautologous Tissue Substitute, Percutaneous Endoscopic Approach |
| 04104K8 | Bypass Abdominal Aorta to Bilateral Common Iliac Arteries with Nonautologous Tissue Substitute, Percutaneous Endoscopic Approach |
| 04104K9 | Bypass Abdominal Aorta to Right Internal Iliac Artery with Nonautologous Tissue Substitute, Percutaneous Endoscopic Approach |
| 04104KB | Bypass Abdominal Aorta to Left Internal Iliac Artery with Nonautologous Tissue Substitute, Percutaneous Endoscopic Approach |
| 04104KC | Bypass Abdominal Aorta to Bilateral Internal Iliac Arteries with Nonautologous Tissue Substitute, Percutaneous Endoscopic Approach |
| 04104KD | Bypass Abdominal Aorta to Right External Iliac Artery with Nonautologous Tissue Substitute, Percutaneous Endoscopic Approach |
| 04104KF | Bypass Abdominal Aorta to Left External Iliac Artery with Nonautologous Tissue Substitute, Percutaneous Endoscopic Approach |
| 04104KG | Bypass Abdominal Aorta to Bilateral External Iliac Arteries with Nonautologous Tissue Substitute, Percutaneous Endoscopic Approach |
| 04104KH | Bypass Abdominal Aorta to Right Femoral Artery with Nonautologous Tissue Substitute, Percutaneous Endoscopic Approach |
| 04104KJ | Bypass Abdominal Aorta to Left Femoral Artery with Nonautologous Tissue Substitute, Percutaneous Endoscopic Approach |
| 04104KK | Bypass Abdominal Aorta to Bilateral Femoral Arteries with Nonautologous Tissue Substitute, Percutaneous Endoscopic Approach |
| 04104KQ | Bypass Abdominal Aorta to Lower Extremity Artery with Nonautologous Tissue Substitute, Percutaneous Endoscopic Approach |
| 04104KR | Bypass Abdominal Aorta to Lower Artery with Nonautologous Tissue Substitute, Percutaneous Endoscopic Approach |
| 04104Z6 | Bypass Abdominal Aorta to Right Common Iliac Artery, Percutaneous Endoscopic Approach |
| 04104Z7 | Bypass Abdominal Aorta to Left Common Iliac Artery, Percutaneous Endoscopic Approach |
| 04104Z8 | Bypass Abdominal Aorta to Bilateral Common Iliac Arteries, Percutaneous Endoscopic Approach |
| 04104Z9 | Bypass Abdominal Aorta to Right Internal Iliac Artery, Percutaneous Endoscopic Approach |
| 04104ZB | Bypass Abdominal Aorta to Left Internal Iliac Artery, Percutaneous Endoscopic Approach |
| 04104ZC | Bypass Abdominal Aorta to Bilateral Internal Iliac Arteries, Percutaneous Endoscopic Approach |
| 04104ZD | Bypass Abdominal Aorta to Right External Iliac Artery, Percutaneous Endoscopic Approach |
| 04104ZF | Bypass Abdominal Aorta to Left External Iliac Artery, Percutaneous Endoscopic Approach |
| 04104ZG | Bypass Abdominal Aorta to Bilateral External Iliac Arteries, Percutaneous Endoscopic Approach |
| 04104ZH | Bypass Abdominal Aorta to Right Femoral Artery, Percutaneous Endoscopic Approach |
| 04104ZJ | Bypass Abdominal Aorta to Left Femoral Artery, Percutaneous Endoscopic Approach |
| 04104ZK | Bypass Abdominal Aorta to Bilateral Femoral Arteries, Percutaneous Endoscopic Approach |
| 04104ZQ | Bypass Abdominal Aorta to Lower Extremity Artery, Percutaneous Endoscopic Approach |
| 04104ZR | Bypass Abdominal Aorta to Lower Artery, Percutaneous Endoscopic Approach |
| 041C09H | Bypass Right Common Iliac Artery to Right Femoral Artery with Autologous Venous Tissue, Open Approach |
| 041C09J | Bypass Right Common Iliac Artery to Left Femoral Artery with Autologous Venous Tissue, Open Approach |
| 041C09K | Bypass Right Common Iliac Artery to Bilateral Femoral Arteries with Autologous Venous Tissue, Open Approach |
| 041C0AH | Bypass Right Common Iliac Artery to Right Femoral Artery with Autologous Arterial Tissue, Open Approach |
| 041C0AJ | Bypass Right Common Iliac Artery to Left Femoral Artery with Autologous Arterial Tissue, Open Approach |
| 041C0AK | Bypass Right Common Iliac Artery to Bilateral Femoral Arteries with Autologous Arterial Tissue, Open Approach |
| 041C0JH | Bypass Right Common Iliac Artery to Right Femoral Artery with Synthetic Substitute, Open Approach |
| 041C0JJ | Bypass Right Common Iliac Artery to Left Femoral Artery with Synthetic Substitute, Open Approach |
| 041C0JK | Bypass Right Common Iliac Artery to Bilateral Femoral Arteries with Synthetic Substitute, Open Approach |
| 041C0KH | Bypass Right Common Iliac Artery to Right Femoral Artery with Nonautologous Tissue Substitute, Open Approach |
| 041C0KJ | Bypass Right Common Iliac Artery to Left Femoral Artery with Nonautologous Tissue Substitute, Open Approach |
| 041C0KK | Bypass Right Common Iliac Artery to Bilateral Femoral Arteries with Nonautologous Tissue Substitute, Open Approach |
| 041C0ZH | Bypass Right Common Iliac Artery to Right Femoral Artery, Open Approach |
| 041C0ZJ | Bypass Right Common Iliac Artery to Left Femoral Artery, Open Approach |
| 041C0ZK | Bypass Right Common Iliac Artery to Bilateral Femoral Arteries, Open Approach |
| 041C49H | Bypass Right Common Iliac Artery to Right Femoral Artery with Autologous Venous Tissue, Percutaneous Endoscopic Approach |
| 041C49J | Bypass Right Common Iliac Artery to Left Femoral Artery with Autologous Venous Tissue, Percutaneous Endoscopic Approach |
| 041C49K | Bypass Right Common Iliac Artery to Bilateral Femoral Arteries with Autologous Venous Tissue, Percutaneous Endoscopic Approach |
| 041C4AH | Bypass Right Common Iliac Artery to Right Femoral Artery with Autologous Arterial Tissue, Percutaneous Endoscopic Approach |
| 041C4AJ | Bypass Right Common Iliac Artery to Left Femoral Artery with Autologous Arterial Tissue, Percutaneous Endoscopic Approach |
| 041C4AK | Bypass Right Common Iliac Artery to Bilateral Femoral Arteries with Autologous Arterial Tissue, Percutaneous Endoscopic Approach |
| 041C4JH | Bypass Right Common Iliac Artery to Right Femoral Artery with Synthetic Substitute, Percutaneous Endoscopic Approach |
| 041C4JJ | Bypass Right Common Iliac Artery to Left Femoral Artery with Synthetic Substitute, Percutaneous Endoscopic Approach |
| 041C4JK | Bypass Right Common Iliac Artery to Bilateral Femoral Arteries with Synthetic Substitute, Percutaneous Endoscopic Approach |
| 041C4KH | Bypass Right Common Iliac Artery to Right Femoral Artery with Nonautologous Tissue Substitute, Percutaneous Endoscopic Approach |
| 041C4KJ | Bypass Right Common Iliac Artery to Left Femoral Artery with Nonautologous Tissue Substitute, Percutaneous Endoscopic Approach |
| 041C4KK | Bypass Right Common Iliac Artery to Bilateral Femoral Arteries with Nonautologous Tissue Substitute, Percutaneous Endoscopic Approach |
| 041C4ZH | Bypass Right Common Iliac Artery to Right Femoral Artery, Percutaneous Endoscopic Approach |
| 041C4ZJ | Bypass Right Common Iliac Artery to Left Femoral Artery, Percutaneous Endoscopic Approach |
| 041C4ZK | Bypass Right Common Iliac Artery to Bilateral Femoral Arteries, Percutaneous Endoscopic Approach |
| 041D09H | Bypass Left Common Iliac Artery to Right Femoral Artery with Autologous Venous Tissue, Open Approach |
| 041D09J | Bypass Left Common Iliac Artery to Left Femoral Artery with Autologous Venous Tissue, Open Approach |
| 041D09K | Bypass Left Common Iliac Artery to Bilateral Femoral Arteries with Autologous Venous Tissue, Open Approach |
| 041D0AH | Bypass Left Common Iliac Artery to Right Femoral Artery with Autologous Arterial Tissue, Open Approach |
| 041D0AJ | Bypass Left Common Iliac Artery to Left Femoral Artery with Autologous Arterial Tissue, Open Approach |
| 041D0AK | Bypass Left Common Iliac Artery to Bilateral Femoral Arteries with Autologous Arterial Tissue, Open Approach |
| 041D0JH | Bypass Left Common Iliac Artery to Right Femoral Artery with Synthetic Substitute, Open Approach |
| 041D0JJ | Bypass Left Common Iliac Artery to Left Femoral Artery with Synthetic Substitute, Open Approach |
| 041D0JK | Bypass Left Common Iliac Artery to Bilateral Femoral Arteries with Synthetic Substitute, Open Approach |
| 041D0KH | Bypass Left Common Iliac Artery to Right Femoral Artery with Nonautologous Tissue Substitute, Open Approach |
| 041D0KJ | Bypass Left Common Iliac Artery to Left Femoral Artery with Nonautologous Tissue Substitute, Open Approach |
| 041D0KK | Bypass Left Common Iliac Artery to Bilateral Femoral Arteries with Nonautologous Tissue Substitute, Open Approach |
| 041D0ZH | Bypass Left Common Iliac Artery to Right Femoral Artery, Open Approach |
| 041D0ZJ | Bypass Left Common Iliac Artery to Left Femoral Artery, Open Approach |
| 041D0ZK | Bypass Left Common Iliac Artery to Bilateral Femoral Arteries, Open Approach |
| 041D49H | Bypass Left Common Iliac Artery to Right Femoral Artery with Autologous Venous Tissue, Percutaneous Endoscopic Approach |
| 041D49J | Bypass Left Common Iliac Artery to Left Femoral Artery with Autologous Venous Tissue, Percutaneous Endoscopic Approach |
| 041D49K | Bypass Left Common Iliac Artery to Bilateral Femoral Arteries with Autologous Venous Tissue, Percutaneous Endoscopic Approach |
| 041D4AH | Bypass Left Common Iliac Artery to Right Femoral Artery with Autologous Arterial Tissue, Percutaneous Endoscopic Approach |
| 041D4AJ | Bypass Left Common Iliac Artery to Left Femoral Artery with Autologous Arterial Tissue, Percutaneous Endoscopic Approach |
| 041D4AK | Bypass Left Common Iliac Artery to Bilateral Femoral Arteries with Autologous Arterial Tissue, Percutaneous Endoscopic Approach |
| 041D4JH | Bypass Left Common Iliac Artery to Right Femoral Artery with Synthetic Substitute, Percutaneous Endoscopic Approach |
| 041D4JJ | Bypass Left Common Iliac Artery to Left Femoral Artery with Synthetic Substitute, Percutaneous Endoscopic Approach |
| 041D4JK | Bypass Left Common Iliac Artery to Bilateral Femoral Arteries with Synthetic Substitute, Percutaneous Endoscopic Approach |
| 041D4KH | Bypass Left Common Iliac Artery to Right Femoral Artery with Nonautologous Tissue Substitute, Percutaneous Endoscopic Approach |
| 041D4KJ | Bypass Left Common Iliac Artery to Left Femoral Artery with Nonautologous Tissue Substitute, Percutaneous Endoscopic Approach |
| 041D4KK | Bypass Left Common Iliac Artery to Bilateral Femoral Arteries with Nonautologous Tissue Substitute, Percutaneous Endoscopic Approach |
| 041D4ZH | Bypass Left Common Iliac Artery to Right Femoral Artery, Percutaneous Endoscopic Approach |
| 041D4ZJ | Bypass Left Common Iliac Artery to Left Femoral Artery, Percutaneous Endoscopic Approach |
| 041D4ZK | Bypass Left Common Iliac Artery to Bilateral Femoral Arteries, Percutaneous Endoscopic Approach |
| 041E09H | Bypass Right Internal Iliac Artery to Right Femoral Artery with Autologous Venous Tissue, Open Approach |
| 041E09J | Bypass Right Internal Iliac Artery to Left Femoral Artery with Autologous Venous Tissue, Open Approach |
| 041E09K | Bypass Right Internal Iliac Artery to Bilateral Femoral Arteries with Autologous Venous Tissue, Open Approach |
| 041E0AH | Bypass Right Internal Iliac Artery to Right Femoral Artery with Autologous Arterial Tissue, Open Approach |
| 041E0AJ | Bypass Right Internal Iliac Artery to Left Femoral Artery with Autologous Arterial Tissue, Open Approach |
| 041E0AK | Bypass Right Internal Iliac Artery to Bilateral Femoral Arteries with Autologous Arterial Tissue, Open Approach |
| 041E0JH | Bypass Right Internal Iliac Artery to Right Femoral Artery with Synthetic Substitute, Open Approach |
| 041E0JJ | Bypass Right Internal Iliac Artery to Left Femoral Artery with Synthetic Substitute, Open Approach |
| 041E0JK | Bypass Right Internal Iliac Artery to Bilateral Femoral Arteries with Synthetic Substitute, Open Approach |
| 041E0KH | Bypass Right Internal Iliac Artery to Right Femoral Artery with Nonautologous Tissue Substitute, Open Approach |
| 041E0KJ | Bypass Right Internal Iliac Artery to Left Femoral Artery with Nonautologous Tissue Substitute, Open Approach |
| 041E0KK | Bypass Right Internal Iliac Artery to Bilateral Femoral Arteries with Nonautologous Tissue Substitute, Open Approach |
| 041E0ZH | Bypass Right Internal Iliac Artery to Right Femoral Artery, Open Approach |
| 041E0ZJ | Bypass Right Internal Iliac Artery to Left Femoral Artery, Open Approach |
| 041E0ZK | Bypass Right Internal Iliac Artery to Bilateral Femoral Arteries, Open Approach |
| 041E49H | Bypass Right Internal Iliac Artery to Right Femoral Artery with Autologous Venous Tissue, Percutaneous Endoscopic Approach |
| 041E49J | Bypass Right Internal Iliac Artery to Left Femoral Artery with Autologous Venous Tissue, Percutaneous Endoscopic Approach |
| 041E49K | Bypass Right Internal Iliac Artery to Bilateral Femoral Arteries with Autologous Venous Tissue, Percutaneous Endoscopic Approach |
| 041E4AH | Bypass Right Internal Iliac Artery to Right Femoral Artery with Autologous Arterial Tissue, Percutaneous Endoscopic Approach |
| 041E4AJ | Bypass Right Internal Iliac Artery to Left Femoral Artery with Autologous Arterial Tissue, Percutaneous Endoscopic Approach |
| 041E4AK | Bypass Right Internal Iliac Artery to Bilateral Femoral Arteries with Autologous Arterial Tissue, Percutaneous Endoscopic Approach |
| 041E4JH | Bypass Right Internal Iliac Artery to Right Femoral Artery with Synthetic Substitute, Percutaneous Endoscopic Approach |
| 041E4JJ | Bypass Right Internal Iliac Artery to Left Femoral Artery with Synthetic Substitute, Percutaneous Endoscopic Approach |
| 041E4JK | Bypass Right Internal Iliac Artery to Bilateral Femoral Arteries with Synthetic Substitute, Percutaneous Endoscopic Approach |
| 041E4KH | Bypass Right Internal Iliac Artery to Right Femoral Artery with Nonautologous Tissue Substitute, Percutaneous Endoscopic Approach |
| 041E4KJ | Bypass Right Internal Iliac Artery to Left Femoral Artery with Nonautologous Tissue Substitute, Percutaneous Endoscopic Approach |
| 041E4KK | Bypass Right Internal Iliac Artery to Bilateral Femoral Arteries with Nonautologous Tissue Substitute, Percutaneous Endoscopic Approach |
| 041E4ZH | Bypass Right Internal Iliac Artery to Right Femoral Artery, Percutaneous Endoscopic Approach |
| 041E4ZJ | Bypass Right Internal Iliac Artery to Left Femoral Artery, Percutaneous Endoscopic Approach |
| 041E4ZK | Bypass Right Internal Iliac Artery to Bilateral Femoral Arteries, Percutaneous Endoscopic Approach |
| 041F09H | Bypass Left Internal Iliac Artery to Right Femoral Artery with Autologous Venous Tissue, Open Approach |
| 041F09J | Bypass Left Internal Iliac Artery to Left Femoral Artery with Autologous Venous Tissue, Open Approach |
| 041F09K | Bypass Left Internal Iliac Artery to Bilateral Femoral Arteries with Autologous Venous Tissue, Open Approach |
| 041F0AH | Bypass Left Internal Iliac Artery to Right Femoral Artery with Autologous Arterial Tissue, Open Approach |
| 041F0AJ | Bypass Left Internal Iliac Artery to Left Femoral Artery with Autologous Arterial Tissue, Open Approach |
| 041F0AK | Bypass Left Internal Iliac Artery to Bilateral Femoral Arteries with Autologous Arterial Tissue, Open Approach |
| 041F0JH | Bypass Left Internal Iliac Artery to Right Femoral Artery with Synthetic Substitute, Open Approach |
| 041F0JJ | Bypass Left Internal Iliac Artery to Left Femoral Artery with Synthetic Substitute, Open Approach |
| 041F0JK | Bypass Left Internal Iliac Artery to Bilateral Femoral Arteries with Synthetic Substitute, Open Approach |
| 041F0KH | Bypass Left Internal Iliac Artery to Right Femoral Artery with Nonautologous Tissue Substitute, Open Approach |
| 041F0KJ | Bypass Left Internal Iliac Artery to Left Femoral Artery with Nonautologous Tissue Substitute, Open Approach |
| 041F0KK | Bypass Left Internal Iliac Artery to Bilateral Femoral Arteries with Nonautologous Tissue Substitute, Open Approach |
| 041F0ZH | Bypass Left Internal Iliac Artery to Right Femoral Artery, Open Approach |
| 041F0ZJ | Bypass Left Internal Iliac Artery to Left Femoral Artery, Open Approach |
| 041F0ZK | Bypass Left Internal Iliac Artery to Bilateral Femoral Arteries, Open Approach |
| 041F49H | Bypass Left Internal Iliac Artery to Right Femoral Artery with Autologous Venous Tissue, Percutaneous Endoscopic Approach |
| 041F49J | Bypass Left Internal Iliac Artery to Left Femoral Artery with Autologous Venous Tissue, Percutaneous Endoscopic Approach |
| 041F49K | Bypass Left Internal Iliac Artery to Bilateral Femoral Arteries with Autologous Venous Tissue, Percutaneous Endoscopic Approach |
| 041F4AH | Bypass Left Internal Iliac Artery to Right Femoral Artery with Autologous Arterial Tissue, Percutaneous Endoscopic Approach |
| 041F4AJ | Bypass Left Internal Iliac Artery to Left Femoral Artery with Autologous Arterial Tissue, Percutaneous Endoscopic Approach |
| 041F4AK | Bypass Left Internal Iliac Artery to Bilateral Femoral Arteries with Autologous Arterial Tissue, Percutaneous Endoscopic Approach |
| 041F4JH | Bypass Left Internal Iliac Artery to Right Femoral Artery with Synthetic Substitute, Percutaneous Endoscopic Approach |
| 041F4JJ | Bypass Left Internal Iliac Artery to Left Femoral Artery with Synthetic Substitute, Percutaneous Endoscopic Approach |
| 041F4JK | Bypass Left Internal Iliac Artery to Bilateral Femoral Arteries with Synthetic Substitute, Percutaneous Endoscopic Approach |
| 041F4KH | Bypass Left Internal Iliac Artery to Right Femoral Artery with Nonautologous Tissue Substitute, Percutaneous Endoscopic Approach |
| 041F4KJ | Bypass Left Internal Iliac Artery to Left Femoral Artery with Nonautologous Tissue Substitute, Percutaneous Endoscopic Approach |
| 041F4KK | Bypass Left Internal Iliac Artery to Bilateral Femoral Arteries with Nonautologous Tissue Substitute, Percutaneous Endoscopic Approach |
| 041F4ZH | Bypass Left Internal Iliac Artery to Right Femoral Artery, Percutaneous Endoscopic Approach |
| 041F4ZJ | Bypass Left Internal Iliac Artery to Left Femoral Artery, Percutaneous Endoscopic Approach |
| 041F4ZK | Bypass Left Internal Iliac Artery to Bilateral Femoral Arteries, Percutaneous Endoscopic Approach |
| 041H09H | Bypass Right External Iliac Artery to Right Femoral Artery with Autologous Venous Tissue, Open Approach |
| 041H09J | Bypass Right External Iliac Artery to Left Femoral Artery with Autologous Venous Tissue, Open Approach |
| 041H09K | Bypass Right External Iliac Artery to Bilateral Femoral Arteries with Autologous Venous Tissue, Open Approach |
| 041H0AH | Bypass Right External Iliac Artery to Right Femoral Artery with Autologous Arterial Tissue, Open Approach |
| 041H0AJ | Bypass Right External Iliac Artery to Left Femoral Artery with Autologous Arterial Tissue, Open Approach |
| 041H0AK | Bypass Right External Iliac Artery to Bilateral Femoral Arteries with Autologous Arterial Tissue, Open Approach |
| 041H0JH | Bypass Right External Iliac Artery to Right Femoral Artery with Synthetic Substitute, Open Approach |
| 041H0JJ | Bypass Right External Iliac Artery to Left Femoral Artery with Synthetic Substitute, Open Approach |
| 041H0JK | Bypass Right External Iliac Artery to Bilateral Femoral Arteries with Synthetic Substitute, Open Approach |
| 041H0KH | Bypass Right External Iliac Artery to Right Femoral Artery with Nonautologous Tissue Substitute, Open Approach |
| 041H0KJ | Bypass Right External Iliac Artery to Left Femoral Artery with Nonautologous Tissue Substitute, Open Approach |
| 041H0KK | Bypass Right External Iliac Artery to Bilateral Femoral Arteries with Nonautologous Tissue Substitute, Open Approach |
| 041H0ZH | Bypass Right External Iliac Artery to Right Femoral Artery, Open Approach |
| 041H0ZJ | Bypass Right External Iliac Artery to Left Femoral Artery, Open Approach |
| 041H0ZK | Bypass Right External Iliac Artery to Bilateral Femoral Arteries, Open Approach |
| 041H49H | Bypass Right External Iliac Artery to Right Femoral Artery with Autologous Venous Tissue, Percutaneous Endoscopic Approach |
| 041H49J | Bypass Right External Iliac Artery to Left Femoral Artery with Autologous Venous Tissue, Percutaneous Endoscopic Approach |
| 041H49K | Bypass Right External Iliac Artery to Bilateral Femoral Arteries with Autologous Venous Tissue, Percutaneous Endoscopic Approach |
| 041H4AH | Bypass Right External Iliac Artery to Right Femoral Artery with Autologous Arterial Tissue, Percutaneous Endoscopic Approach |
| 041H4AJ | Bypass Right External Iliac Artery to Left Femoral Artery with Autologous Arterial Tissue, Percutaneous Endoscopic Approach |
| 041H4AK | Bypass Right External Iliac Artery to Bilateral Femoral Arteries with Autologous Arterial Tissue, Percutaneous Endoscopic Approach |
| 041H4JH | Bypass Right External Iliac Artery to Right Femoral Artery with Synthetic Substitute, Percutaneous Endoscopic Approach |
| 041H4JJ | Bypass Right External Iliac Artery to Left Femoral Artery with Synthetic Substitute, Percutaneous Endoscopic Approach |
| 041H4JK | Bypass Right External Iliac Artery to Bilateral Femoral Arteries with Synthetic Substitute, Percutaneous Endoscopic Approach |
| 041H4KH | Bypass Right External Iliac Artery to Right Femoral Artery with Nonautologous Tissue Substitute, Percutaneous Endoscopic Approach |
| 041H4KJ | Bypass Right External Iliac Artery to Left Femoral Artery with Nonautologous Tissue Substitute, Percutaneous Endoscopic Approach |
| 041H4KK | Bypass Right External Iliac Artery to Bilateral Femoral Arteries with Nonautologous Tissue Substitute, Percutaneous Endoscopic Approach |
| 041H4ZH | Bypass Right External Iliac Artery to Right Femoral Artery, Percutaneous Endoscopic Approach |
| 041H4ZJ | Bypass Right External Iliac Artery to Left Femoral Artery, Percutaneous Endoscopic Approach |
| 041H4ZK | Bypass Right External Iliac Artery to Bilateral Femoral Arteries, Percutaneous Endoscopic Approach |
| 041J09H | Bypass Left External Iliac Artery to Right Femoral Artery with Autologous Venous Tissue, Open Approach |
| 041J09J | Bypass Left External Iliac Artery to Left Femoral Artery with Autologous Venous Tissue, Open Approach |
| 041J09K | Bypass Left External Iliac Artery to Bilateral Femoral Arteries with Autologous Venous Tissue, Open Approach |
| 041J0AH | Bypass Left External Iliac Artery to Right Femoral Artery with Autologous Arterial Tissue, Open Approach |
| 041J0AJ | Bypass Left External Iliac Artery to Left Femoral Artery with Autologous Arterial Tissue, Open Approach |
| 041J0AK | Bypass Left External Iliac Artery to Bilateral Femoral Arteries with Autologous Arterial Tissue, Open Approach |
| 041J0JH | Bypass Left External Iliac Artery to Right Femoral Artery with Synthetic Substitute, Open Approach |
| 041J0JJ | Bypass Left External Iliac Artery to Left Femoral Artery with Synthetic Substitute, Open Approach |
| 041J0JK | Bypass Left External Iliac Artery to Bilateral Femoral Arteries with Synthetic Substitute, Open Approach |
| 041J0KH | Bypass Left External Iliac Artery to Right Femoral Artery with Nonautologous Tissue Substitute, Open Approach |
| 041J0KJ | Bypass Left External Iliac Artery to Left Femoral Artery with Nonautologous Tissue Substitute, Open Approach |
| 041J0KK | Bypass Left External Iliac Artery to Bilateral Femoral Arteries with Nonautologous Tissue Substitute, Open Approach |
| 041J0ZH | Bypass Left External Iliac Artery to Right Femoral Artery, Open Approach |
| 041J0ZJ | Bypass Left External Iliac Artery to Left Femoral Artery, Open Approach |
| 041J0ZK | Bypass Left External Iliac Artery to Bilateral Femoral Arteries, Open Approach |
| 041J49H | Bypass Left External Iliac Artery to Right Femoral Artery with Autologous Venous Tissue, Percutaneous Endoscopic Approach |
| 041J49J | Bypass Left External Iliac Artery to Left Femoral Artery with Autologous Venous Tissue, Percutaneous Endoscopic Approach |
| 041J49K | Bypass Left External Iliac Artery to Bilateral Femoral Arteries with Autologous Venous Tissue, Percutaneous Endoscopic Approach |
| 041J4AH | Bypass Left External Iliac Artery to Right Femoral Artery with Autologous Arterial Tissue, Percutaneous Endoscopic Approach |
| 041J4AJ | Bypass Left External Iliac Artery to Left Femoral Artery with Autologous Arterial Tissue, Percutaneous Endoscopic Approach |
| 041J4AK | Bypass Left External Iliac Artery to Bilateral Femoral Arteries with Autologous Arterial Tissue, Percutaneous Endoscopic Approach |
| 041J4JH | Bypass Left External Iliac Artery to Right Femoral Artery with Synthetic Substitute, Percutaneous Endoscopic Approach |
| 041J4JJ | Bypass Left External Iliac Artery to Left Femoral Artery with Synthetic Substitute, Percutaneous Endoscopic Approach |
| 041J4JK | Bypass Left External Iliac Artery to Bilateral Femoral Arteries with Synthetic Substitute, Percutaneous Endoscopic Approach |
| 041J4KH | Bypass Left External Iliac Artery to Right Femoral Artery with Nonautologous Tissue Substitute, Percutaneous Endoscopic Approach |
| 041J4KJ | Bypass Left External Iliac Artery to Left Femoral Artery with Nonautologous Tissue Substitute, Percutaneous Endoscopic Approach |
| 041J4KK | Bypass Left External Iliac Artery to Bilateral Femoral Arteries with Nonautologous Tissue Substitute, Percutaneous Endoscopic Approach |
| 041J4ZH | Bypass Left External Iliac Artery to Right Femoral Artery, Percutaneous Endoscopic Approach |
| 041J4ZJ | Bypass Left External Iliac Artery to Left Femoral Artery, Percutaneous Endoscopic Approach |
| 041J4ZK | Bypass Left External Iliac Artery to Bilateral Femoral Arteries, Percutaneous Endoscopic Approach |
| 041K09H | Bypass Right Femoral Artery to Right Femoral Artery with Autologous Venous Tissue, Open Approach |
| 041K09J | Bypass Right Femoral Artery to Left Femoral Artery with Autologous Venous Tissue, Open Approach |
| 041K09K | Bypass Right Femoral Artery to Bilateral Femoral Arteries with Autologous Venous Tissue, Open Approach |
| 041K09L | Bypass Right Femoral Artery to Popliteal Artery with Autologous Venous Tissue, Open Approach |
| 041K09M | Bypass Right Femoral Artery to Peroneal Artery with Autologous Venous Tissue, Open Approach |
| 041K09N | Bypass Right Femoral Artery to Posterior Tibial Artery with Autologous Venous Tissue, Open Approach |
| 041K09P | Bypass Right Femoral Artery to Foot Artery with Autologous Venous Tissue, Open Approach |
| 041K09Q | Bypass Right Femoral Artery to Lower Extremity Artery with Autologous Venous Tissue, Open Approach |
| 041K09S | Bypass Right Femoral Artery to Lower Extremity Vein with Autologous Venous Tissue, Open Approach |
| 041K0AH | Bypass Right Femoral Artery to Right Femoral Artery with Autologous Arterial Tissue, Open Approach |
| 041K0AJ | Bypass Right Femoral Artery to Left Femoral Artery with Autologous Arterial Tissue, Open Approach |
| 041K0AK | Bypass Right Femoral Artery to Bilateral Femoral Arteries with Autologous Arterial Tissue, Open Approach |
| 041K0AL | Bypass Right Femoral Artery to Popliteal Artery with Autologous Arterial Tissue, Open Approach |
| 041K0AM | Bypass Right Femoral Artery to Peroneal Artery with Autologous Arterial Tissue, Open Approach |
| 041K0AN | Bypass Right Femoral Artery to Posterior Tibial Artery with Autologous Arterial Tissue, Open Approach |
| 041K0AP | Bypass Right Femoral Artery to Foot Artery with Autologous Arterial Tissue, Open Approach |
| 041K0AQ | Bypass Right Femoral Artery to Lower Extremity Artery with Autologous Arterial Tissue, Open Approach |
| 041K0AS | Bypass Right Femoral Artery to Lower Extremity Vein with Autologous Arterial Tissue, Open Approach |
| 041K0JH | Bypass Right Femoral Artery to Right Femoral Artery with Synthetic Substitute, Open Approach |
| 041K0JJ | Bypass Right Femoral Artery to Left Femoral Artery with Synthetic Substitute, Open Approach |
| 041K0JK | Bypass Right Femoral Artery to Bilateral Femoral Arteries with Synthetic Substitute, Open Approach |
| 041K0JL | Bypass Right Femoral Artery to Popliteal Artery with Synthetic Substitute, Open Approach |
| 041K0JM | Bypass Right Femoral Artery to Peroneal Artery with Synthetic Substitute, Open Approach |
| 041K0JN | Bypass Right Femoral Artery to Posterior Tibial Artery with Synthetic Substitute, Open Approach |
| 041K0JP | Bypass Right Femoral Artery to Foot Artery with Synthetic Substitute, Open Approach |
| 041K0JQ | Bypass Right Femoral Artery to Lower Extremity Artery with Synthetic Substitute, Open Approach |
| 041K0JS | Bypass Right Femoral Artery to Lower Extremity Vein with Synthetic Substitute, Open Approach |
| 041K0KH | Bypass Right Femoral Artery to Right Femoral Artery with Nonautologous Tissue Substitute, Open Approach |
| 041K0KJ | Bypass Right Femoral Artery to Left Femoral Artery with Nonautologous Tissue Substitute, Open Approach |
| 041K0KK | Bypass Right Femoral Artery to Bilateral Femoral Arteries with Nonautologous Tissue Substitute, Open Approach |
| 041K0KL | Bypass Right Femoral Artery to Popliteal Artery with Nonautologous Tissue Substitute, Open Approach |
| 041K0KM | Bypass Right Femoral Artery to Peroneal Artery with Nonautologous Tissue Substitute, Open Approach |
| 041K0KN | Bypass Right Femoral Artery to Posterior Tibial Artery with Nonautologous Tissue Substitute, Open Approach |
| 041K0KP | Bypass Right Femoral Artery to Foot Artery with Nonautologous Tissue Substitute, Open Approach |
| 041K0KQ | Bypass Right Femoral Artery to Lower Extremity Artery with Nonautologous Tissue Substitute, Open Approach |
| 041K0KS | Bypass Right Femoral Artery to Lower Extremity Vein with Nonautologous Tissue Substitute, Open Approach |
| 041K0ZH | Bypass Right Femoral Artery to Right Femoral Artery, Open Approach |
| 041K0ZJ | Bypass Right Femoral Artery to Left Femoral Artery, Open Approach |
| 041K0ZK | Bypass Right Femoral Artery to Bilateral Femoral Arteries, Open Approach |
| 041K0ZL | Bypass Right Femoral Artery to Popliteal Artery, Open Approach |
| 041K0ZM | Bypass Right Femoral Artery to Peroneal Artery, Open Approach |
| 041K0ZN | Bypass Right Femoral Artery to Posterior Tibial Artery, Open Approach |
| 041K0ZP | Bypass Right Femoral Artery to Foot Artery, Open Approach |
| 041K0ZQ | Bypass Right Femoral Artery to Lower Extremity Artery, Open Approach |
| 041K0ZS | Bypass Right Femoral Artery to Lower Extremity Vein, Open Approach |
| 041K49H | Bypass Right Femoral Artery to Right Femoral Artery with Autologous Venous Tissue, Percutaneous Endoscopic Approach |
| 041K49J | Bypass Right Femoral Artery to Left Femoral Artery with Autologous Venous Tissue, Percutaneous Endoscopic Approach |
| 041K49K | Bypass Right Femoral Artery to Bilateral Femoral Arteries with Autologous Venous Tissue, Percutaneous Endoscopic Approach |
| 041K49L | Bypass Right Femoral Artery to Popliteal Artery with Autologous Venous Tissue, Percutaneous Endoscopic Approach |
| 041K49M | Bypass Right Femoral Artery to Peroneal Artery with Autologous Venous Tissue, Percutaneous Endoscopic Approach |
| 041K49N | Bypass Right Femoral Artery to Posterior Tibial Artery with Autologous Venous Tissue, Percutaneous Endoscopic Approach |
| 041K49P | Bypass Right Femoral Artery to Foot Artery with Autologous Venous Tissue, Percutaneous Endoscopic Approach |
| 041K49Q | Bypass Right Femoral Artery to Lower Extremity Artery with Autologous Venous Tissue, Percutaneous Endoscopic Approach |
| 041K49S | Bypass Right Femoral Artery to Lower Extremity Vein with Autologous Venous Tissue, Percutaneous Endoscopic Approach |
| 041K4AH | Bypass Right Femoral Artery to Right Femoral Artery with Autologous Arterial Tissue, Percutaneous Endoscopic Approach |
| 041K4AJ | Bypass Right Femoral Artery to Left Femoral Artery with Autologous Arterial Tissue, Percutaneous Endoscopic Approach |
| 041K4AK | Bypass Right Femoral Artery to Bilateral Femoral Arteries with Autologous Arterial Tissue, Percutaneous Endoscopic Approach |
| 041K4AL | Bypass Right Femoral Artery to Popliteal Artery with Autologous Arterial Tissue, Percutaneous Endoscopic Approach |
| 041K4AM | Bypass Right Femoral Artery to Peroneal Artery with Autologous Arterial Tissue, Percutaneous Endoscopic Approach |
| 041K4AN | Bypass Right Femoral Artery to Posterior Tibial Artery with Autologous Arterial Tissue, Percutaneous Endoscopic Approach |
| 041K4AP | Bypass Right Femoral Artery to Foot Artery with Autologous Arterial Tissue, Percutaneous Endoscopic Approach |
| 041K4AQ | Bypass Right Femoral Artery to Lower Extremity Artery with Autologous Arterial Tissue, Percutaneous Endoscopic Approach |
| 041K4AS | Bypass Right Femoral Artery to Lower Extremity Vein with Autologous Arterial Tissue, Percutaneous Endoscopic Approach |
| 041K4JH | Bypass Right Femoral Artery to Right Femoral Artery with Synthetic Substitute, Percutaneous Endoscopic Approach |
| 041K4JJ | Bypass Right Femoral Artery to Left Femoral Artery with Synthetic Substitute, Percutaneous Endoscopic Approach |
| 041K4JK | Bypass Right Femoral Artery to Bilateral Femoral Arteries with Synthetic Substitute, Percutaneous Endoscopic Approach |
| 041K4JL | Bypass Right Femoral Artery to Popliteal Artery with Synthetic Substitute, Percutaneous Endoscopic Approach |
| 041K4JM | Bypass Right Femoral Artery to Peroneal Artery with Synthetic Substitute, Percutaneous Endoscopic Approach |
| 041K4JN | Bypass Right Femoral Artery to Posterior Tibial Artery with Synthetic Substitute, Percutaneous Endoscopic Approach |
| 041K4JP | Bypass Right Femoral Artery to Foot Artery with Synthetic Substitute, Percutaneous Endoscopic Approach |
| 041K4JQ | Bypass Right Femoral Artery to Lower Extremity Artery with Synthetic Substitute, Percutaneous Endoscopic Approach |
| 041K4JS | Bypass Right Femoral Artery to Lower Extremity Vein with Synthetic Substitute, Percutaneous Endoscopic Approach |
| 041K4KH | Bypass Right Femoral Artery to Right Femoral Artery with Nonautologous Tissue Substitute, Percutaneous Endoscopic Approach |
| 041K4KJ | Bypass Right Femoral Artery to Left Femoral Artery with Nonautologous Tissue Substitute, Percutaneous Endoscopic Approach |
| 041K4KK | Bypass Right Femoral Artery to Bilateral Femoral Arteries with Nonautologous Tissue Substitute, Percutaneous Endoscopic Approach |
| 041K4KL | Bypass Right Femoral Artery to Popliteal Artery with Nonautologous Tissue Substitute, Percutaneous Endoscopic Approach |
| 041K4KM | Bypass Right Femoral Artery to Peroneal Artery with Nonautologous Tissue Substitute, Percutaneous Endoscopic Approach |
| 041K4KN | Bypass Right Femoral Artery to Posterior Tibial Artery with Nonautologous Tissue Substitute, Percutaneous Endoscopic Approach |
| 041K4KP | Bypass Right Femoral Artery to Foot Artery with Nonautologous Tissue Substitute, Percutaneous Endoscopic Approach |
| 041K4KQ | Bypass Right Femoral Artery to Lower Extremity Artery with Nonautologous Tissue Substitute, Percutaneous Endoscopic Approach |
| 041K4KS | Bypass Right Femoral Artery to Lower Extremity Vein with Nonautologous Tissue Substitute, Percutaneous Endoscopic Approach |
| 041K4ZH | Bypass Right Femoral Artery to Right Femoral Artery, Percutaneous Endoscopic Approach |
| 041K4ZJ | Bypass Right Femoral Artery to Left Femoral Artery, Percutaneous Endoscopic Approach |
| 041K4ZK | Bypass Right Femoral Artery to Bilateral Femoral Arteries, Percutaneous Endoscopic Approach |
| 041K4ZL | Bypass Right Femoral Artery to Popliteal Artery, Percutaneous Endoscopic Approach |
| 041K4ZM | Bypass Right Femoral Artery to Peroneal Artery, Percutaneous Endoscopic Approach |
| 041K4ZN | Bypass Right Femoral Artery to Posterior Tibial Artery, Percutaneous Endoscopic Approach |
| 041K4ZP | Bypass Right Femoral Artery to Foot Artery, Percutaneous Endoscopic Approach |
| 041K4ZQ | Bypass Right Femoral Artery to Lower Extremity Artery, Percutaneous Endoscopic Approach |
| 041K4ZS | Bypass Right Femoral Artery to Lower Extremity Vein, Percutaneous Endoscopic Approach |
| 041L09H | Bypass Left Femoral Artery to Right Femoral Artery with Autologous Venous Tissue, Open Approach |
| 041L09J | Bypass Left Femoral Artery to Left Femoral Artery with Autologous Venous Tissue, Open Approach |
| 041L09K | Bypass Left Femoral Artery to Bilateral Femoral Arteries with Autologous Venous Tissue, Open Approach |
| 041L09L | Bypass Left Femoral Artery to Popliteal Artery with Autologous Venous Tissue, Open Approach |
| 041L09M | Bypass Left Femoral Artery to Peroneal Artery with Autologous Venous Tissue, Open Approach |
| 041L09N | Bypass Left Femoral Artery to Posterior Tibial Artery with Autologous Venous Tissue, Open Approach |
| 041L09P | Bypass Left Femoral Artery to Foot Artery with Autologous Venous Tissue, Open Approach |
| 041L09Q | Bypass Left Femoral Artery to Lower Extremity Artery with Autologous Venous Tissue, Open Approach |
| 041L09S | Bypass Left Femoral Artery to Lower Extremity Vein with Autologous Venous Tissue, Open Approach |
| 041L0AH | Bypass Left Femoral Artery to Right Femoral Artery with Autologous Arterial Tissue, Open Approach |
| 041L0AJ | Bypass Left Femoral Artery to Left Femoral Artery with Autologous Arterial Tissue, Open Approach |
| 041L0AK | Bypass Left Femoral Artery to Bilateral Femoral Arteries with Autologous Arterial Tissue, Open Approach |
| 041L0AL | Bypass Left Femoral Artery to Popliteal Artery with Autologous Arterial Tissue, Open Approach |
| 041L0AM | Bypass Left Femoral Artery to Peroneal Artery with Autologous Arterial Tissue, Open Approach |
| 041L0AN | Bypass Left Femoral Artery to Posterior Tibial Artery with Autologous Arterial Tissue, Open Approach |
| 041L0AP | Bypass Left Femoral Artery to Foot Artery with Autologous Arterial Tissue, Open Approach |
| 041L0AQ | Bypass Left Femoral Artery to Lower Extremity Artery with Autologous Arterial Tissue, Open Approach |
| 041L0AS | Bypass Left Femoral Artery to Lower Extremity Vein with Autologous Arterial Tissue, Open Approach |
| 041L0JH | Bypass Left Femoral Artery to Right Femoral Artery with Synthetic Substitute, Open Approach |
| 041L0JJ | Bypass Left Femoral Artery to Left Femoral Artery with Synthetic Substitute, Open Approach |
| 041L0JK | Bypass Left Femoral Artery to Bilateral Femoral Arteries with Synthetic Substitute, Open Approach |
| 041L0JL | Bypass Left Femoral Artery to Popliteal Artery with Synthetic Substitute, Open Approach |
| 041L0JM | Bypass Left Femoral Artery to Peroneal Artery with Synthetic Substitute, Open Approach |
| 041L0JN | Bypass Left Femoral Artery to Posterior Tibial Artery with Synthetic Substitute, Open Approach |
| 041L0JP | Bypass Left Femoral Artery to Foot Artery with Synthetic Substitute, Open Approach |
| 041L0JQ | Bypass Left Femoral Artery to Lower Extremity Artery with Synthetic Substitute, Open Approach |
| 041L0JS | Bypass Left Femoral Artery to Lower Extremity Vein with Synthetic Substitute, Open Approach |
| 041L0KH | Bypass Left Femoral Artery to Right Femoral Artery with Nonautologous Tissue Substitute, Open Approach |
| 041L0KJ | Bypass Left Femoral Artery to Left Femoral Artery with Nonautologous Tissue Substitute, Open Approach |
| 041L0KK | Bypass Left Femoral Artery to Bilateral Femoral Arteries with Nonautologous Tissue Substitute, Open Approach |
| 041L0KL | Bypass Left Femoral Artery to Popliteal Artery with Nonautologous Tissue Substitute, Open Approach |
| 041L0KM | Bypass Left Femoral Artery to Peroneal Artery with Nonautologous Tissue Substitute, Open Approach |
| 041L0KN | Bypass Left Femoral Artery to Posterior Tibial Artery with Nonautologous Tissue Substitute, Open Approach |
| 041L0KP | Bypass Left Femoral Artery to Foot Artery with Nonautologous Tissue Substitute, Open Approach |
| 041L0KQ | Bypass Left Femoral Artery to Lower Extremity Artery with Nonautologous Tissue Substitute, Open Approach |
| 041L0KS | Bypass Left Femoral Artery to Lower Extremity Vein with Nonautologous Tissue Substitute, Open Approach |
| 041L0ZH | Bypass Left Femoral Artery to Right Femoral Artery, Open Approach |
| 041L0ZJ | Bypass Left Femoral Artery to Left Femoral Artery, Open Approach |
| 041L0ZK | Bypass Left Femoral Artery to Bilateral Femoral Arteries, Open Approach |
| 041L0ZL | Bypass Left Femoral Artery to Popliteal Artery, Open Approach |
| 041L0ZM | Bypass Left Femoral Artery to Peroneal Artery, Open Approach |
| 041L0ZN | Bypass Left Femoral Artery to Posterior Tibial Artery, Open Approach |
| 041L0ZP | Bypass Left Femoral Artery to Foot Artery, Open Approach |
| 041L0ZQ | Bypass Left Femoral Artery to Lower Extremity Artery, Open Approach |
| 041L0ZS | Bypass Left Femoral Artery to Lower Extremity Vein, Open Approach |
| 041L49H | Bypass Left Femoral Artery to Right Femoral Artery with Autologous Venous Tissue, Percutaneous Endoscopic Approach |
| 041L49J | Bypass Left Femoral Artery to Left Femoral Artery with Autologous Venous Tissue, Percutaneous Endoscopic Approach |
| 041L49K | Bypass Left Femoral Artery to Bilateral Femoral Arteries with Autologous Venous Tissue, Percutaneous Endoscopic Approach |
| 041L49L | Bypass Left Femoral Artery to Popliteal Artery with Autologous Venous Tissue, Percutaneous Endoscopic Approach |
| 041L49M | Bypass Left Femoral Artery to Peroneal Artery with Autologous Venous Tissue, Percutaneous Endoscopic Approach |
| 041L49N | Bypass Left Femoral Artery to Posterior Tibial Artery with Autologous Venous Tissue, Percutaneous Endoscopic Approach |
| 041L49P | Bypass Left Femoral Artery to Foot Artery with Autologous Venous Tissue, Percutaneous Endoscopic Approach |
| 041L49Q | Bypass Left Femoral Artery to Lower Extremity Artery with Autologous Venous Tissue, Percutaneous Endoscopic Approach |
| 041L49S | Bypass Left Femoral Artery to Lower Extremity Vein with Autologous Venous Tissue, Percutaneous Endoscopic Approach |
| 041L4AH | Bypass Left Femoral Artery to Right Femoral Artery with Autologous Arterial Tissue, Percutaneous Endoscopic Approach |
| 041L4AJ | Bypass Left Femoral Artery to Left Femoral Artery with Autologous Arterial Tissue, Percutaneous Endoscopic Approach |
| 041L4AK | Bypass Left Femoral Artery to Bilateral Femoral Arteries with Autologous Arterial Tissue, Percutaneous Endoscopic Approach |
| 041L4AL | Bypass Left Femoral Artery to Popliteal Artery with Autologous Arterial Tissue, Percutaneous Endoscopic Approach |
| 041L4AM | Bypass Left Femoral Artery to Peroneal Artery with Autologous Arterial Tissue, Percutaneous Endoscopic Approach |
| 041L4AN | Bypass Left Femoral Artery to Posterior Tibial Artery with Autologous Arterial Tissue, Percutaneous Endoscopic Approach |
| 041L4AP | Bypass Left Femoral Artery to Foot Artery with Autologous Arterial Tissue, Percutaneous Endoscopic Approach |
| 041L4AQ | Bypass Left Femoral Artery to Lower Extremity Artery with Autologous Arterial Tissue, Percutaneous Endoscopic Approach |
| 041L4AS | Bypass Left Femoral Artery to Lower Extremity Vein with Autologous Arterial Tissue, Percutaneous Endoscopic Approach |
| 041L4JH | Bypass Left Femoral Artery to Right Femoral Artery with Synthetic Substitute, Percutaneous Endoscopic Approach |
| 041L4JJ | Bypass Left Femoral Artery to Left Femoral Artery with Synthetic Substitute, Percutaneous Endoscopic Approach |
| 041L4JK | Bypass Left Femoral Artery to Bilateral Femoral Arteries with Synthetic Substitute, Percutaneous Endoscopic Approach |
| 041L4JL | Bypass Left Femoral Artery to Popliteal Artery with Synthetic Substitute, Percutaneous Endoscopic Approach |
| 041L4JM | Bypass Left Femoral Artery to Peroneal Artery with Synthetic Substitute, Percutaneous Endoscopic Approach |
| 041L4JN | Bypass Left Femoral Artery to Posterior Tibial Artery with Synthetic Substitute, Percutaneous Endoscopic Approach |
| 041L4JP | Bypass Left Femoral Artery to Foot Artery with Synthetic Substitute, Percutaneous Endoscopic Approach |
| 041L4JQ | Bypass Left Femoral Artery to Lower Extremity Artery with Synthetic Substitute, Percutaneous Endoscopic Approach |
| 041L4JS | Bypass Left Femoral Artery to Lower Extremity Vein with Synthetic Substitute, Percutaneous Endoscopic Approach |
| 041L4KH | Bypass Left Femoral Artery to Right Femoral Artery with Nonautologous Tissue Substitute, Percutaneous Endoscopic Approach |
| 041L4KJ | Bypass Left Femoral Artery to Left Femoral Artery with Nonautologous Tissue Substitute, Percutaneous Endoscopic Approach |
| 041L4KK | Bypass Left Femoral Artery to Bilateral Femoral Arteries with Nonautologous Tissue Substitute, Percutaneous Endoscopic Approach |
| 041L4KL | Bypass Left Femoral Artery to Popliteal Artery with Nonautologous Tissue Substitute, Percutaneous Endoscopic Approach |
| 041L4KM | Bypass Left Femoral Artery to Peroneal Artery with Nonautologous Tissue Substitute, Percutaneous Endoscopic Approach |
| 041L4KN | Bypass Left Femoral Artery to Posterior Tibial Artery with Nonautologous Tissue Substitute, Percutaneous Endoscopic Approach |
| 041L4KP | Bypass Left Femoral Artery to Foot Artery with Nonautologous Tissue Substitute, Percutaneous Endoscopic Approach |
| 041L4KQ | Bypass Left Femoral Artery to Lower Extremity Artery with Nonautologous Tissue Substitute, Percutaneous Endoscopic Approach |
| 041L4KS | Bypass Left Femoral Artery to Lower Extremity Vein with Nonautologous Tissue Substitute, Percutaneous Endoscopic Approach |
| 041L4ZH | Bypass Left Femoral Artery to Right Femoral Artery, Percutaneous Endoscopic Approach |
| 041L4ZJ | Bypass Left Femoral Artery to Left Femoral Artery, Percutaneous Endoscopic Approach |
| 041L4ZK | Bypass Left Femoral Artery to Bilateral Femoral Arteries, Percutaneous Endoscopic Approach |
| 041L4ZL | Bypass Left Femoral Artery to Popliteal Artery, Percutaneous Endoscopic Approach |
| 041L4ZM | Bypass Left Femoral Artery to Peroneal Artery, Percutaneous Endoscopic Approach |
| 041L4ZN | Bypass Left Femoral Artery to Posterior Tibial Artery, Percutaneous Endoscopic Approach |
| 041L4ZP | Bypass Left Femoral Artery to Foot Artery, Percutaneous Endoscopic Approach |
| 041L4ZQ | Bypass Left Femoral Artery to Lower Extremity Artery, Percutaneous Endoscopic Approach |
| 041L4ZS | Bypass Left Femoral Artery to Lower Extremity Vein, Percutaneous Endoscopic Approach |
| 041M09L | Bypass Right Popliteal Artery to Popliteal Artery with Autologous Venous Tissue, Open Approach |
| 041M09M | Bypass Right Popliteal Artery to Peroneal Artery with Autologous Venous Tissue, Open Approach |
| 041M09P | Bypass Right Popliteal Artery to Foot Artery with Autologous Venous Tissue, Open Approach |
| 041M09Q | Bypass Right Popliteal Artery to Lower Extremity Artery with Autologous Venous Tissue, Open Approach |
| 041M09S | Bypass Right Popliteal Artery to Lower Extremity Vein with Autologous Venous Tissue, Open Approach |
| 041M0AL | Bypass Right Popliteal Artery to Popliteal Artery with Autologous Arterial Tissue, Open Approach |
| 041M0AM | Bypass Right Popliteal Artery to Peroneal Artery with Autologous Arterial Tissue, Open Approach |
| 041M0AP | Bypass Right Popliteal Artery to Foot Artery with Autologous Arterial Tissue, Open Approach |
| 041M0AQ | Bypass Right Popliteal Artery to Lower Extremity Artery with Autologous Arterial Tissue, Open Approach |
| 041M0AS | Bypass Right Popliteal Artery to Lower Extremity Vein with Autologous Arterial Tissue, Open Approach |
| 041M0JL | Bypass Right Popliteal Artery to Popliteal Artery with Synthetic Substitute, Open Approach |
| 041M0JM | Bypass Right Popliteal Artery to Peroneal Artery with Synthetic Substitute, Open Approach |
| 041M0JP | Bypass Right Popliteal Artery to Foot Artery with Synthetic Substitute, Open Approach |
| 041M0JQ | Bypass Right Popliteal Artery to Lower Extremity Artery with Synthetic Substitute, Open Approach |
| 041M0JS | Bypass Right Popliteal Artery to Lower Extremity Vein with Synthetic Substitute, Open Approach |
| 041M0KL | Bypass Right Popliteal Artery to Popliteal Artery with Nonautologous Tissue Substitute, Open Approach |
| 041M0KM | Bypass Right Popliteal Artery to Peroneal Artery with Nonautologous Tissue Substitute, Open Approach |
| 041M0KP | Bypass Right Popliteal Artery to Foot Artery with Nonautologous Tissue Substitute, Open Approach |
| 041M0KQ | Bypass Right Popliteal Artery to Lower Extremity Artery with Nonautologous Tissue Substitute, Open Approach |
| 041M0KS | Bypass Right Popliteal Artery to Lower Extremity Vein with Nonautologous Tissue Substitute, Open Approach |
| 041M0ZL | Bypass Right Popliteal Artery to Popliteal Artery, Open Approach |
| 041M0ZM | Bypass Right Popliteal Artery to Peroneal Artery, Open Approach |
| 041M0ZP | Bypass Right Popliteal Artery to Foot Artery, Open Approach |
| 041M0ZQ | Bypass Right Popliteal Artery to Lower Extremity Artery, Open Approach |
| 041M0ZS | Bypass Right Popliteal Artery to Lower Extremity Vein, Open Approach |
| 041M49L | Bypass Right Popliteal Artery to Popliteal Artery with Autologous Venous Tissue, Percutaneous Endoscopic Approach |
| 041M49M | Bypass Right Popliteal Artery to Peroneal Artery with Autologous Venous Tissue, Percutaneous Endoscopic Approach |
| 041M49P | Bypass Right Popliteal Artery to Foot Artery with Autologous Venous Tissue, Percutaneous Endoscopic Approach |
| 041M49Q | Bypass Right Popliteal Artery to Lower Extremity Artery with Autologous Venous Tissue, Percutaneous Endoscopic Approach |
| 041M49S | Bypass Right Popliteal Artery to Lower Extremity Vein with Autologous Venous Tissue, Percutaneous Endoscopic Approach |
| 041M4AL | Bypass Right Popliteal Artery to Popliteal Artery with Autologous Arterial Tissue, Percutaneous Endoscopic Approach |
| 041M4AM | Bypass Right Popliteal Artery to Peroneal Artery with Autologous Arterial Tissue, Percutaneous Endoscopic Approach |
| 041M4AP | Bypass Right Popliteal Artery to Foot Artery with Autologous Arterial Tissue, Percutaneous Endoscopic Approach |
| 041M4AQ | Bypass Right Popliteal Artery to Lower Extremity Artery with Autologous Arterial Tissue, Percutaneous Endoscopic Approach |
| 041M4AS | Bypass Right Popliteal Artery to Lower Extremity Vein with Autologous Arterial Tissue, Percutaneous Endoscopic Approach |
| 041M4JL | Bypass Right Popliteal Artery to Popliteal Artery with Synthetic Substitute, Percutaneous Endoscopic Approach |
| 041M4JM | Bypass Right Popliteal Artery to Peroneal Artery with Synthetic Substitute, Percutaneous Endoscopic Approach |
| 041M4JP | Bypass Right Popliteal Artery to Foot Artery with Synthetic Substitute, Percutaneous Endoscopic Approach |
| 041M4JQ | Bypass Right Popliteal Artery to Lower Extremity Artery with Synthetic Substitute, Percutaneous Endoscopic Approach |
| 041M4JS | Bypass Right Popliteal Artery to Lower Extremity Vein with Synthetic Substitute, Percutaneous Endoscopic Approach |
| 041M4KL | Bypass Right Popliteal Artery to Popliteal Artery with Nonautologous Tissue Substitute, Percutaneous Endoscopic Approach |
| 041M4KM | Bypass Right Popliteal Artery to Peroneal Artery with Nonautologous Tissue Substitute, Percutaneous Endoscopic Approach |
| 041M4KP | Bypass Right Popliteal Artery to Foot Artery with Nonautologous Tissue Substitute, Percutaneous Endoscopic Approach |
| 041M4KQ | Bypass Right Popliteal Artery to Lower Extremity Artery with Nonautologous Tissue Substitute, Percutaneous Endoscopic Approach |
| 041M4KS | Bypass Right Popliteal Artery to Lower Extremity Vein with Nonautologous Tissue Substitute, Percutaneous Endoscopic Approach |
| 041M4ZL | Bypass Right Popliteal Artery to Popliteal Artery, Percutaneous Endoscopic Approach |
| 041M4ZM | Bypass Right Popliteal Artery to Peroneal Artery, Percutaneous Endoscopic Approach |
| 041M4ZP | Bypass Right Popliteal Artery to Foot Artery, Percutaneous Endoscopic Approach |
| 041M4ZQ | Bypass Right Popliteal Artery to Lower Extremity Artery, Percutaneous Endoscopic Approach |
| 041M4ZS | Bypass Right Popliteal Artery to Lower Extremity Vein, Percutaneous Endoscopic Approach |
| 041N09L | Bypass Left Popliteal Artery to Popliteal Artery with Autologous Venous Tissue, Open Approach |
| 041N09M | Bypass Left Popliteal Artery to Peroneal Artery with Autologous Venous Tissue, Open Approach |
| 041N09P | Bypass Left Popliteal Artery to Foot Artery with Autologous Venous Tissue, Open Approach |
| 041N09Q | Bypass Left Popliteal Artery to Lower Extremity Artery with Autologous Venous Tissue, Open Approach |
| 041N09S | Bypass Left Popliteal Artery to Lower Extremity Vein with Autologous Venous Tissue, Open Approach |
| 041N0AL | Bypass Left Popliteal Artery to Popliteal Artery with Autologous Arterial Tissue, Open Approach |
| 041N0AM | Bypass Left Popliteal Artery to Peroneal Artery with Autologous Arterial Tissue, Open Approach |
| 041N0AP | Bypass Left Popliteal Artery to Foot Artery with Autologous Arterial Tissue, Open Approach |
| 041N0AQ | Bypass Left Popliteal Artery to Lower Extremity Artery with Autologous Arterial Tissue, Open Approach |
| 041N0AS | Bypass Left Popliteal Artery to Lower Extremity Vein with Autologous Arterial Tissue, Open Approach |
| 041N0JL | Bypass Left Popliteal Artery to Popliteal Artery with Synthetic Substitute, Open Approach |
| 041N0JM | Bypass Left Popliteal Artery to Peroneal Artery with Synthetic Substitute, Open Approach |
| 041N0JP | Bypass Left Popliteal Artery to Foot Artery with Synthetic Substitute, Open Approach |
| 041N0JQ | Bypass Left Popliteal Artery to Lower Extremity Artery with Synthetic Substitute, Open Approach |
| 041N0JS | Bypass Left Popliteal Artery to Lower Extremity Vein with Synthetic Substitute, Open Approach |
| 041N0KL | Bypass Left Popliteal Artery to Popliteal Artery with Nonautologous Tissue Substitute, Open Approach |
| 041N0KM | Bypass Left Popliteal Artery to Peroneal Artery with Nonautologous Tissue Substitute, Open Approach |
| 041N0KP | Bypass Left Popliteal Artery to Foot Artery with Nonautologous Tissue Substitute, Open Approach |
| 041N0KQ | Bypass Left Popliteal Artery to Lower Extremity Artery with Nonautologous Tissue Substitute, Open Approach |
| 041N0KS | Bypass Left Popliteal Artery to Lower Extremity Vein with Nonautologous Tissue Substitute, Open Approach |
| 041N0ZL | Bypass Left Popliteal Artery to Popliteal Artery, Open Approach |
| 041N0ZM | Bypass Left Popliteal Artery to Peroneal Artery, Open Approach |
| 041N0ZP | Bypass Left Popliteal Artery to Foot Artery, Open Approach |
| 041N0ZQ | Bypass Left Popliteal Artery to Lower Extremity Artery, Open Approach |
| 041N0ZS | Bypass Left Popliteal Artery to Lower Extremity Vein, Open Approach |
| 041N49L | Bypass Left Popliteal Artery to Popliteal Artery with Autologous Venous Tissue, Percutaneous Endoscopic Approach |
| 041N49M | Bypass Left Popliteal Artery to Peroneal Artery with Autologous Venous Tissue, Percutaneous Endoscopic Approach |
| 041N49P | Bypass Left Popliteal Artery to Foot Artery with Autologous Venous Tissue, Percutaneous Endoscopic Approach |
| 041N49Q | Bypass Left Popliteal Artery to Lower Extremity Artery with Autologous Venous Tissue, Percutaneous Endoscopic Approach |
| 041N49S | Bypass Left Popliteal Artery to Lower Extremity Vein with Autologous Venous Tissue, Percutaneous Endoscopic Approach |
| 041N4AL | Bypass Left Popliteal Artery to Popliteal Artery with Autologous Arterial Tissue, Percutaneous Endoscopic Approach |
| 041N4AM | Bypass Left Popliteal Artery to Peroneal Artery with Autologous Arterial Tissue, Percutaneous Endoscopic Approach |
| 041N4AP | Bypass Left Popliteal Artery to Foot Artery with Autologous Arterial Tissue, Percutaneous Endoscopic Approach |
| 041N4AQ | Bypass Left Popliteal Artery to Lower Extremity Artery with Autologous Arterial Tissue, Percutaneous Endoscopic Approach |
| 041N4AS | Bypass Left Popliteal Artery to Lower Extremity Vein with Autologous Arterial Tissue, Percutaneous Endoscopic Approach |
| 041N4JL | Bypass Left Popliteal Artery to Popliteal Artery with Synthetic Substitute, Percutaneous Endoscopic Approach |
| 041N4JM | Bypass Left Popliteal Artery to Peroneal Artery with Synthetic Substitute, Percutaneous Endoscopic Approach |
| 041N4JP | Bypass Left Popliteal Artery to Foot Artery with Synthetic Substitute, Percutaneous Endoscopic Approach |
| 041N4JQ | Bypass Left Popliteal Artery to Lower Extremity Artery with Synthetic Substitute, Percutaneous Endoscopic Approach |
| 041N4JS | Bypass Left Popliteal Artery to Lower Extremity Vein with Synthetic Substitute, Percutaneous Endoscopic Approach |
| 041N4KL | Bypass Left Popliteal Artery to Popliteal Artery with Nonautologous Tissue Substitute, Percutaneous Endoscopic Approach |
| 041N4KM | Bypass Left Popliteal Artery to Peroneal Artery with Nonautologous Tissue Substitute, Percutaneous Endoscopic Approach |
| 041N4KP | Bypass Left Popliteal Artery to Foot Artery with Nonautologous Tissue Substitute, Percutaneous Endoscopic Approach |
| 041N4KQ | Bypass Left Popliteal Artery to Lower Extremity Artery with Nonautologous Tissue Substitute, Percutaneous Endoscopic Approach |
| 041N4KS | Bypass Left Popliteal Artery to Lower Extremity Vein with Nonautologous Tissue Substitute, Percutaneous Endoscopic Approach |
| 041N4ZL | Bypass Left Popliteal Artery to Popliteal Artery, Percutaneous Endoscopic Approach |
| 041N4ZM | Bypass Left Popliteal Artery to Peroneal Artery, Percutaneous Endoscopic Approach |
| 041N4ZP | Bypass Left Popliteal Artery to Foot Artery, Percutaneous Endoscopic Approach |
| 041N4ZQ | Bypass Left Popliteal Artery to Lower Extremity Artery, Percutaneous Endoscopic Approach |
| 041N4ZS | Bypass Left Popliteal Artery to Lower Extremity Vein, Percutaneous Endoscopic Approach |
| 04RK07Z | Replacement of Right Femoral Artery with Autologous Tissue Substitute, Open Approach |
| 04RK0JZ | Replacement of Right Femoral Artery with Synthetic Substitute, Open Approach |
| 04RK0KZ | Replacement of Right Femoral Artery with Nonautologous Tissue Substitute, Open Approach |
| 04RK47Z | Replacement of Right Femoral Artery with Autologous Tissue Substitute, Percutaneous Endoscopic Approach |
| 04RK4JZ | Replacement of Right Femoral Artery with Synthetic Substitute, Percutaneous Endoscopic Approach |
| 04RK4KZ | Replacement of Right Femoral Artery with Nonautologous Tissue Substitute, Percutaneous Endoscopic Approach |
| 04RL07Z | Replacement of Left Femoral Artery with Autologous Tissue Substitute, Open Approach |
| 04RL0JZ | Replacement of Left Femoral Artery with Synthetic Substitute, Open Approach |
| 04RL0KZ | Replacement of Left Femoral Artery with Nonautologous Tissue Substitute, Open Approach |
| 04RL47Z | Replacement of Left Femoral Artery with Autologous Tissue Substitute, Percutaneous Endoscopic Approach |
| 04RL4JZ | Replacement of Left Femoral Artery with Synthetic Substitute, Percutaneous Endoscopic Approach |
| 04RL4KZ | Replacement of Left Femoral Artery with Nonautologous Tissue Substitute, Percutaneous Endoscopic Approach |
| 04RM07Z | Replacement of Right Popliteal Artery with Autologous Tissue Substitute, Open Approach |
| 04RM0JZ | Replacement of Right Popliteal Artery with Synthetic Substitute, Open Approach |
| 04RM0KZ | Replacement of Right Popliteal Artery with Nonautologous Tissue Substitute, Open Approach |
| 04RM47Z | Replacement of Right Popliteal Artery with Autologous Tissue Substitute, Percutaneous Endoscopic Approach |
| 04RM4JZ | Replacement of Right Popliteal Artery with Synthetic Substitute, Percutaneous Endoscopic Approach |
| 04RM4KZ | Replacement of Right Popliteal Artery with Nonautologous Tissue Substitute, Percutaneous Endoscopic Approach |
| 04RN07Z | Replacement of Left Popliteal Artery with Autologous Tissue Substitute, Open Approach |
| 04RN0JZ | Replacement of Left Popliteal Artery with Synthetic Substitute, Open Approach |
| 04RN0KZ | Replacement of Left Popliteal Artery with Nonautologous Tissue Substitute, Open Approach |
| 04RN47Z | Replacement of Left Popliteal Artery with Autologous Tissue Substitute, Percutaneous Endoscopic Approach |
| 04RN4JZ | Replacement of Left Popliteal Artery with Synthetic Substitute, Percutaneous Endoscopic Approach |
| 04RN4KZ | Replacement of Left Popliteal Artery with Nonautologous Tissue Substitute, Percutaneous Endoscopic Approach |
| 04RP07Z | Replacement of Right Anterior Tibial Artery with Autologous Tissue Substitute, Open Approach |
| 04RP0JZ | Replacement of Right Anterior Tibial Artery with Synthetic Substitute, Open Approach |
| 04RP0KZ | Replacement of Right Anterior Tibial Artery with Nonautologous Tissue Substitute, Open Approach |
| 04RP47Z | Replacement of Right Anterior Tibial Artery with Autologous Tissue Substitute, Percutaneous Endoscopic Approach |
| 04RP4JZ | Replacement of Right Anterior Tibial Artery with Synthetic Substitute, Percutaneous Endoscopic Approach |
| 04RP4KZ | Replacement of Right Anterior Tibial Artery with Nonautologous Tissue Substitute, Percutaneous Endoscopic Approach |
| 04RQ07Z | Replacement of Left Anterior Tibial Artery with Autologous Tissue Substitute, Open Approach |
| 04RQ0JZ | Replacement of Left Anterior Tibial Artery with Synthetic Substitute, Open Approach |
| 04RQ0KZ | Replacement of Left Anterior Tibial Artery with Nonautologous Tissue Substitute, Open Approach |
| 04RQ47Z | Replacement of Left Anterior Tibial Artery with Autologous Tissue Substitute, Percutaneous Endoscopic Approach |
| 04RQ4JZ | Replacement of Left Anterior Tibial Artery with Synthetic Substitute, Percutaneous Endoscopic Approach |
| 04RQ4KZ | Replacement of Left Anterior Tibial Artery with Nonautologous Tissue Substitute, Percutaneous Endoscopic Approach |
| 04RR07Z | Replacement of Right Posterior Tibial Artery with Autologous Tissue Substitute, Open Approach |
| 04RR0JZ | Replacement of Right Posterior Tibial Artery with Synthetic Substitute, Open Approach |
| 04RR0KZ | Replacement of Right Posterior Tibial Artery with Nonautologous Tissue Substitute, Open Approach |
| 04RR47Z | Replacement of Right Posterior Tibial Artery with Autologous Tissue Substitute, Percutaneous Endoscopic Approach |
| 04RR4JZ | Replacement of Right Posterior Tibial Artery with Synthetic Substitute, Percutaneous Endoscopic Approach |
| 04RR4KZ | Replacement of Right Posterior Tibial Artery with Nonautologous Tissue Substitute, Percutaneous Endoscopic Approach |
| 04RS07Z | Replacement of Left Posterior Tibial Artery with Autologous Tissue Substitute, Open Approach |
| 04RS0JZ | Replacement of Left Posterior Tibial Artery with Synthetic Substitute, Open Approach |
| 04RS0KZ | Replacement of Left Posterior Tibial Artery with Nonautologous Tissue Substitute, Open Approach |
| 04RS47Z | Replacement of Left Posterior Tibial Artery with Autologous Tissue Substitute, Percutaneous Endoscopic Approach |
| 04RS4JZ | Replacement of Left Posterior Tibial Artery with Synthetic Substitute, Percutaneous Endoscopic Approach |
| 04RS4KZ | Replacement of Left Posterior Tibial Artery with Nonautologous Tissue Substitute, Percutaneous Endoscopic Approach |
| 04RT07Z | Replacement of Right Peroneal Artery with Autologous Tissue Substitute, Open Approach |
| 04RT0JZ | Replacement of Right Peroneal Artery with Synthetic Substitute, Open Approach |
| 04RT0KZ | Replacement of Right Peroneal Artery with Nonautologous Tissue Substitute, Open Approach |
| 04RT47Z | Replacement of Right Peroneal Artery with Autologous Tissue Substitute, Percutaneous Endoscopic Approach |
| 04RT4JZ | Replacement of Right Peroneal Artery with Synthetic Substitute, Percutaneous Endoscopic Approach |
| 04RT4KZ | Replacement of Right Peroneal Artery with Nonautologous Tissue Substitute, Percutaneous Endoscopic Approach |
| 04RU07Z | Replacement of Left Peroneal Artery with Autologous Tissue Substitute, Open Approach |
| 04RU0JZ | Replacement of Left Peroneal Artery with Synthetic Substitute, Open Approach |
| 04RU0KZ | Replacement of Left Peroneal Artery with Nonautologous Tissue Substitute, Open Approach |
| 04RU47Z | Replacement of Left Peroneal Artery with Autologous Tissue Substitute, Percutaneous Endoscopic Approach |
| 04RU4JZ | Replacement of Left Peroneal Artery with Synthetic Substitute, Percutaneous Endoscopic Approach |
| 04RU4KZ | Replacement of Left Peroneal Artery with Nonautologous Tissue Substitute, Percutaneous Endoscopic Approach |
| 04RV07Z | Replacement of Right Foot Artery with Autologous Tissue Substitute, Open Approach |
| 04RV0JZ | Replacement of Right Foot Artery with Synthetic Substitute, Open Approach |
| 04RV0KZ | Replacement of Right Foot Artery with Nonautologous Tissue Substitute, Open Approach |
| 04RV47Z | Replacement of Right Foot Artery with Autologous Tissue Substitute, Percutaneous Endoscopic Approach |
| 04RV4JZ | Replacement of Right Foot Artery with Synthetic Substitute, Percutaneous Endoscopic Approach |
| 04RV4KZ | Replacement of Right Foot Artery with Nonautologous Tissue Substitute, Percutaneous Endoscopic Approach |
| 04RW07Z | Replacement of Left Foot Artery with Autologous Tissue Substitute, Open Approach |
| 04RW0JZ | Replacement of Left Foot Artery with Synthetic Substitute, Open Approach |
| 04RW0KZ | Replacement of Left Foot Artery with Nonautologous Tissue Substitute, Open Approach |
| 04RW47Z | Replacement of Left Foot Artery with Autologous Tissue Substitute, Percutaneous Endoscopic Approach |
| 04RW4JZ | Replacement of Left Foot Artery with Synthetic Substitute, Percutaneous Endoscopic Approach |
| 04RW4KZ | Replacement of Left Foot Artery with Nonautologous Tissue Substitute, Percutaneous Endoscopic Approach |
| 04RY07Z | Replacement of Lower Artery with Autologous Tissue Substitute, Open Approach |
| 04RY0JZ | Replacement of Lower Artery with Synthetic Substitute, Open Approach |
| 04RY0KZ | Replacement of Lower Artery with Nonautologous Tissue Substitute, Open Approach |
| 04RY47Z | Replacement of Lower Artery with Autologous Tissue Substitute, Percutaneous Endoscopic Approach |
| 04RY4JZ | Replacement of Lower Artery with Synthetic Substitute, Percutaneous Endoscopic Approach |
| 04RY4KZ | Replacement of Lower Artery with Nonautologous Tissue Substitute, Percutaneous Endoscopic Approach |
| 04UC07Z | Supplement Right Common Iliac Artery with Autologous Tissue Substitute, Open Approach |
| 04UC0JZ | Supplement Right Common Iliac Artery with Synthetic Substitute, Open Approach |
| 04UC0KZ | Supplement Right Common Iliac Artery with Nonautologous Tissue Substitute, Open Approach |
| 04UC37Z | Supplement Right Common Iliac Artery with Autologous Tissue Substitute, Percutaneous Approach |
| 04UC3JZ | Supplement Right Common Iliac Artery with Synthetic Substitute, Percutaneous Approach |
| 04UC3KZ | Supplement Right Common Iliac Artery with Nonautologous Tissue Substitute, Percutaneous Approach |
| 04UC47Z | Supplement Right Common Iliac Artery with Autologous Tissue Substitute, Percutaneous Endoscopic Approach |
| 04UC4JZ | Supplement Right Common Iliac Artery with Synthetic Substitute, Percutaneous Endoscopic Approach |
| 04UC4KZ | Supplement Right Common Iliac Artery with Nonautologous Tissue Substitute, Percutaneous Endoscopic Approach |
| 04UD07Z | Supplement Left Common Iliac Artery with Autologous Tissue Substitute, Open Approach |
| 04UD0JZ | Supplement Left Common Iliac Artery with Synthetic Substitute, Open Approach |
| 04UD0KZ | Supplement Left Common Iliac Artery with Nonautologous Tissue Substitute, Open Approach |
| 04UD37Z | Supplement Left Common Iliac Artery with Autologous Tissue Substitute, Percutaneous Approach |
| 04UD3JZ | Supplement Left Common Iliac Artery with Synthetic Substitute, Percutaneous Approach |
| 04UD3KZ | Supplement Left Common Iliac Artery with Nonautologous Tissue Substitute, Percutaneous Approach |
| 04UD47Z | Supplement Left Common Iliac Artery with Autologous Tissue Substitute, Percutaneous Endoscopic Approach |
| 04UD4JZ | Supplement Left Common Iliac Artery with Synthetic Substitute, Percutaneous Endoscopic Approach |
| 04UD4KZ | Supplement Left Common Iliac Artery with Nonautologous Tissue Substitute, Percutaneous Endoscopic Approach |
| 04UE07Z | Supplement Right Internal Iliac Artery with Autologous Tissue Substitute, Open Approach |
| 04UE0JZ | Supplement Right Internal Iliac Artery with Synthetic Substitute, Open Approach |
| 04UE0KZ | Supplement Right Internal Iliac Artery with Nonautologous Tissue Substitute, Open Approach |
| 04UE37Z | Supplement Right Internal Iliac Artery with Autologous Tissue Substitute, Percutaneous Approach |
| 04UE3JZ | Supplement Right Internal Iliac Artery with Synthetic Substitute, Percutaneous Approach |
| 04UE3KZ | Supplement Right Internal Iliac Artery with Nonautologous Tissue Substitute, Percutaneous Approach |
| 04UE47Z | Supplement Right Internal Iliac Artery with Autologous Tissue Substitute, Percutaneous Endoscopic Approach |
| 04UE4JZ | Supplement Right Internal Iliac Artery with Synthetic Substitute, Percutaneous Endoscopic Approach |
| 04UE4KZ | Supplement Right Internal Iliac Artery with Nonautologous Tissue Substitute, Percutaneous Endoscopic Approach |
| 04UF07Z | Supplement Left Internal Iliac Artery with Autologous Tissue Substitute, Open Approach |
| 04UF0JZ | Supplement Left Internal Iliac Artery with Synthetic Substitute, Open Approach |
| 04UF0KZ | Supplement Left Internal Iliac Artery with Nonautologous Tissue Substitute, Open Approach |
| 04UF37Z | Supplement Left Internal Iliac Artery with Autologous Tissue Substitute, Percutaneous Approach |
| 04UF3JZ | Supplement Left Internal Iliac Artery with Synthetic Substitute, Percutaneous Approach |
| 04UF3KZ | Supplement Left Internal Iliac Artery with Nonautologous Tissue Substitute, Percutaneous Approach |
| 04UF47Z | Supplement Left Internal Iliac Artery with Autologous Tissue Substitute, Percutaneous Endoscopic Approach |
| 04UF4JZ | Supplement Left Internal Iliac Artery with Synthetic Substitute, Percutaneous Endoscopic Approach |
| 04UF4KZ | Supplement Left Internal Iliac Artery with Nonautologous Tissue Substitute, Percutaneous Endoscopic Approach |
| 04UH07Z | Supplement Right External Iliac Artery with Autologous Tissue Substitute, Open Approach |
| 04UH0JZ | Supplement Right External Iliac Artery with Synthetic Substitute, Open Approach |
| 04UH0KZ | Supplement Right External Iliac Artery with Nonautologous Tissue Substitute, Open Approach |
| 04UH37Z | Supplement Right External Iliac Artery with Autologous Tissue Substitute, Percutaneous Approach |
| 04UH3JZ | Supplement Right External Iliac Artery with Synthetic Substitute, Percutaneous Approach |
| 04UH3KZ | Supplement Right External Iliac Artery with Nonautologous Tissue Substitute, Percutaneous Approach |
| 04UH47Z | Supplement Right External Iliac Artery with Autologous Tissue Substitute, Percutaneous Endoscopic Approach |
| 04UH4JZ | Supplement Right External Iliac Artery with Synthetic Substitute, Percutaneous Endoscopic Approach |
| 04UH4KZ | Supplement Right External Iliac Artery with Nonautologous Tissue Substitute, Percutaneous Endoscopic Approach |
| 04UJ07Z | Supplement Left External Iliac Artery with Autologous Tissue Substitute, Open Approach |
| 04UJ0JZ | Supplement Left External Iliac Artery with Synthetic Substitute, Open Approach |
| 04UJ0KZ | Supplement Left External Iliac Artery with Nonautologous Tissue Substitute, Open Approach |
| 04UJ37Z | Supplement Left External Iliac Artery with Autologous Tissue Substitute, Percutaneous Approach |
| 04UJ3JZ | Supplement Left External Iliac Artery with Synthetic Substitute, Percutaneous Approach |
| 04UJ3KZ | Supplement Left External Iliac Artery with Nonautologous Tissue Substitute, Percutaneous Approach |
| 04UJ47Z | Supplement Left External Iliac Artery with Autologous Tissue Substitute, Percutaneous Endoscopic Approach |
| 04UJ4JZ | Supplement Left External Iliac Artery with Synthetic Substitute, Percutaneous Endoscopic Approach |
| 04UJ4KZ | Supplement Left External Iliac Artery with Nonautologous Tissue Substitute, Percutaneous Endoscopic Approach |
| 04UK07Z | Supplement Right Femoral Artery with Autologous Tissue Substitute, Open Approach |
| 04UK0JZ | Supplement Right Femoral Artery with Synthetic Substitute, Open Approach |
| 04UK0KZ | Supplement Right Femoral Artery with Nonautologous Tissue Substitute, Open Approach |
| 04UK37Z | Supplement Right Femoral Artery with Autologous Tissue Substitute, Percutaneous Approach |
| 04UK3JZ | Supplement Right Femoral Artery with Synthetic Substitute, Percutaneous Approach |
| 04UK3KZ | Supplement Right Femoral Artery with Nonautologous Tissue Substitute, Percutaneous Approach |
| 04UK47Z | Supplement Right Femoral Artery with Autologous Tissue Substitute, Percutaneous Endoscopic Approach |
| 04UK4JZ | Supplement Right Femoral Artery with Synthetic Substitute, Percutaneous Endoscopic Approach |
| 04UK4KZ | Supplement Right Femoral Artery with Nonautologous Tissue Substitute, Percutaneous Endoscopic Approach |
| 04UL07Z | Supplement Left Femoral Artery with Autologous Tissue Substitute, Open Approach |
| 04UL0JZ | Supplement Left Femoral Artery with Synthetic Substitute, Open Approach |
| 04UL0KZ | Supplement Left Femoral Artery with Nonautologous Tissue Substitute, Open Approach |
| 04UL37Z | Supplement Left Femoral Artery with Autologous Tissue Substitute, Percutaneous Approach |
| 04UL3JZ | Supplement Left Femoral Artery with Synthetic Substitute, Percutaneous Approach |
| 04UL3KZ | Supplement Left Femoral Artery with Nonautologous Tissue Substitute, Percutaneous Approach |
| 04UL47Z | Supplement Left Femoral Artery with Autologous Tissue Substitute, Percutaneous Endoscopic Approach |
| 04UL4JZ | Supplement Left Femoral Artery with Synthetic Substitute, Percutaneous Endoscopic Approach |
| 04UL4KZ | Supplement Left Femoral Artery with Nonautologous Tissue Substitute, Percutaneous Endoscopic Approach |
| 04UM07Z | Supplement Right Popliteal Artery with Autologous Tissue Substitute, Open Approach |
| 04UM0JZ | Supplement Right Popliteal Artery with Synthetic Substitute, Open Approach |
| 04UM0KZ | Supplement Right Popliteal Artery with Nonautologous Tissue Substitute, Open Approach |
| 04UM37Z | Supplement Right Popliteal Artery with Autologous Tissue Substitute, Percutaneous Approach |
| 04UM3JZ | Supplement Right Popliteal Artery with Synthetic Substitute, Percutaneous Approach |
| 04UM3KZ | Supplement Right Popliteal Artery with Nonautologous Tissue Substitute, Percutaneous Approach |
| 04UM47Z | Supplement Right Popliteal Artery with Autologous Tissue Substitute, Percutaneous Endoscopic Approach |
| 04UM4JZ | Supplement Right Popliteal Artery with Synthetic Substitute, Percutaneous Endoscopic Approach |
| 04UM4KZ | Supplement Right Popliteal Artery with Nonautologous Tissue Substitute, Percutaneous Endoscopic Approach |
| 04UN07Z | Supplement Left Popliteal Artery with Autologous Tissue Substitute, Open Approach |
| 04UN0JZ | Supplement Left Popliteal Artery with Synthetic Substitute, Open Approach |
| 04UN0KZ | Supplement Left Popliteal Artery with Nonautologous Tissue Substitute, Open Approach |
| 04UN37Z | Supplement Left Popliteal Artery with Autologous Tissue Substitute, Percutaneous Approach |
| 04UN3JZ | Supplement Left Popliteal Artery with Synthetic Substitute, Percutaneous Approach |
| 04UN3KZ | Supplement Left Popliteal Artery with Nonautologous Tissue Substitute, Percutaneous Approach |
| 04UN47Z | Supplement Left Popliteal Artery with Autologous Tissue Substitute, Percutaneous Endoscopic Approach |
| 04UN4JZ | Supplement Left Popliteal Artery with Synthetic Substitute, Percutaneous Endoscopic Approach |
| 04UN4KZ | Supplement Left Popliteal Artery with Nonautologous Tissue Substitute, Percutaneous Endoscopic Approach |
| 04UP07Z | Supplement Right Anterior Tibial Artery with Autologous Tissue Substitute, Open Approach |
| 04UP0JZ | Supplement Right Anterior Tibial Artery with Synthetic Substitute, Open Approach |
| 04UP0KZ | Supplement Right Anterior Tibial Artery with Nonautologous Tissue Substitute, Open Approach |
| 04UP37Z | Supplement Right Anterior Tibial Artery with Autologous Tissue Substitute, Percutaneous Approach |
| 04UP3JZ | Supplement Right Anterior Tibial Artery with Synthetic Substitute, Percutaneous Approach |
| 04UP3KZ | Supplement Right Anterior Tibial Artery with Nonautologous Tissue Substitute, Percutaneous Approach |
| 04UP47Z | Supplement Right Anterior Tibial Artery with Autologous Tissue Substitute, Percutaneous Endoscopic Approach |
| 04UP4JZ | Supplement Right Anterior Tibial Artery with Synthetic Substitute, Percutaneous Endoscopic Approach |
| 04UP4KZ | Supplement Right Anterior Tibial Artery with Nonautologous Tissue Substitute, Percutaneous Endoscopic Approach |
| 04UQ07Z | Supplement Left Anterior Tibial Artery with Autologous Tissue Substitute, Open Approach |
| 04UQ0JZ | Supplement Left Anterior Tibial Artery with Synthetic Substitute, Open Approach |
| 04UQ0KZ | Supplement Left Anterior Tibial Artery with Nonautologous Tissue Substitute, Open Approach |
| 04UQ37Z | Supplement Left Anterior Tibial Artery with Autologous Tissue Substitute, Percutaneous Approach |
| 04UQ3JZ | Supplement Left Anterior Tibial Artery with Synthetic Substitute, Percutaneous Approach |
| 04UQ3KZ | Supplement Left Anterior Tibial Artery with Nonautologous Tissue Substitute, Percutaneous Approach |
| 04UQ47Z | Supplement Left Anterior Tibial Artery with Autologous Tissue Substitute, Percutaneous Endoscopic Approach |
| 04UQ4JZ | Supplement Left Anterior Tibial Artery with Synthetic Substitute, Percutaneous Endoscopic Approach |
| 04UQ4KZ | Supplement Left Anterior Tibial Artery with Nonautologous Tissue Substitute, Percutaneous Endoscopic Approach |
| 04UR07Z | Supplement Right Posterior Tibial Artery with Autologous Tissue Substitute, Open Approach |
| 04UR0JZ | Supplement Right Posterior Tibial Artery with Synthetic Substitute, Open Approach |
| 04UR0KZ | Supplement Right Posterior Tibial Artery with Nonautologous Tissue Substitute, Open Approach |
| 04UR37Z | Supplement Right Posterior Tibial Artery with Autologous Tissue Substitute, Percutaneous Approach |
| 04UR3JZ | Supplement Right Posterior Tibial Artery with Synthetic Substitute, Percutaneous Approach |
| 04UR3KZ | Supplement Right Posterior Tibial Artery with Nonautologous Tissue Substitute, Percutaneous Approach |
| 04UR47Z | Supplement Right Posterior Tibial Artery with Autologous Tissue Substitute, Percutaneous Endoscopic Approach |
| 04UR4JZ | Supplement Right Posterior Tibial Artery with Synthetic Substitute, Percutaneous Endoscopic Approach |
| 04UR4KZ | Supplement Right Posterior Tibial Artery with Nonautologous Tissue Substitute, Percutaneous Endoscopic Approach |
| 04US07Z | Supplement Left Posterior Tibial Artery with Autologous Tissue Substitute, Open Approach |
| 04US0JZ | Supplement Left Posterior Tibial Artery with Synthetic Substitute, Open Approach |
| 04US0KZ | Supplement Left Posterior Tibial Artery with Nonautologous Tissue Substitute, Open Approach |
| 04US37Z | Supplement Left Posterior Tibial Artery with Autologous Tissue Substitute, Percutaneous Approach |
| 04US3JZ | Supplement Left Posterior Tibial Artery with Synthetic Substitute, Percutaneous Approach |
| 04US3KZ | Supplement Left Posterior Tibial Artery with Nonautologous Tissue Substitute, Percutaneous Approach |
| 04US47Z | Supplement Left Posterior Tibial Artery with Autologous Tissue Substitute, Percutaneous Endoscopic Approach |
| 04US4JZ | Supplement Left Posterior Tibial Artery with Synthetic Substitute, Percutaneous Endoscopic Approach |
| 04US4KZ | Supplement Left Posterior Tibial Artery with Nonautologous Tissue Substitute, Percutaneous Endoscopic Approach |
| 04UT07Z | Supplement Right Peroneal Artery with Autologous Tissue Substitute, Open Approach |
| 04UT0JZ | Supplement Right Peroneal Artery with Synthetic Substitute, Open Approach |
| 04UT0KZ | Supplement Right Peroneal Artery with Nonautologous Tissue Substitute, Open Approach |
| 04UT37Z | Supplement Right Peroneal Artery with Autologous Tissue Substitute, Percutaneous Approach |
| 04UT3JZ | Supplement Right Peroneal Artery with Synthetic Substitute, Percutaneous Approach |
| 04UT3KZ | Supplement Right Peroneal Artery with Nonautologous Tissue Substitute, Percutaneous Approach |
| 04UT47Z | Supplement Right Peroneal Artery with Autologous Tissue Substitute, Percutaneous Endoscopic Approach |
| 04UT4JZ | Supplement Right Peroneal Artery with Synthetic Substitute, Percutaneous Endoscopic Approach |
| 04UT4KZ | Supplement Right Peroneal Artery with Nonautologous Tissue Substitute, Percutaneous Endoscopic Approach |
| 04UU07Z | Supplement Left Peroneal Artery with Autologous Tissue Substitute, Open Approach |
| 04UU0JZ | Supplement Left Peroneal Artery with Synthetic Substitute, Open Approach |
| 04UU0KZ | Supplement Left Peroneal Artery with Nonautologous Tissue Substitute, Open Approach |
| 04UU37Z | Supplement Left Peroneal Artery with Autologous Tissue Substitute, Percutaneous Approach |
| 04UU3JZ | Supplement Left Peroneal Artery with Synthetic Substitute, Percutaneous Approach |
| 04UU3KZ | Supplement Left Peroneal Artery with Nonautologous Tissue Substitute, Percutaneous Approach |
| 04UU47Z | Supplement Left Peroneal Artery with Autologous Tissue Substitute, Percutaneous Endoscopic Approach |
| 04UU4JZ | Supplement Left Peroneal Artery with Synthetic Substitute, Percutaneous Endoscopic Approach |
| 04UU4KZ | Supplement Left Peroneal Artery with Nonautologous Tissue Substitute, Percutaneous Endoscopic Approach |
| 04UV07Z | Supplement Right Foot Artery with Autologous Tissue Substitute, Open Approach |
| 04UV0JZ | Supplement Right Foot Artery with Synthetic Substitute, Open Approach |
| 04UV0KZ | Supplement Right Foot Artery with Nonautologous Tissue Substitute, Open Approach |
| 04UV37Z | Supplement Right Foot Artery with Autologous Tissue Substitute, Percutaneous Approach |
| 04UV3JZ | Supplement Right Foot Artery with Synthetic Substitute, Percutaneous Approach |
| 04UV3KZ | Supplement Right Foot Artery with Nonautologous Tissue Substitute, Percutaneous Approach |
| 04UV47Z | Supplement Right Foot Artery with Autologous Tissue Substitute, Percutaneous Endoscopic Approach |
| 04UV4JZ | Supplement Right Foot Artery with Synthetic Substitute, Percutaneous Endoscopic Approach |
| 04UV4KZ | Supplement Right Foot Artery with Nonautologous Tissue Substitute, Percutaneous Endoscopic Approach |
| 04UW07Z | Supplement Left Foot Artery with Autologous Tissue Substitute, Open Approach |
| 04UW0JZ | Supplement Left Foot Artery with Synthetic Substitute, Open Approach |
| 04UW0KZ | Supplement Left Foot Artery with Nonautologous Tissue Substitute, Open Approach |
| 04UW37Z | Supplement Left Foot Artery with Autologous Tissue Substitute, Percutaneous Approach |
| 04UW3JZ | Supplement Left Foot Artery with Synthetic Substitute, Percutaneous Approach |
| 04UW3KZ | Supplement Left Foot Artery with Nonautologous Tissue Substitute, Percutaneous Approach |
| 04UW47Z | Supplement Left Foot Artery with Autologous Tissue Substitute, Percutaneous Endoscopic Approach |
| 04UW4JZ | Supplement Left Foot Artery with Synthetic Substitute, Percutaneous Endoscopic Approach |
| 04UW4KZ | Supplement Left Foot Artery with Nonautologous Tissue Substitute, Percutaneous Endoscopic Approach |
| 04UY07Z | Supplement Lower Artery with Autologous Tissue Substitute, Open Approach |
| 04UY0JZ | Supplement Lower Artery with Synthetic Substitute, Open Approach |
| 04UY0KZ | Supplement Lower Artery with Nonautologous Tissue Substitute, Open Approach |
| 04UY37Z | Supplement Lower Artery with Autologous Tissue Substitute, Percutaneous Approach |
| 04UY3JZ | Supplement Lower Artery with Synthetic Substitute, Percutaneous Approach |
| 04UY3KZ | Supplement Lower Artery with Nonautologous Tissue Substitute, Percutaneous Approach |
| 04UY47Z | Supplement Lower Artery with Autologous Tissue Substitute, Percutaneous Endoscopic Approach |
| 04UY4JZ | Supplement Lower Artery with Synthetic Substitute, Percutaneous Endoscopic Approach |
| 04UY4KZ | Supplement Lower Artery with Nonautologous Tissue Substitute, Percutaneous Endoscopic Approach |
| B40F0ZZ | Plain Radiography of Right Lower Extremity Arteries using High Osmolar Contrast |
| B40F1ZZ | Plain Radiography of Right Lower Extremity Arteries using Low Osmolar Contrast |
| B40FYZZ | Plain Radiography of Right Lower Extremity Arteries using Other Contrast |
| B40G0ZZ | Plain Radiography of Left Lower Extremity Arteries using High Osmolar Contrast |
| B40G1ZZ | Plain Radiography of Left Lower Extremity Arteries using Low Osmolar Contrast |
| B40GYZZ | Plain Radiography of Left Lower Extremity Arteries using Other Contrast |
| B40J0ZZ | Plain Radiography of Other Lower Arteries using High Osmolar Contrast |
| B40J1ZZ | Plain Radiography of Other Lower Arteries using Low Osmolar Contrast |
| B40JYZZ | Plain Radiography of Other Lower Arteries using Other Contrast |
| B41F0ZZ | Fluoroscopy of Right Lower Extremity Arteries using High Osmolar Contrast |
| B41F1ZZ | Fluoroscopy of Right Lower Extremity Arteries using Low Osmolar Contrast |
| B41FYZZ | Fluoroscopy of Right Lower Extremity Arteries using Other Contrast |
| B41G0ZZ | Fluoroscopy of Left Lower Extremity Arteries using High Osmolar Contrast |
| B41G1ZZ | Fluoroscopy of Left Lower Extremity Arteries using Low Osmolar Contrast |
| B41GYZZ | Fluoroscopy of Left Lower Extremity Arteries using Other Contrast |
| B41J0ZZ | Fluoroscopy of Other Lower Arteries using High Osmolar Contrast |
| B41J1ZZ | Fluoroscopy of Other Lower Arteries using Low Osmolar Contrast |
| B41JYZZ | Fluoroscopy of Other Lower Arteries using Other Contrast |
