# Supplementary material for: Model-Based Algorithms for Detecting Peripheral Artery Disease Using Administrative Data From an Electronic Health Record Data System: Algorithm Development Study
Source: JMIR Med Inform. 2020 Aug 19;8(8):e18542. doi: 10.2196/18542 (PMC7468640; doi:10.2196/18542)
Supplement: Multimedia Appendix 3 [file medinform_v8i8e18542_app3.docx]

**Appendix 3: Baseline characteristics by Training or Validation role.**

| **Variable** | **Validation (N=790)** | **Training (N=1,604)** | **p-value** |
| --- | --- | --- | --- |
| Demographics |  |  |  |
| Age (years), Mean (SD) | 68.0 (14.2) | 67.8 (13.7) | .61 |
| Gender, Male | 417 (52.8%) | 871 (54.3%) | .48 |
| Race |  |  | .73 |
| White | 531 (67.2%) | 1,094 (68.2%) |  |
| Black/African-American | 216 (27.3%) | 416 (25.9%) |  |
| Other Races | 43 (5.4%) | 94 (5.9%) |  |
| Confirmed PAD | 258 (32.7%) | 522 (32.5%) | .96 |
